# New Insights into the RNA-Based Mechanism of Action of the Anticancer Drug 5′-Fluorouracil in Eukaryotic Cells

**DOI:** 10.1371/journal.pone.0078172

**Published:** 2013-11-01

**Authors:** Laura Mojardín, Javier Botet, Luis Quintales, Sergio Moreno, Margarita Salas

**Affiliations:** 1 Instituto de Biología Molecular “Eladio Viñuela” (CSIC), Centro de Biología Molecular “Severo Ochoa” (CSIC-UAM), Universidad Autónoma, Cantoblanco, Madrid, Spain; 2 Instituto de Biología Funcional y Genómica (CSIC/Universidad de Salamanca), Salamanca, Spain; University of Quebect at Trois-Rivieres, Canada

## Abstract

5-Fluorouracil (5FU) is a chemotherapeutic drug widely used in treating a range of advanced, solid tumours and, in particular, colorectal cancer. Here, we used high-density tiling DNA microarray technology to obtain the specific transcriptome-wide response induced by 5FU in the eukaryotic model *Schizosaccharomyces pombe*. This approach combined with real-time quantitative PCR analysis allowed us to detect splicing defects of a significant number of intron-containing mRNA, in addition to identify some rRNA and tRNA processing defects after 5FU treatment. Interestingly, our studies also revealed that 5FU specifically induced the expression of certain genes implicated in the processing of mRNA, tRNA and rRNA precursors, and in the post-transcriptional modification of uracil residues in RNA. The transcription of several tRNA genes was also significantly induced after drug exposure. These transcriptional changes might represent a cellular response mechanism to counteract 5FU damage since deletion strains for some of these up-regulated genes were hypersensitive to 5FU. Moreover, most of these RNA processing genes have human orthologs that participate in conserved pathways, suggesting that they could be novel targets to improve the efficacy of 5FU-based treatments.

## Introduction

The antimetabolite 5-Fluorouracil (5FU) is an analogue of uracil widely used as a chemotherapeutic agent in the treatment of a variety of cancers. 5FU-based therapy has been shown to significantly increase both the response and survival rates for breast, neck and head cancer; however, its effectiveness is higher in advanced colorectal tumours [1]. An understanding of its mechanism of action is fundamental to be able to enhance the clinical effectiveness of chemotherapy by rationally designing new strategies as well as to enhance our knowledge of the cytotoxic effects, which have been associated with its active metabolites. In particular, the fluorodeoxyuridine monophosphate (FdUMP) is known to inhibit the thymidylate synthase causing deoxynucleotide pool imbalances and DNA damage [2], whereas the fluorodeoxyuridine triphosphate (FdUTP) and the fluorouridine triphosphate (FUTP) can be misincorporated into DNA and RNA, respectively, interfering with normal nucleic acid metabolism [3]–[5]. However, there is growing evidence that 5FU cytotoxicity may mainly be attributable to the impairment of RNA processing pathways. Accordingly, several experiments have shown that uridine, but not thymidine, relieved the cytotoxic and apoptotic effects of 5FU [1], [6]. 5FU-containing RNA has been shown to interfere with normal processing and maturation of rRNA, tRNA, and mRNA precursors [1], [4], [7]–[9]. Although a well-established correlation exists between the direct incorporation of 5FU into RNA and the cytotoxic effects of the drug, the mechanism underlying this toxicity has not been fully elucidated.

To gain further insights into the *in vivo* mechanism of action by which 5FU causes RNA-based toxicity in eukaryotic cells, we used the fission yeast *Schizosaccharomyces pombe* as a model organism. As many essential cellular processes are conserved in eukaryotes, yeast species have been proven to be powerful tools for identifying human drug targets [10], [11]. We employed high-density tiling DNA microarray technology to perform the first whole genome transcriptional profile associated with the 5FU response in a eukaryotic organism. Combining this strategy with real-time quantitative PCR (qPCR) experiments, we were able to identify a range of processing defects of mRNA, tRNA and rRNA precursors caused by 5FU treatment. The significant induction of certain RNA processing genes might be associated with these drug effects as a cellular response mechanism to counteract 5FU damage.

## Materials and Methods

### Chemicals, Yeast Strains and Growth Media

5FU was obtained from Sigma-Aldrich (St. Louis, MO; Cat. No. F6627) prepared as a 20 mM stock solution in water and kept at 4°C. The *S. pombe* strains used in this study are listed in Table S1. Haploid deletion mutants used in this study were purchased from Bioneer. Cells were grown in YE (3% glucose, 0.5% yeast extract).

### Fluorescence-activated Cell Sorting Analysis

Flow cytometry was used to estimate the relative DNA content of fission yeast cells at 0, 15, 60 or 240 minutes after 5FU treatment. Approximately 10^7^ cells from an exponentially growing culture were collected by centrifugation, fixed in 70% ethanol, and processed as previously described [12]. Analysis was performed using FACSCalibur (Becton Dickinson) and CELLQuest software.

### 4′, 6′-Diamidino-2-phenylindole Staining and Microscopy

Ethanol-fixed cells were washed once in buffer PBS (137 mM NaCl, 2.7 mM KCl, 10 mM Na_2_HPO_4_ and 2 mM KH_2_PO_4_, pH 7.4) and then stained with 4′,6′-diamidino-2-phenylindole (DAPI, Merck) at a final concentration of 1 µg/mL. Images were acquired on a laser-scanning confocal microscope (LSM510 Meta; Carl Zeiss) equipped with an Axiovert 200 M.

### Viability Assays

For liquid survival assays, 5FU was added to early exponentially growing cells (OD_595_ = 0.2, ∼4×10^6^ cells/ml). After incubation for the indicated times, cells were plated in rich media (YE) and colonies were counted after incubation during 3–4 days at 30°C. For growth inhibition assays, strains were inoculated in triplicates in a 96-well plate containing YE and grown at 30°C to saturation. Then, they were replicated into 96-well plates with YE medium (with or without 150 µM 5FU) using a stainless steel 96-pin replicator (Nalgene Nunc International) and incubated at 30°C. Growth was quantitatively scored every 24 h by monitoring the absorbance at 595 nm with a microplate reader (Varioskan, Thermo Scientific).

### Total RNA Extraction

Cultures of *S. pombe* wild-type strain 972 h^−^ were grown in YE medium at 30°C to OD_595_ = 0.2 (∼4×10^6^ cells/ml). 5FU was added to the cultures except controls to a final concentration of 500 µM and incubation was allowed to proceed for 15, 60 or 240 minutes. Total RNA was extracted using the MasterPure Yeast RNA Purification Kit (Epicentre, Madison, WI) according to the manufacturer’s recommendations. The purified RNA was immediately frozen in liquid nitrogen and stored at −70°C. The quality and quantity of total RNA were determined by Nanodrop ND-1000 UV spectroscopy (Thermo Scientific), and RNA integrity was checked using a 2100 Bioanalyzer (Agilent Technologies). We obtained high-quality RNA from all the samples since the RNA Integrity Number (RIN) was greater than 9 in each case.

### Target Labelling and Microarray Hybridization

Affymetrix GeneChip *S. pombe* 1.0FR tiling microarray containing 25-mer probes tiled at 20-nucleotide intervals across both strands of the fission yeast genome were used for measurement of DNA strand-specific expression at 0, 15, 60 or 240 minutes of 5FU exposure in two independent biological replicates. Labelling and hybridizations were performed according to protocols from Affymetrix. Briefly, 300 ng of total RNA were amplified and labelled (preserving the original polarity of the RNA) using the GeneChip whole transcript sense target labelling assay and then hybridized to *S. pombe* tiling 1.0 FR Array (Affymetrix). Washing and scanning were performed using GeneChip System of Affymetrix (GeneChip Hybridization Oven 640, GeneChip Fluidics Station 450 and GeneChip Scanner 7G). The Pearson correlation coefficients of the probe hybridization signals between tiling microarray duplicates were 0.982 (t0), 0.978 (t15), 0.974 (t60) and 0.985 (t240) indicating minimum variability between them. The complete set of microarray hybridization results is available at the Gene Expression Omnibus (GEO) database under accession number GSE46919.

### Differential Expression Analysis of Microarray Data

To obtain a quantification of the differential gene expression pattern we employed a previously reported protocol [13], which processes the strand-specific hybridization signals in a quantitative manner to create a custom Chip Description File (CDF) based on the PomBase annotation database [14] ftp://ftp.ebi.ac.uk/pub/databases/pombase/pombe/Chromosome_contigs/OLD/20110204). Transcriptional levels were calculated after background correction, normalization and summarization using RMA Bioconductor package [15]. Differential expression data and the associated P values were adjusted for multiple testing using the Benjamini and Hochberg's method. Limma Bioconductor package was used to control the false discovery rate [16]. To validate the gene expression results obtained from the microarray analysis, we randomly picked out two RNA processing genes (*ctu1* and *SPBC713.05*) and two genes (*ssa1* and *psi1*) whose human orthologs were induced after 5FU exposure in cell cultures [17], [18]. The qPCR analysis confirmed the transcriptional changes detected from microarrays (Figure S1). Primers for amplification reactions are listed in Table S2.

### Synthesis of cDNA for qPCR Analysis

The reverse transcription of RNA to further check the levels of certain mRNA or rRNA regions by qPCR was performed using the High Capacity RNA-to-cDNA Master Mix (Applied Biosystems, Cat. No. 4390712) according to the manufacturer’s instructions using 500 ng of total RNA. Samples omitting reverse transcriptase were included as negative controls in each set of reactions. PCR primers for reverse transcription reactions (Table S2) were designed using Primer-BLAST software (http://www.ncbi.nlm.nih.gov/tools/primer-blast/). The specificity of each primer pair was assessed *in silico* by matching them against the complete *S. pombe* genome. To analyse the tRNA levels, we first synthesized cDNA products from total RNA as follows: RNA was denatured at 90°C for 5 min and then snap-cooled on ice for 10 min. Then, 600 ng of total RNA was reverse transcribed with Super Script III First-Strand Synthesis SuperMix for qRT-PCR (Invitrogen, Cat. No.11752) according to the manufacturer’s recommendations except that the incubation reaction was performed at 54°C to facilitate transcription through the highly stable secondary structure of the tRNA. PCR primers to amplify intronic or exonic regions within tRNA were manually designed (Table S2). Despite the fact that many tRNA genes share sequence similarity, primers to amplify intronic or exonic regions within the selected tRNA genes were all specific for those regions as we only detected a unique PCR product using melting curve analysis. Note that genes for tRNA^Ala^_CGC_, tRNA^Arg^_CCU_ and tRNA^Gly^_CCC_ only have one copy in the genome, whereas several identical copies are found for tRNA^Val^_UAC_, tRNA^Ser^_GCU_, tRNA^Trp^_CCA_, tRNA^Leu^_CAA_, tRNA^His^_GUG_ and tRNA^Glu^_CUC_ genes. Although many tRNA molecules bearing the same anticodon do not have exactly the same sequence, we only analysed those with identical sequences.

### Real-Time Quantitative PCR Assays

Absolute quantification of cDNA derived from mRNA and rRNA were performed in triplicates on a BioRad CFX 384 instrument with 10 µL of final reaction volume containing 5 ng of cDNA (except for rDNA regions in which cDNA was diluted to 0.05 ng), 2.5 µM of each forward and reverse primer, and 5 µL of Fast Sybr Green Master Mix (Applied Biosystems). The qPCR assays were set up using an Eppendorf pipetting robot (epMotion 5075). PCR efficiency for each primer pair was calculated using 4-fold dilutions (from 50 ng) of pooled cDNA. Cycling parameters were as follows: 20 sec at 95°C followed by 40 two-step cycles of 95°C for 3 sec +60°C for 30 sec. Melting curve analysis from 60°C to 95°C was included at the end of the program. Essentially, the same protocol was used to obtain the relative quantification of tRNA levels except for the amount of cDNA added to the amplification reactions (1 ng) and the cycling conditions: 5 sec at 95°C followed by 40 two-step cycles of 95°C for 5 sec +62°C for 5 sec. Data analysis was performed using GenEx qPCR data analysis software v.5.3.7 (MultiD).

### Qualitative RT-PCR and Gel Electrophoresis

To visualize the presence of unspliced transcripts we performed qualitative RT-PCR assays in a Biorad Thermal Cycler. The amplification reactions contained 10 ng of cDNA, 1 U of Vent DNA Polymerase (New England BioLabs), 2.5 µL of 10× ThermoPol Reaction Buffer, 1 µM of each forward and reverse primer (Table S2), 150 µM of each dNTP and 1 µCi of [α-^32^P]-CTP (3000 Ci/mmol) (Perkin-Elmer Life Science) in a final volume of 25 µL. Cycling conditions were as follows: 92°C for 4 min, followed by 22 three-step cycles at 92°C for 45 sec +50°C for 60 sec and 72°C for 80 sec and a final elongation step at 72°C for 10 min. Then the PCR products were analysed by agarose gel electrophoresis. A 1 Kb Plus DNA Ladder (Invitrogen) was used to determine products size. Electrophoresis was carried out at room temperature in TAE buffer (40 mM Tris, 1 mM EDTA, pH set to 7.5 with acetic acid) at 120 V for 40 min. Gels were vacuum-dried and the radioactive bands were detected by autoradiography.

## Results and Discussion

### 5FU Induces Cytotoxicity in *S. pombe*

**Figure 1:**
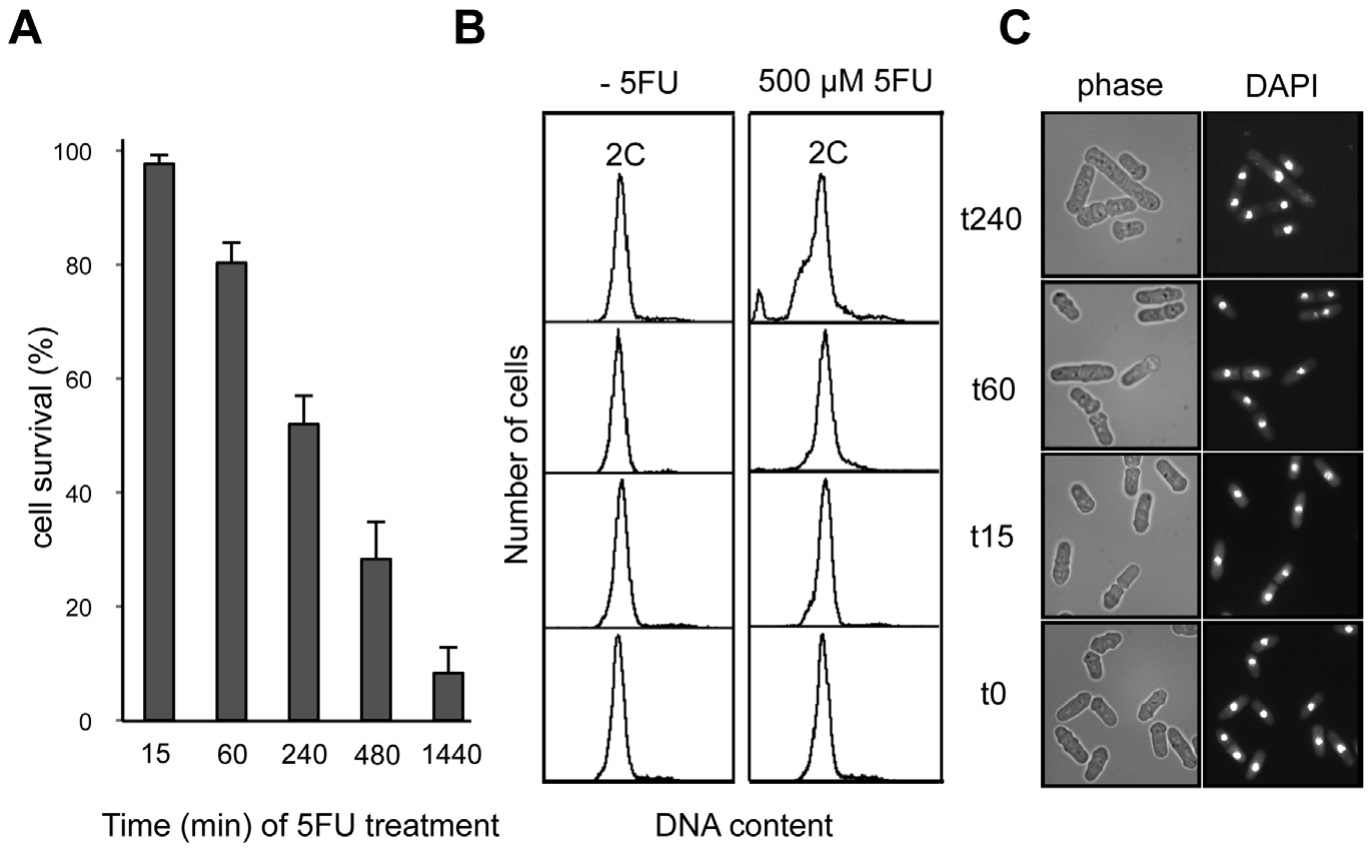
Phenotypic effects of 5FU in *S. pombe* cells. (**A**) Survival time course after 500 µM 5FU (EC50) treatment. The values represent the means ± s.d. of three independent experiments. (**B**) FACS analysis of the time course experiment indicating heterogeneity in DNA content at 240 min in 5FU-treated cells compared to untreated cells. (**C**) Microscopy analysis and DAPI staining show cells with elongated phenotype after 5FU exposure for 240 min suggesting a cell cycle delay in the presence of the drug.

We performed a time course analysis of *S. pombe* viability after incubating cells with 500 µM 5FU (the half maximal effective concentration dose, EC50, at 240 min) in order to evaluate the phenotypic effects associated to drug treatment (Figure 1A). Flow cytometry experiments revealed that 5FU-treated cells showed an abnormal DNA content at 240 min when compared to untreated cells (Figure 1B), probably due to a delayed entry into S phase. Consistent with this result, 5FU has been described as a G1/S phase blocker in human cell lines [19]. In addition, microscopy analysis and nuclear DAPI staining showed that a proportion of the cells treated with 5FU for 240 min became elongated with a characteristic cell cycle delay phenotype (Figure 1C).

### Global Disruption of Pre-mRNA Splicing Caused by 5FU

Despite increasing evidence that suggests that 5FU mainly exerts its cytotoxic effects through the inhibition of RNA metabolism, little is known about the global effects caused by 5FU in RNA processing and particularly in mRNA splicing. In order to gain further insights into the transcriptome-wide impact of 5FU, we took advantage of high-density tiling microarray technology for detecting intron-containing transcripts in *S. pombe*. A time-course and high-resolution survey of all differentially transcribed regions of the genome were obtained by comparing the transcriptional response of cells exposed to 500 µM of 5FU during 15, 60 or 240 minutes with respect to an untreated control.

**Figure 2:**
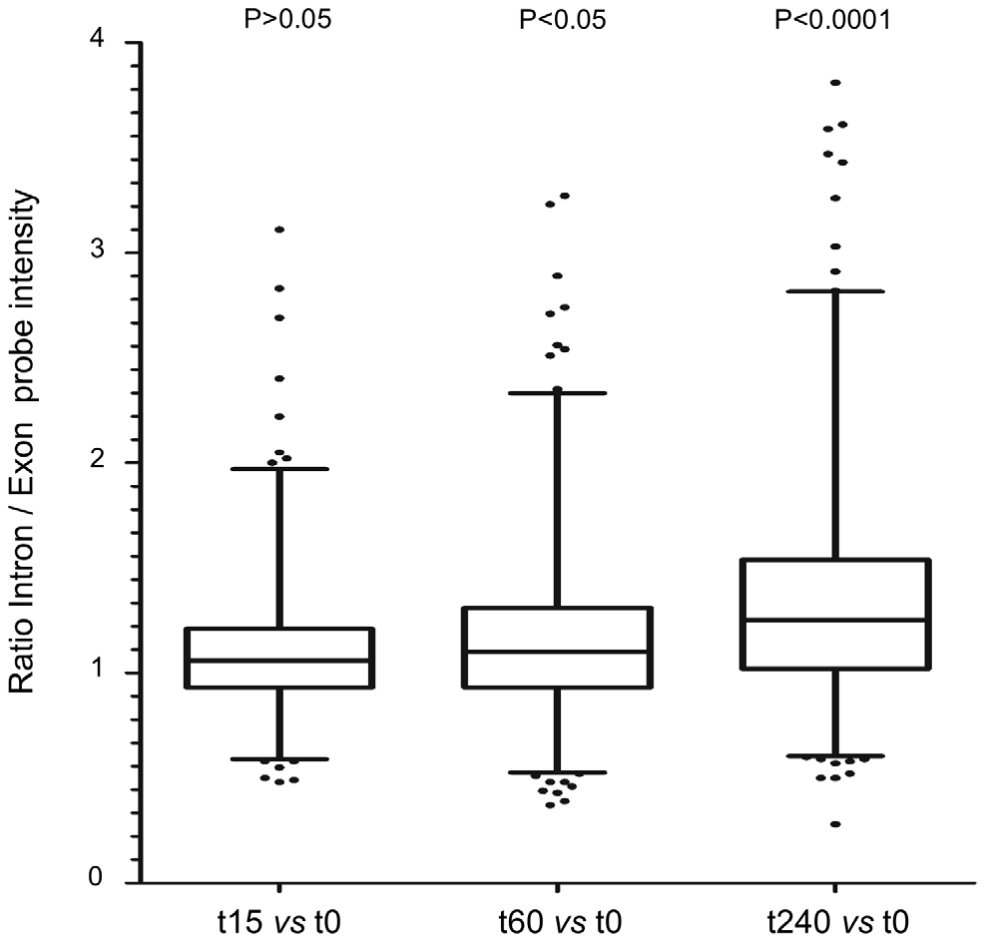
Box and whisker plots showing the ratio of intron/exon hybridization signal intensities after 5FU exposure. The analysis considered 948 intron-containing transcripts of *S. pombe*. The ratio represents the pre-mRNA/(mRNA+pre-mRNA) levels. The Individual boxes represent the median (central horizontal line) and the 75–25% percentiles. The whiskers extend from the boxes to 1% and 99% of the data set. Dots indicate outliers. Data are representative of two independent experiments. The P values between groups were calculated using the two-tailed Mann-Whitney test. The values at 240 min for introns *SPCC24B10.17_b* (5.2) and *SPAC1486.01_a* (4.9) are not shown.

The fission yeast nuclear genome consists of 5,175 annotated protein-coding genes, of which 2,404 are known or predicted to contain at least one intron with an average length of 81 nucleotides [20]. To obtain a direct measurement of intron-containing transcripts we calculated the average probe intensity across the 948 intronic regions that were delimited by, at least, 4 core probes in the microarray platform, since those data were deemed to be statistically significant (Table S3). Cells treated with 5FU showed a time-dependent global increase of unspliced transcripts, whereas the exon levels (representing a measure of both mature mRNA and pre-mRNA levels) did not significantly change, ruling out the possibility that these alterations were an indirect consequence of increments in the transcription rates (Figure S2). Consistent with this result is the fact that the levels of intronic regions relative to their corresponding flanking exons (intron/exon ratio) were significantly higher at 60 and 240 min after drug exposure than the untreated control (P<0.05 and P<0.0001, respectively) (Figure 2). In particular, more than 1.5-fold increase was detected for 12% of the genes at 60 min and 27.7% at 240 min of 5FU treatment (Table S3).

**Figure 3:**
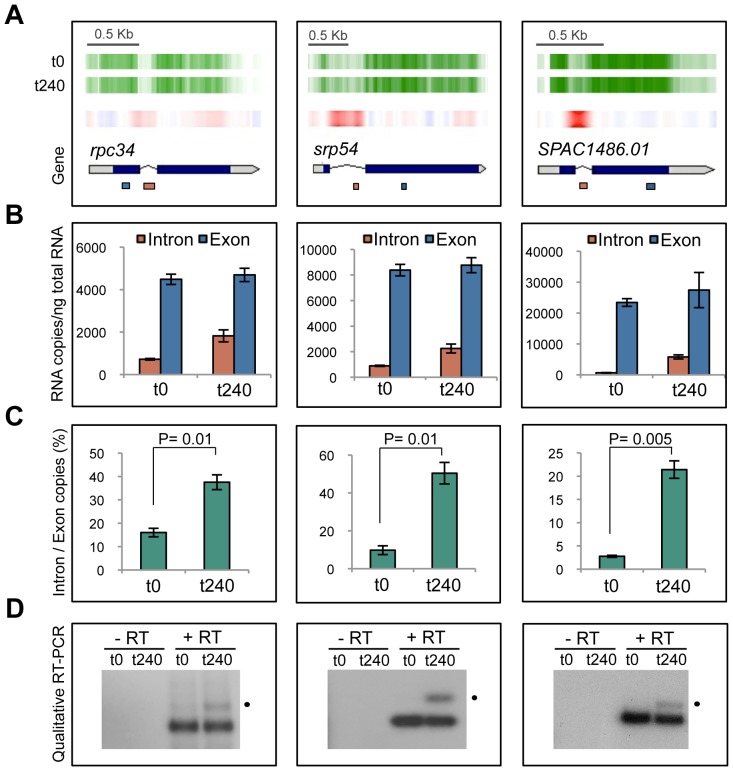
Validation of selected genes *rpc34*, *srp54* and *SPAC1486.01* for intron retention after 5FU exposure. (**A**) Visualization of microarray expression data. Vertical green lines represent transcription from sense DNA strand in *S. pombe* cells treated with 5FU for 240 min (t240) or untreated control (t0). Red vertical lines indicate a differential over-expression level between both conditions. Genes are shown as bars pointing towards the direction of transcription, grey sectors inside correspond to 5′- and 3′-untranslated regions and thin lines to introns. Boxes below indicate the amplified exon (blue) or intron (orange) fragments showed in “**B**”. (**B**) Absolute levels of intronic and exonic regions quantified by qPCR for each gene transcript in untreated or 5FU-treated cells. The values represent the RNA copy number per defined ng of total RNA. Bar charts show the average values ± s.d. of two independent experiments. (**C**) Proportion of intron/exon levels (measured by the normalized ratio of intron to exon copies). The P values between groups were calculated using the two-tailed Student’s t test. (**D**) Gel electrophoresis of RT-PCR products showing prominent bands corresponding to spliced mRNA at 0 and 240 min of 5FU exposure for *rpc34* (0.9 Kb), *srp54* (1.6 Kb) and *SPAC1486.01* (0.7 Kb) and upper bands with the expected size of the unspliced mRNA precursors for *rpc34* (1.1 Kb), *srp54* (2 Kb) and *SPAC1486.01* (0.8 Kb) that only appear after drug treatment. Reverse transcriptase is omitted from the reaction for the negative control (−RT).

To validate these results, we selected three genes (*rpc34*, *srp54* and *SPAC1486.01*) whose transcripts exhibited an increase in intron retention after 240 min of 5FU treatment, according to tiling microarray analysis (Table S3 and Figure 3A). Reverse transcription followed by qPCR experiments that amplified specific intron or exon segments, revealed that the percentage of intron-containing transcripts detected were between ∼2 and 8-fold higher than the untreated control (Figure 3B, 3C). The analysis of qualitative reverse transcriptase-PCR products on agarose gel electrophoresis further confirmed the presence of these unspliced transcripts (Figure 3D).

Taken together, our findings provide a novel global insight into pre-mRNA splicing defects that previously were only reported for a limited number of transcripts when exposed to 5FU [9], [21], [22]. The direct misincorporation of the 5FU metabolite FUTP into the mRNA precursors could possibly contribute to these alterations by preventing efficient splice site recognition and/or cleavage. However, several studies have shown that the splicing machinery efficiently processed 5FU-substituted pre-mRNA [9], [23]. This apparent contradiction could be explained if the incorporation of 5FU into the RNA components of the splicing machinery were the main cause of the processing defects reported rather than the 5FU-substitutions into substrate RNA. This hypothesis is supported by the fact that the incorporation of 5FU into the small nuclear RNAs (snRNA), which are key components of the spliceosome, caused impairment of pre-mRNA splicing [9], [24].

### Effects of 5FU on Expression and Splicing of tRNA

**Figure 4:**
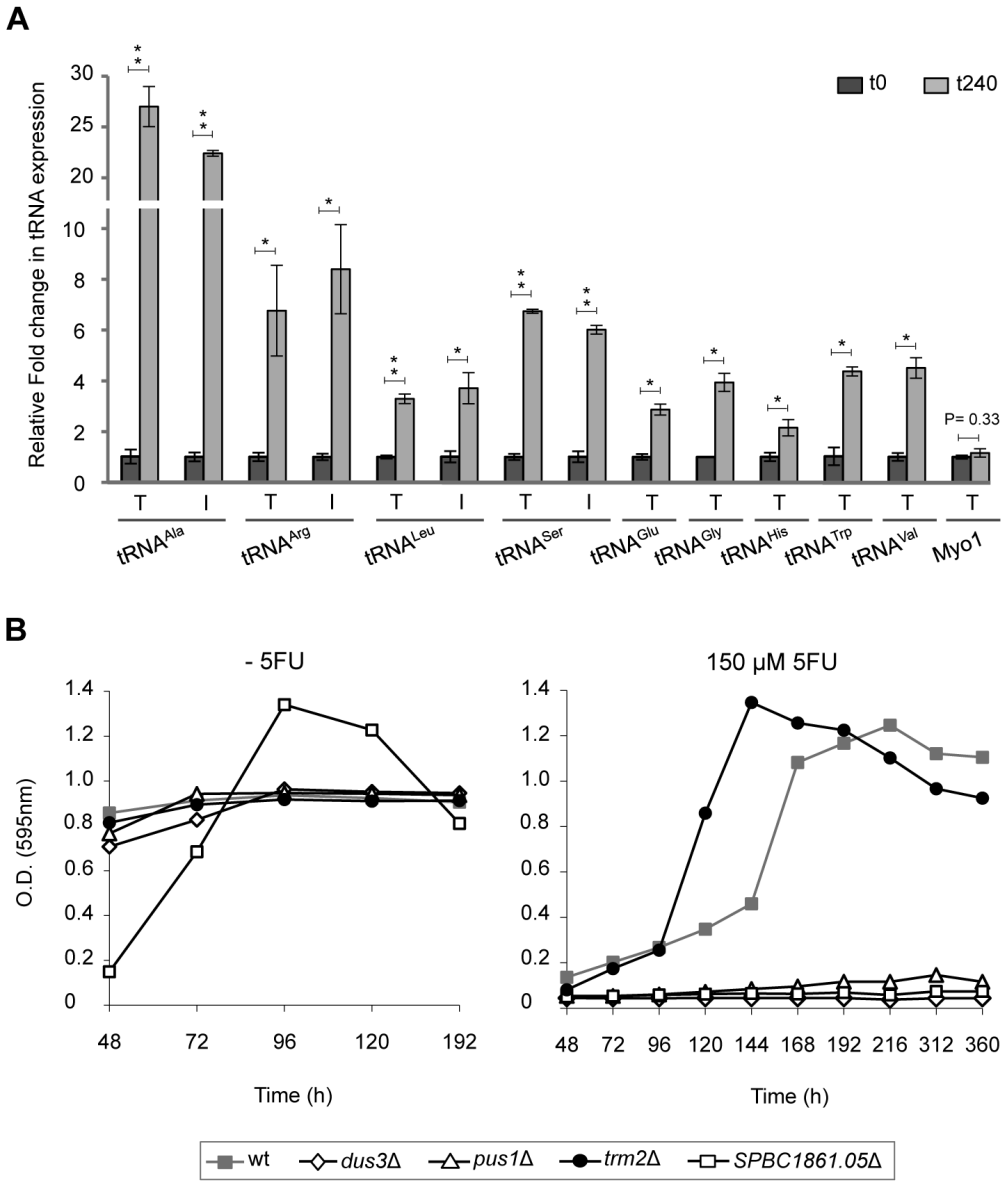
Effect of 5FU in tRNA transcripts and in the viability of different tRNA modification mutants. (**A**) Relative levels of intronic or exonic regions within transcripts of 9 selected tRNA genes at both 0 and 240 min of 5FU exposure quantified by qPCR. Genes for tRNA^Ala^_CGC_, tRNA^Arg^_CCU_ and tRNA^Gly^_CCC_ only have one copy in the genome, whereas multiple identical copies are found for the other tRNA genes examined. In these cases, the amplified products detected represent a mixture of the expression levels of the individual repetitions. The values represent the relative fold change in transcripts abundance between untreated and 5FU-treated cells normalized to the initial amount of starting RNA. Note that only differences between the same group are directly comparable. The expression levels of the normalization gene *myo1* were shown as a control. Bar charts show the average values ± s.d. of two independent experiments. Asterisks designate overall significance, * P<0.05, ** P<0.005, using the two-tailed Student’s t test. T represents the amount of total tRNA (combining both spliced and unspliced transcripts), whereas I represents the intron levels. (**B**) Sensitivity to 5FU of *S. pombe* strains deleted for genes involved in the modification of residues within tRNA molecules. The growth of the 5FU sensitive strain *dus3Δ* is also shown. Note that the control wild-type is the ED668 strain. The growth curve is representative of at least two independent experiments.

Intron-containing transcripts from protein-coding genes are not the only type of RNA molecules that undergo splicing in eukaryotic cells. A number of tRNA genes also contain introns that must be removed by an enzymatic cut-and-rejoin reaction that requires the heterotetrameric tRNA splicing endonuclease (SEN) complex to generate the mature and functional structure. Using the tRNAscan-SE program (Genomic tRNA Database, http://gtrnadb.ucsc.edu) a total of 186 tRNA genes were identified in *S. pombe*, 44 of which have introns (24% of the total) ranging in size from 7 to 30 nucleotides [25]. To test whether the pre-tRNA splicing was affected by 5FU, we compared the levels of unspliced transcripts after 240 min of drug exposure respect to the untreated control for four selected intron-containing tRNA genes that code for tRNA^Ala^_CGC_, tRNA^Arg^_CCU_, tRNA^Leu^_CAA_ and tRNA^Ser^_GCU_ (Figure 4A). Specific intronic or exonic regions within each tRNA were amplified using reverse transcription and qPCR experiments. A comparison of the intron to exon ratios at 0 and 240 min of 5FU treatment for each type of tRNA transcript revealed that only tRNA^Arg^_CCU_ showed a statistically significant increase in intron retention (1.2-fold higher, P = 0.05). The results also indicated that cell exposure to 5FU for 240 min had an enhanced expression of the selected intron-containing tRNA genes (Figure 4A). This finding led us to ask whether the levels of non intron-containing tRNA were also affected. The analysis showed that the amount of transcripts detected for tRNA^His^_GUG_, tRNA^Trp^_CCA_, tRNA^Gly^_CCC_, tRNA^Val^_UAC_ and tRNA^Glu^_CUC_ were notably higher after 5FU exposure, although their levels were lower than those in most of the intron-containing tRNA genes examined (Figure 4A).

To our knowledge, this is the first time that an increase in the tRNA levels has been observed in cells treated with 5FU. Previous studies reported that the 5FU-containing tRNA is able to form stable complexes with the tRNA pseudouridine synthase [26] and the tRNA 5-methyluridine-methyltransferase [27] causing the inhibition of both enzymatic activities. The subsequent sequestration of tRNA molecules and the presumable decrease in levels of pseudouridine and 5-methyluridine modifications, which stabilize specific structural tRNA motifs, may induce the expression of the tRNA genes. Accordingly, strains lacking these genes might have an increased sensitivity to 5FU. To test this, we analysed the effects of 5FU in *S. pombe* strains deleted for *pus1*, which encodes the only enzyme with demonstrated tRNA pseudouridine synthase activity [28], and *trm2*, a gene predicted to encode the only tRNA (m5U54) methyltransferase (Figure 4B). A strain deleted for *dus3*, the ortholog of the *Saccharomyces cerevisiae* tRNA dihydrouridine synthase 3 whose deletion increased the sensitivity to 5FU [29], was used as a control. The unexpected resistance of the *trm2*Δ cells (Figure 4B) could be explained if the protein plays a role in DNA repair, like its ortholog TRM2 in *S. cerevisiae* does, as this activity has the potential to promote cell cycle arrest in response to 5FU treatment, as previously shown for other enzymes involved in DNA repair pathways [30]. In contrast, the *pus1*Δ strain was highly sensitive to 5FU compared to the wild-type one (Figure 4B). These findings support previous research, which suggested that pseudouridine modifications are relevant targets for 5FU [29]. Based on these results, it would be expected that the inhibition of the pseudouridine metabolizing enzymes could also contribute to the cytotoxicity of 5FU. To test this hypothesis we examined the drug sensitivity of a strain disrupted for *SPBC1861.05*, a gene predicted to encode the only fission yeast enzyme able to modify (by phosphorylation) pseudouridine residues. The viability assays showed that this modification could be also one of the targets of 5FU, as the strain *SPBC1861.05*Δ was hypersensitive to the drug (Figure 4B).

### Defective Pre-rRNA Processing Caused by 5FU

**Figure 5:**
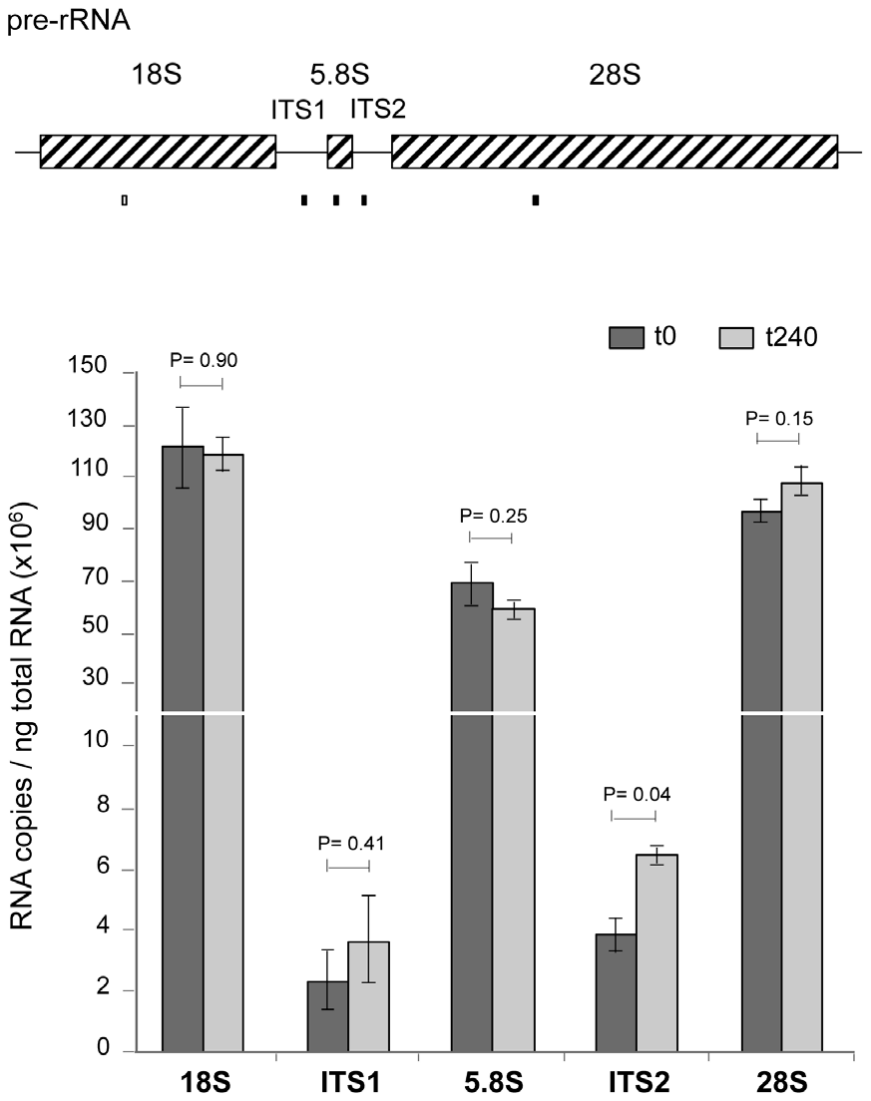
5FU induces the accumulation of unprocessed rRNA molecules. Absolute quantification of transcript levels of 5FU-treated cells during 240 min (t240) compared to control (t0) in several regions within the rRNA precursor. Boxes below the diagram show the specific segments amplified by qPCR. Bar charts show the average values ± s.d. of two independent experiments. The P values were calculated using the two-tailed Student’s t test.

Several studies have shown the capacity of 5FU to inhibit the processing of pre-rRNA into mature rRNA [7], [31], [32], the most abundant type of RNA in eukaryotic cells. In *S. pombe* the major rRNAs are transcribed from the 100–120 tandem repeats of the rDNA locus into a single precursor that contains the sequences for the mature 18S, 5.8S, and 28S rRNA separated by two internal transcribed spacers, ITS1 and ITS2 (Figure 5) [33]. The nuclear exosome, a highly conserved RNA processing complex, plays a critical role in the series of cleavage events required to generate mature rRNA molecules [34]. A genome-wide screen to assess the cellular effects of several chemical compounds in *S. cerevisiae* revealed that 5FU produces the accumulation of an rRNA processing product that includes a part of the ITS1 and ITS2 segments [32]. To further investigate whether 5FU causes a similar impairment in fission yeast, we compared the amount of transcripts detected by qPCR from several regions of the rRNA transcript after 240 min of drug treatment to the amount found in untreated cells (Figure 5). The expression levels for the 18S, ITS1, 5.8S, and 28S regions did not show any statistically significant differences; however, a significant 1.65-fold increase (P<0.05) was observed for ITS2 suggesting a defective pre-rRNA processing associated with the 5FU treatment in *S. pombe*.

### 5FU Induces the Transcription of Genes Involved in RNA Processing Pathways

Next we explored whether the observed RNA processing defects produced by 5FU in *S. pombe* caused a transcriptional activation response of the genes involved in such a pathway. To test this prediction, the expression levels of the genes involved in the processing of mRNA (180), tRNA (103) and rRNA (202) were evaluated at 0, 15, 60 or 240 min after drug exposure and the significantly up-regulated ones (greater than 1.5-fold, P<0.05) were identified (Table 1). RNA processing genes that were known to respond to multiple types of environmental stresses [35] were not included in order to only evaluate the specific response to 5FU.

**Table 1:** 5FU induced RNA processing genes in *S. pombe*.

Gene Name	Annotation^a^	Humanortholog^b^	t15 *vs*t0 FC P		t60 *vs*t0 FC P		t240 *vs*t0 FC P	
**mRNA processing**								
*SPAP8A3.06*	Splicing factor U2AF 23 kDa subunit	U2AF1	2.5	0.01	1.8	0.03		
*hub1*	Ubiquitin-like modifier hub1	HUB1			1.7	0.01	1.8	0.01
*ini1*	Pre-mRNA-splicing factor ini1	PHF5A			1.5	0.02	1.6	0.03
*prp38*	Pre-mRNA-splicing factor 38	PRPF38A	1.7	0.03				
*snu66*	U4/U6.U5 tri-snRNP-associated protein snu66	SART1			2.0	0.01		
*prp11*	Pre-mRNA-processing ATP-dependent RNA helicase	DDX46			1.7	<0.01		
*SPBC713.05*	Uncharacterized WD repeat-containing protein C713.05	WDR83			1.7	0.03		
*sap49*	Spliceosome-associated protein 49	SF3B4			1.5	<0.01		
*aar2*	A1 cistron-splicing factor aar2	AAR2			1.5	0.05	1.5	0.03
**tRNA processing**								
*tum1*	Putative 3-mercaptopyruvate sulfurtransferase	TST, MPST			1.9	0.02		
*SPAC10F6.04*	RCC1 repeat-containing protein C10F6.04	SERGEF			1.8	<0.01		
*sen54*	Probable tRNA-splicing endonuclease subunit sen54	TSEN54			1.6	<0.01		
*SPAC12B10.08c*	Probable tRNA(Ile)-lysidine synthase				1.6	<0.01		
*mss1*	tRNA modification GTPase mss1, mitochondrial	GTPBP3			1.6	0.02		
*ctu1*	Cytoplasmic tRNA 2-thiolation protein 1	CTU1			1.5	0.01	1.9	<0.01
*ctu2*	Cytoplasmic tRNA 2-thiolation protein 2	CTU2					1.6	0.02
**rRNA processing**								
*esf2*	Pre-rRNA-processing protein esf2	ABT1	1.6	<0.01	2.0	<0.01	1.7	<0.01
*cgr1*	rRNA-processing protein cgr1		1.6	0.01	1.6	<0.01	1.8	<0.01
*SPAC227.02c*	Ribosomal RNA-processing protein 15	RRP15	1.6	0.04	1.5	0.02		
*mrm2*	rRNA (uridine-2'-O-)-methyltransferase	FTSJ2			1.5	0.04		
*SPCC757.08*	Exosome complex component rrp45	EXOSC9					1.6	<0.01

**Footnotes:**

a Annotation provides a schematically description of the gene product as indicated in UniprotKB database (http://www.uniprot.org/). Genes were classified according to their function in Gene Ontology (GO) groups using PomBase Database (http://www.pombase.org/spombe/query/builder).

b Potential human orthologue genes or functional counterparts found in Homologene (http://www.ncbi.nlm.nih.gov/homologene/) and KOGs classification (http://www. ncbi.nlm.nih.gov/COG/grace/kognitor.html).

Abbreviation: FC, Fold change in gene expression after 15 min, 60 min or 240 min of 5FU treatment. Only values higher than the threshold (1.5) were shown. P values (P) were calculated as described in Materials and Methods.

One of the most interesting up-regulated genes is *snu66*, which, like gene *prp38*, encodes a subunit of the U4/U6.U5 pre-mRNA splicing small nuclear ribonucleoprotein (snRNP) complex. The human homologue of yeast Snu66, called SART1, is essential for the recruitment of the tri-snRNP to the pre-spliceosome [36]. Microarray expression profilings of metastatic colorectal cancer patient biopsies have recently revealed that SART1 transcription was up-regulated following 5FU treatment [37]. In addition, silencing of SART1 with siRNA (small interfering RNA) synergistically enhanced the apoptotic effects of 5FU in several colorectal cancer cell lines.

Another intriguing finding was the up-regulation of *hub1*, a gene encoding a Snu66 binding protein [38], which has been implicated in enabling the spliceosome to tolerate and to use some non-canonical 5′ splice sites. Relaxing the specificity of the spliceosome would be one of the cellular mechanisms to bypass the deleterious effect produced by the direct incorporation of 5FU into mRNA transcripts, or even into the small nuclear RNAs (snRNA) that are necessary for an efficient intron removal process. We also found that *aar2*, a gene required for the correct assembly of this spliceosomal tri-snRNP, was induced.

Moreover, *SPAP8A3.06* [39], *ini1* [40] and *sap49* [41] gene products are components of the U2 snRNP complex that binds to the intronic region at the branch site to generate a prespliceosome that is partially stabilized by Prp11 [42]. *SPBC713.05* encodes a protein purified as part of the spliceosome [43].

5FU also affects the expression of genes potentially involved in several post-transcriptional modifications of the tRNA wobble uridine, such as thiolation (*tum1*, *ctu1* and *ctu2*) and the formation of 5-methoxycarbonylmethyl (*SPAC10F6.04*) [44], [45] and 5-carboxymethylaminomethyl (*mss1*) groups [46]. It has been suggested that the absence of these modifications may influence decoding. *SPAC12B10.08c* is predicted to ligate lysine onto the cytidine present at the wobble position 34 of the tRNA^Ile^_AUA_, thus changing the amino acid specificity from methionine to isoleucine in the mitochondria [47]. On the other hand, the induction of *sen54* expression, a gene with a potential role in tRNA precursors maturation, suggests that 5FU could alter the splicing of some tRNA introns, as in the case of tRNA^Arg^_CCU_ transcripts (Figure 4A). Furthermore, it might be possible that some human tRNA genes show splicing defects after 5FU treatment, since the mechanism of tRNA splicing has been conserved through evolution [48].

The induction of various rRNA metabolic genes, such as *cgr1*, predicted to be required in the processing of the pre-rRNA for the 60S ribosome subunit [49], *esf2*, implicated in pre-18S rRNA processing [50] and *SPCC757.08* (*rrp45*), involved in the generation of mature 5.8S rRNA transcripts could be related to the defects found in rRNA maturation steps (Figure 5). In fact, Rrp45 is a component of the nuclear exosome complex that has been previously shown to be one of the main cellular targets for 5FU in *S. cerevisiae* [32]. *Mrm2*, a gene encoding a methyltransferase required for 2′-O-ribose methylation of the uracil at position 2,791 of yeast mitochondrial 21S rRNA [51] was also induced, suggesting the possibility that the catalytic activity of this enzyme might be impaired if 5FU arises at that position instead of uracil.

It is interesting to note that many of the up-regulated RNA processing genes are implicated in uridine modifications that substantially decrease after 5FU treatment, indicating the high specificity of the cellular response to the drug.

### Sensitivity to 5FU of Mutants Defective in RNA Processing Genes

In previous studies, the analysis of the transcriptional changes associated with 5FU exposure mainly focused on the comparison between 5FU sensitive and resistant tumour cell lines in order to identify biomarkers for predicting the efficacy of chemotherapeutic response [52]–[54]. However, gene expression profiles of cancer cells that were exposed to drug agents have been shown to correlate with drug sensitivity in several studies [55], [56]. Accordingly, the up-regulation of the RNA processing genes shown in Table 1 might reflect a cellular attempt to counteract the damaging effects of 5FU, and thus the deletion of some of these genes could enhance 5FU-induced apoptosis. To check this possibility, we selected *S. pombe* haploid mutants deleted for individual genes involved in RNA processing (Table 1) that are not essential for yeast viability.

**Figure 6:**
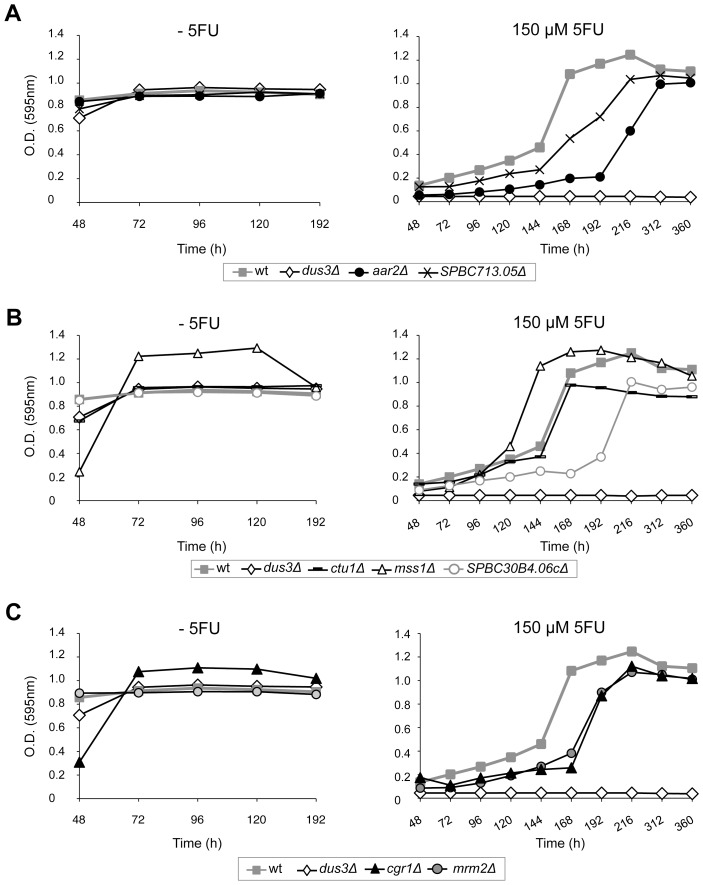
Sensitivity to 5FU of different mutants affected in RNA processing pathways. Strains deleted for genes involved in the processing of mRNA (**A**), tRNA (**B**) or rRNA (**C**) were tested for 5FU sensitivity. Mutants grew at near wild-type rates on liquid medium but exhibited reduced growth rates when 5FU was added to the cultures with the exception of the strain *mss1Δ*. The growth of the 5FU sensitive strain *dus3Δ* and the control wild-type (ED668) is also shown. Data are representative of three independent experiments.

The growth curve analysis revealed that strains *aar2*Δ, *cgr1*Δ, *ctu1*Δ, *mrm2*Δ and *SPBC713.05*Δ were more sensitive to 5FU than the wild-type control (Figure 6). However, the strain *mss1*Δ did not exhibit hypersensitivity to 5FU (Figure 6B), suggesting a modest contribution of this gene to the 5FU response or functional redundancy with other RNA processing genes. In this regard, the absence of *SPBC30B4.06c*, a protein required for Mss1 to modify uridine bases in mitochondrial tRNA, caused a reduction in the growth rates when 5FU was added to the cultures (Figure 6B). In a recent analysis, heterozygous deletion *S. pombe* strains were tested for sensitivity to several drugs, including 5FU [57]. Interestingly, *sen54*Δ and *tum1*Δ mutants showed an increased sensitivity to 5FU as predicted from our results.

Regardless of the specific mechanism, it seems clear that RNA processing pathways contribute to some extent to 5FU toxicity since mutants in a number of RNA processing proteins exhibit increased sensitivity to the drug. This result suggests that a combined treatment of 5FU with a new generation of antitumor drugs that have the spliceosome as its main target (spliceostatin, pladienolide) [58] could improve the efficacy of the drug. The splicing defects could directly activate apoptosis as might occur in the case of some traditional anticancer agents like cisplatin, which is capable of affecting the splicing of apoptotic genes [59].

Plenty of evidence suggests that RNA-based effects play a prominent role in 5FU cytotoxicity; however, the significance of the thymidylate synthase inhibition has also been largely proved. Multiple clinical studies have shown that a low tumoral thymidylate synthase expression is a predictive marker for a high response to 5FU-based chemotherapy [60], [61]. Alternatively, the down-regulation of the enzyme transcription in cancer cell lines considerably enhanced 5FU efficacy [62]. We checked that the deletion of the human thymidylate synthase ortholog in *S. pombe* (*SPAC15E1.04*) also improved the drug sensitivity (Figure S3), which provides additional evidence for validating the new candidates we propose as 5FU targets.

In conclusion, the analysis of the global transcriptional changes associated with 5FU exposure revealed a range of processing defects in RNA precursors as well as an increase in tRNA levels and an induction of several RNA processing genes. The deletion of many of these genes conferred sensitivity to 5FU showing that the transcriptional response can be used to predict potential drug targets. Further studies will be needed to determine whether lowering the levels of the corresponding human orthologs could increase the cellular sensitivity to 5FU treatment.

## Supporting Information

Figure S1 **Validation of microarray data using qPCR analysis.** We randomly selected two RNA processing genes (*ctu1* and *SPBC713.05*) and two genes (*ssa1* and *psi1*) whose orthologs in humans were induced after 5FU treatment. The expression levels obtained by qPCR (A) or microarray experiments (B) were compared after 15, 60 and 240 min of drug exposure with respect to untreated cells. For normalization, the expression level of the commonly used qPCR normalisation gene myo1 was set to 1. Bars represent average data for two independent biological replicates with each of the individual data points displayed by a cross.
(PDF)

Figure S2 Box and whisker plots showing the average of the probe intensities (Log2 scale) obtained by microarray experiments for the intronic (A) or exonic (B) regions of 948 intron-containing transcripts after exposure *S. pombe* cells for 0, 15, 60 and 240 min to 5FU. Individual boxes represent the median (central horizontal line) and the 75–25% percentiles. The whiskers extend from the boxes to minimum and maximum values. The data shown are representative of two independent experiments. The indicated P values between groups were calculated using the two-tailed Mann-Whitney test. No statistically significant differences were found in the signal intensity of exonic regions among different times.
(PDF)

Figure S3 **Sensitivity to 5FU of the** ***S. pombe*** **strain deleted for the thymidylate synthase gene.** The yeast strain deleted for *SPAC15E1.04* (the predicted ortholog of the human thymidylate synthase gene) was hypersensitive to 5FU. The growth of the 5FU sensitive strain *dus3Δ* and the control wild-type (ED668) is also shown. Data are representative of three independent experiments.
(PDF)

Table S1 Genotype of *S. pombe* strains used in this study.
(PDF)

Table S2 List of primers used in this study.
(PDF)

Table S3 Expression levels of intronic and exonic regions for 948 intron-containing transcripts detected by microarray analysis. A probe-filtering protocol was employed to process the hybridization signals in a quantitative manner to measure differential transcriptional expression as described in Materials and Methods. Genes were listed according to their systematic name. The subscript denotes the position within the gene of the intron that we examined (for example SPAC10F6.10_b correspond to the second intron of that particular ORF). Only introns delimited by a minimum of 4 core probes (nprobes) were analysed. Results for experiment 1 (t0_1, t15_1, t60_1 and t240_1) and 2 (t0_2, t15_2, t60_2 and t240_2) are indicated. P values were calculated using the two-tailed Student’s t test.
(PDF)
