# Supplementary material for: New Insights into the RNA-Based Mechanism of Action of the Anticancer Drug 5′-Fluorouracil in Eukaryotic Cells
Source: PLoS One. 2013 Nov 1;8(11):e78172. doi: 10.1371/journal.pone.0078172 (PMC3815194; doi:10.1371/journal.pone.0078172)

Supplementary Figure S1

A. Relative quantification of RNA with qPCR.

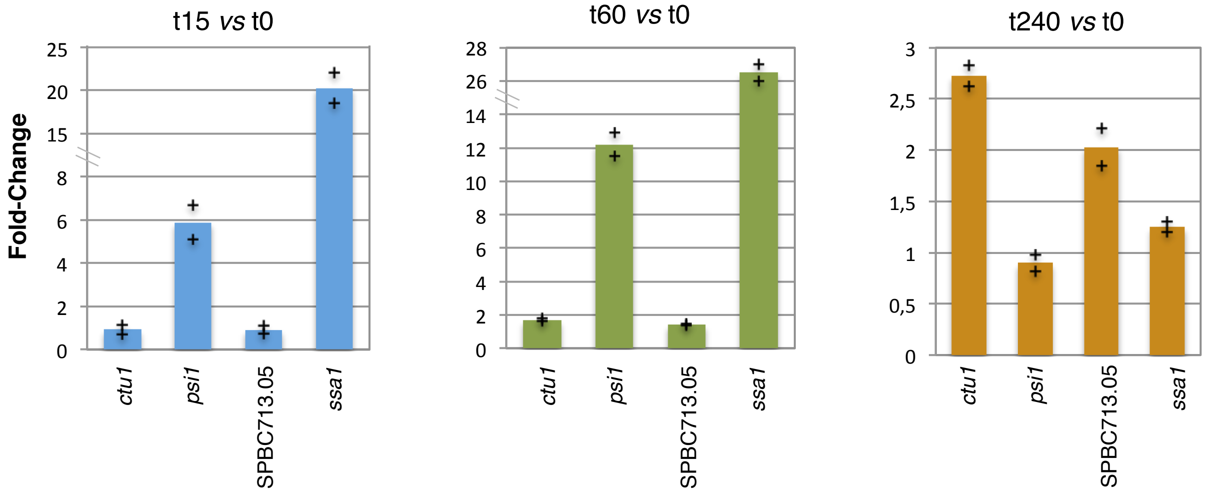

B. Relative quantification of RNA with microarray technology.

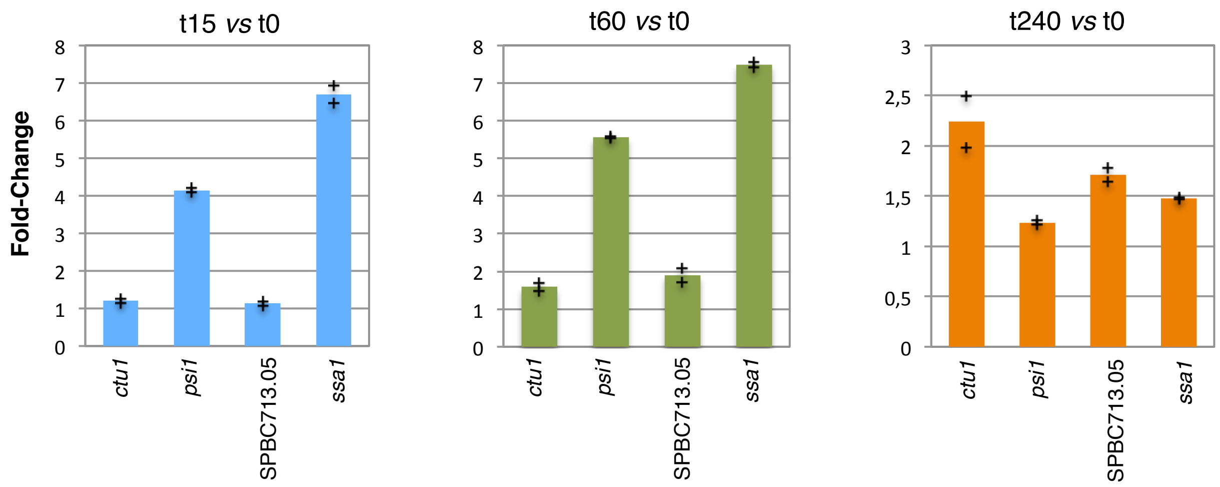

Supplement: Figure S1 — Validation of microarray data using qPCR analysis. We randomly selected two RNA processing genes (ctu1 and SPBC713.05) and two genes (ssa1 and psi1) whose orthologs in humans were induced after 5FU treatment. The expression levels obtained by qPCR (A) or microarray experiments (B) were compared after 15, 60 and 240 min of drug exposure with respect to untreated cells. For normalization, the expression level of the commonly used qPCR normalisation gene myo1 was set to 1. Bars represent average data for two independent biological replicates with each of the individual data points displayed by a cross. (PDF) [file pone.0078172.s001.pdf]
