# Supplementary figures and images for: New Insights into the RNA-Based Mechanism of Action of the Anticancer Drug 5′-Fluorouracil in Eukaryotic Cells
Source: PLoS One. 2013 Nov 1;8(11):e78172. doi: 10.1371/journal.pone.0078172 (PMC3815194; doi:10.1371/journal.pone.0078172)

Supplementary Figure S2

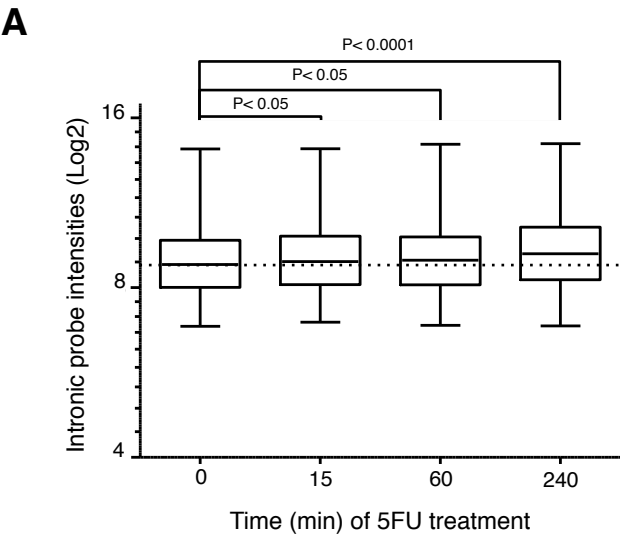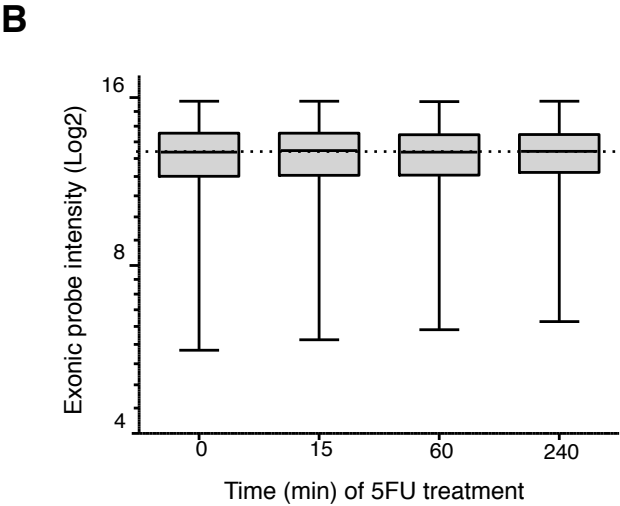

Supplement: Figure S2 — Box and whisker plots showing the average of the probe intensities (Log2 scale) obtained by microarray experiments for the intronic (A) or exonic (B) regions of 948 intron-containing transcripts after exposure S. pombe cells for 0, 15, 60 and 240 min to 5FU. Individual boxes represent the median (central horizontal line) and the 75–25% percentiles. The whiskers extend from the boxes to minimum and maximum values. The data shown are representative of two independent experiments. The indicated P values between groups were calculated using the two-tailed Mann-Whitney test. No statistically significant differences were found in the signal intensity of exonic regions among different times. (PDF) [file pone.0078172.s002.pdf]

Supplementary Figure S3

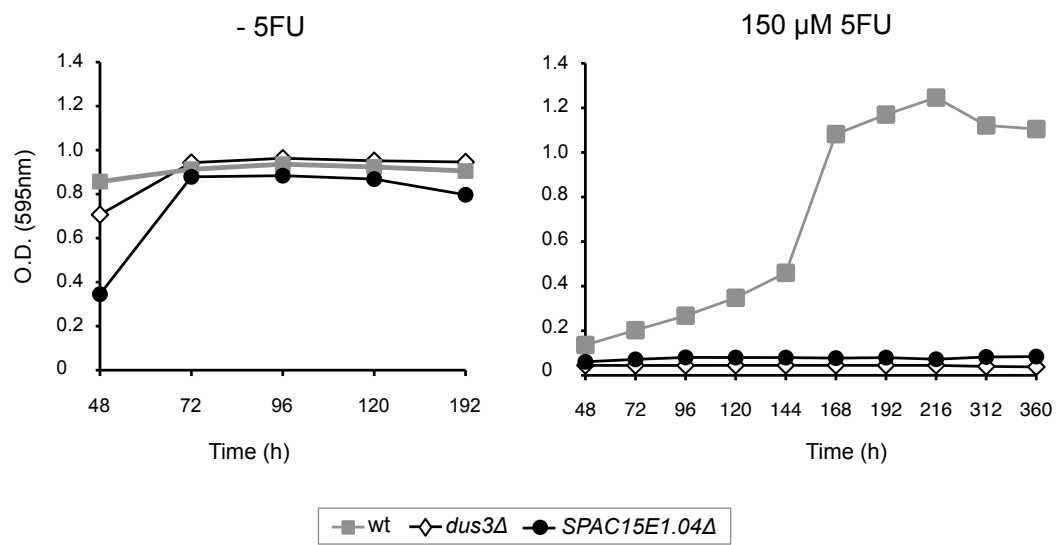

Supplement: Figure S3 — Sensitivity to 5FU of the S. pombe strain deleted for the thymidylate synthase gene. The yeast strain deleted for SPAC15E1.04 (the predicted ortholog of the human thymidylate synthase gene) was hypersensitive to 5FU. The growth of the 5FU sensitive strain dus3Δ and the control wild-type (ED668) is also shown. Data are representative of three independent experiments. (PDF) [file pone.0078172.s003.pdf]
