# Supplementary material for: New Insights into the RNA-Based Mechanism of Action of the Anticancer Drug 5′-Fluorouracil in Eukaryotic Cells
Source: PLoS One. 2013 Nov 1;8(11):e78172. doi: 10.1371/journal.pone.0078172 (PMC3815194; doi:10.1371/journal.pone.0078172)
Supplement: Table S1 — Genotype of S. pombe strains used in this study. (PDF) [file pone.0078172.s004.pdf]

Table S1. *Schizosaccharomyces pombe* strains used in this study

**Strain**	**Genotype**	**Source**
wt	*h- 972*	L. Blanco´s lab
ED668	*h+ ura4-D18 leu1-32 ade6-M216*	Bioneer
*aar2*Δ	*h+ aar2::KanMx4 ura4-D18 leu1-32 ade6-M216*	Bioneer
*cgr1*Δ	*h+ cgr1::KanMx4 ura4-D18 leu1-32 ade6-M216*	Bioneer
*ctu1*Δ	*h+ ctu1::KanMx4 ura4-D18 leu1-32 ade6-M216*	Bioneer
*dus3*Δ	*h+ dus3::kanMx4 ura4-D18 leu1-32 ade6-M216*	Bioneer
*mrm2*Δ	*h+ mrm2::KanMx4 ura4-D18 leu1-32 ade6-M216*	Bioneer
*mss1*Δ	*h+ mss1::KanMx4 ura4-D18 leu1-32 ade6-M216*	Bioneer
*pus1*Δ	*h+ pus1::kanMx4 ura4-D18 leu1-32 ade6-M216*	Bioneer
*SPAC15E1.04*Δ	*h+ SPAC15E1.04::kanMx4 ura4-D18 leu1-32 ade6-M216*	Bioneer
*SPBC1861.05*Δ	*h+ SPBC1861.05::kanMx4 ura4-D18 leu1-32 ade6-M216*	Bioneer
*SPBC30B4.06c*Δ	*h+ SPBC30B4.06c::kanMx4 ura4-D18 leu1-32 ade6-M216*	Bioneer
*SPBC713.05*Δ	*h+ SPBC713.05::KanMx4 ura4-D18 leu1-32 ade6-M216*	Bioneer
*trm2*Δ	*h+ trm2::KanMx4 ura4-D18 leu1-32 ade6-M216*	Bioneer
