# Supplementary material for: New Insights into the RNA-Based Mechanism of Action of the Anticancer Drug 5′-Fluorouracil in Eukaryotic Cells
Source: PLoS One. 2013 Nov 1;8(11):e78172. doi: 10.1371/journal.pone.0078172 (PMC3815194; doi:10.1371/journal.pone.0078172)
Supplement: Table S2 — List of primers used in this study. (PDF) [file pone.0078172.s005.pdf]

Table S2. List of primers used in this study

Oligos for qPCR to validate microarray expression data

Name	Sequence
Ctu1.F	5´ TGCGGAGTTTTTAGACGTCAG 3´
Ctu1.R	5´ TGTCCTGTAACCAAATGATGGA 3´
Psi1.F	5´ ACCTCATCCTGTTTTCACACG 3´
Psi1.R	5´ AAGGATAGCTCTACTTGCATACGG 3´
SPBC713_05.F	5´ AAGTACGTCCTTGCTGCTGGTAAC 3´
SPBC713_05.R	5´ CGCCGCTCTTTACATTCCAA 3´
Ssa1.F	5´ ATGGTATCTGAAGCCGAGAAGT 3´
Ssa1.R	5´ CTTGGCTTGAATACGAGAAGTTT 3´
Myo1.F	5´ CAAGCAACCACTGTTTCTGC 3´
Myo1.R	5´ GAAGAAGCTGCAGAAGTTACCG 3´

Oligos for qPCR to amplify mRNA (intron or exon)

Name	Sequence
**srp54**	
Primers for intron amplification	
I_srp54.F	5´ CCAAGCAACTTTTAGGCAGCTG 3´
I_srp54.R	5´ TCAAAACAGAACATTGGGAAAACAA 3´
Primers for exon amplification	
E_srp54.F	5´ CATCGTTGATACATCTGGTAGGC 3´
E_srp54.R	5´ TTCCACCATCTCAGCAAACA 3´
**rpc34**	
Primers for intron amplification	
I_rpc34.F	5´ ACTGTTTAAGCTTTTCGTTGTTGATC 3´
I_rpc34.R	5´ CATGATTAAAATTGGCGTAAAAACTTT 3´
Primers for exon amplification	
E_rpc34.F	5´ CTTCGATATCCGTGAACGATCTT 3´
E_rpc34.R	5´ TGCCGCAAAGGATCCAAA 3´
**SPAC1486.01**	
Primers for intron amplification	
I_SPAC1486.F	5´ AATTGCTCATGTTGATGGGTTCT 3´
I_SPAC1486.R	5´ ATTAACTAGACAGTGGGAAACATCCA 3´
Primers for exon amplification	
E_SPAC1486.F	5´ CAAAAGGAAGGTGGTGGCAA 3´
E_SPAC1486.R	5´ CCAAAGAACCCCATTTAGAGGTT 3´

Oligos for qPCR to amplify rRNA regions

Name	Sequence
18S.F	5´ GTTGTTGCAGTTAAAAAGCTCGTA 3´
18S.R	5´ ATGACCAGTAAACACGCCTTG 3´
ITS1.F	5´ AAATGAAATTGTAAATATTACGAGTGGATG 3´
ITS1.R	5´ TGATATGCTTGGCATGCAACA 3´
5.8S.F	5´ TCAGCAACGGATCTCTTGGC 3´
5.8S.R	5´ TGCAATTCACATTACGTATCGCAT 3´
ITS2.F	5´ AAATATTTTTGATGAGGTGTTGAACGAA 3´
ITS2.R	5´ TCTTCCTTTTGTTTTCACCAATCG 3´
28S.F	5´ GAGGTAAAGCGAATGATTAGAGGT 3´
28S.R	5´ TTAAAGTTTGAGAATAGGTTGAGGAAA 3´

Oligos for qPCR to amplify tRNA (intron or exon)

Name	Sequence
**tRNA-Arg (anticodon CCU)**	
Primers for intron amplification	
ARG.F	5´ GACCTGATCGTTGTTGGG 3´
ARG.R	5´ CTTACACGACGGGACTCG 3´
Primers for total amplification	
ARG.1	5´GCAATGGTAGCGCATCTC 3´
ARG.R	5´ CTTACACGACGGGACTCG 3´
**tRNA-Leu (anticodon CAA)**	
Primers for intron amplification	
LEU.F	5´ TTGGCCGAGCGGTCTAT 3´
LEU.R	5´ CAGCAAGACTATCGTCCAAGTAT 3´
Primers for total amplification	
LEU.F	5´ TTGGCCGAGCGGTCTAT 3´
LEU.2	5´ ATTCGAACCCTCGCATCT 3´
**tRNA-Ser (anticodon GCU)**	
Primers for intron amplification	
SER.F	5´ GCCTGCTATTCTGTAGCCC 3´
SER.R	5´ ACAACGGCAGGATTCGAA 3´
Primers for total amplification	
SER.1	5´ CCGAGTGGTTTTAAGGCGT 3´
SER.R	5´ ACAACGGCAGGATTCGAA 3´
**tRNA-Ala (anticodon CGC)**	
Primers for intron amplification	
ALA.F	5´ ATTCGCATAGTTCGCACC 3´
ALA.R	5´ AGATGCCGGGAATCGAA 3´

Primers for total amplification

ALA.1	5´ GGGGATGTAGTTTAGGGGTATAAC 3´
ALA.R	5´ AGATGCCGGGAATCGAA 3´

tRNA-His (anticodon GUG)

HIS.F	5´ GCTCACATGGTCCAGTGGTT 3´
HIS.R	5´ GCCCACACCAGGAATCGAA 3´

tRNA-Trp (anticodon CCA)

TRP.F	5´ GGCCCCTTAACTCAGTTGGT 3´
TRP.R	5´ TGACCCCTAAGTGACTTGAACA 3´

tRNA-Gly (anticodon CCC)

GLY.F	5´ CATTAGTGGTGTAGCGGTAACATTGG 3´
GLY.R	5´ TGCATCAGCCGGGAGTCG 3´

tRNA-Val (anticodon UAC)

VAL.F	5´ GCATCTGTAGTCTAGTGGTTATGA 3´
VAL.R	5´ GCATCTACCAGGGATCGAA 3´

tRNA-Glu (anticodon CUC)

GLU.F	5´ CCGTCATGGTCCAGTGGCTA 3´
GLU.R	5´ TCCGTCAGGGGGAATCGAA 3´

Control Myo1

Myo1.F	5´ CAAGCAACCACTGTTTCTGC 3´
Myo1.R	5´ GAAGAAGCTGCAGAAGTTACCG 3´
