# Supplementary material for: New Insights into the RNA-Based Mechanism of Action of the Anticancer Drug 5′-Fluorouracil in Eukaryotic Cells
Source: PLoS One. 2013 Nov 1;8(11):e78172. doi: 10.1371/journal.pone.0078172 (PMC3815194; doi:10.1371/journal.pone.0078172)
Supplement: Table S3 — Expression levels of intronic and exonic regions for 948 intron-containing transcripts detected by microarray analysis. A probe-filtering protocol was employed to process the hybridization signals in a quantitative manner to measure differential transcriptional expression as described in Materials and Methods. Genes were listed according to their systematic name. The subscript denotes the position within the gene of the intron that we examined (for example SPAC10F6.10_b correspond to the second intron of that particular ORF). Only introns delimited by a minimum of 4 core probes (nprobes) were analysed. Results for experiment 1 (t0_1, t15_1, t60_1 and t240_1) and 2 (t0_2, t15_2, t60_2 and t240_2) are indicated. P values were calculated using the two-tailed Student’s t test. (PDF) [file pone.0078172.s006.pdf]

Table S3. Expression levels of intronic and exonic regions for 948 intron-containing transcripts detected by microarray analysis.

A probe-filtering protocol was employed to process the hybridization signals in a quantitative manner to measure differential transcriptional expression as described in Materials and Methods.
Genes were listed according to their systematic name.
The subscript denotes the position within the gene of the intron that we examined (for example SPAC10F6.10_b correspond to the second intron of that particular ORF).
Only introns delimited by a minimum of 4 core probes (nprobes) were analysed. Results for experiment 1 (t0_1, t15_1, t60_1 and t240_1) and 2 (t0_2, t15_2, t60_2 and t240_2) are indicated.
P values were calculated using the two-tailed Student's t test.

Intron name	nprobes	I t0_1	I t0_2	I t15_1	I t15_2	P t15/t0 (I)	E t0_1	E t0_2	E t15_1	E t15_2	P t15/t0 (E)	I t60_1	I t60_2	P t60/t0 (I)	E t60_1	E t60_2	P 60/t0 (E)	I t240_1	I t240_2	P t240/t0 (I)	E t240_1	E t240_2	P t240/t0 (E)
SPAC1002.01_a	5	1184,45	734,19	1209,34	704,28	9,95E-01	6163,97	5268,70	6451,93	5055,41	9,68E-01	1226,22	797,86	8,81E-01	4788,85	4313,30	1,48E-01	2503,97	2091,03	4,84E-02	4976,86	5365,23	3,80E-01
SPAC1002.07c_e	9	504,95	487,75	861,08	704,28	6,82E-02	6700,25	6251,56	7434,40	6700,25	3,03E-01	666,29	709,18	1,43E-02	7912,95	8135,41	2,52E-02	670,92	639,15	1,27E-02	5293,48	4640,29	6,25E-02
SPAC1002.15c_c	6	388,02	263,20	508,46	315,17	5,32E-01	5634,22	4973,34	5256,91	4544,80	4,94E-01	467,88	401,71	2,62E-01	5914,33	5404,70	4,84E-01	770,69	568,10	1,02E-01	6295,04	5832,91	2,00E-01
SPAC1006.03c_b	8	4576,41	3769,09	4482,23	3848,29	9,90E-01	4752,64	4754,37	4882,17	4599,39	9,36E-01	4608,24	4329,55	5,60E-01	5290,20	5038,55	8,24E-02	6122,90	5914,33	4,74E-02	5629,23	5460,48	1,12E-02
SPAC1006.03c_c	12	3040,30	1795,29	3040,30	2048,00	8,88E-01	4752,64	4754,37	4882,17	4599,39	9,36E-01	2797,65	2062,24	9,88E-01	5290,20	5038,55	8,24E-02	4211,15	4011,71	1,15E-01	5629,23	5460,48	1,12E-02
SPAC105.01c_a	7	494,56	433,53	491,14	552,56	3,13E-01	3056,89	3455,51	2128,25	2434,55	6,05E-02	608,87	797,86	1,38E-01	3195,67	3393,95	8,78E-01	837,53	770,69	1,73E-02	3256,06	3649,68	5,55E-01
SPAC105.02c_a	13	639,15	564,18	873,10	826,00	3,04E-02	26615,89	27554,49	28133,47	26801,01	6,85E-01	776,05	648,07	2,75E-01	26987,43	27364,16	8,74E-01	1002,93	903,89	2,98E-02	27554,49	28133,47	3,03E-01
SPAC10F6.03c_a	8	202,25	215,27	188,71	210,84	5,56E-01	16794,76	15932,10	17423,57	16715,46	3,33E-01	183,55	167,73	8,38E-02	13667,71	13333,58	2,51E-02	187,40	181,02	7,72E-02	16924,98	16164,02	7,83E-01
SPAC10F6.10_b	4	224,41	242,19	256,00	233,94	4,97E-01	2399,88	2221,03	2803,05	2569,72	1,25E-01	284,05	259,57	1,26E-01	2823,33	2456,61	2,48E-01	310,83	296,11	2,60E-02	3054,75	2561,98	1,98E-01
SPAC110.03_a	6	347,29	319,57	564,18	436,55	1,25E-01	27554,49	24154,43	25355,30	24322,43	6,25E-01	455,09	290,02	6,86E-01	25531,66	23010,42	5,32E-01	533,74	512,00	8,53E-03	24661,96	23331,64	4,16E-01
SPAC110.03_b	18	814,63	537,45	843,36	657,11	7,00E-01	27556,94	24218,70	25308,54	24336,01	6,02E-01	792,35	592,22	9,33E-01	25501,30	23028,33	5,16E-01	903,89	657,11	6,30E-01	24586,37	23302,24	3,91E-01
SPAC1142.07c_a	5	194,01	183,55	249,00	194,01	3,63E-01	2175,93	1729,34	2472,19	2177,15	2,99E-01	221,32	249,00	8,85E-02	2660,70	2200,34	2,75E-01	259,57	268,73	8,40E-03	2554,23	1872,68	5,88E-01
SPAC11E3.04c_b	6	564,18	580,04	648,07	572,05	4,31E-01	25187,91	24287,26	23902,46	22358,41	2,14E-01	588,13	643,59	2,69E-01	22456,68	22287,61	3,55E-02	1351,18	1418,35	1,80E-03	22248,32	24431,21	3,58E-01
SPAC11E3.06_b	9	179,77	171,25	213,78	195,36	1,03E-01	1310,53	1037,39	1143,93	1133,32	8,20E-01	240,52	272,48	3,93E-02	2023,34	1993,79	2,60E-02	191,34	174,85	5,00E-01	1513,42	1233,14	4,16E-01
SPAC11E3.10_b	6	1296,13	916,51	1097,50	797,86	5,79E-01	13848,34	13253,29	13152,13	11480,98	2,99E-01	843,36	922,88	3,69E-01	10173,10	9706,30	1,08E-02	1031,12	1226,22	9,26E-01	11024,03	10736,95	1,50E-02
SPAC11G7.02_c	5	352,14	306,55	292,04	240,52	2,08E-01	11403,01	12687,87	11355,41	12339,59	8,30E-01	296,11	245,57	2,28E-01	10451,44	11012,17	2,02E-01	257,78	274,37	1,21E-01	11161,58	11673,77	4,60E-01
SPAC11H11.03c_b	15	445,72	415,87	421,68	369,65	3,62E-01	693,68	621,15	674,07	635,79	9,57E-01	580,04	530,06	5,07E-02	1092,92	854,56	1,26E-01	657,11	661,68	4,33E-03	1269,52	832,25	2,18E-01
SPAC1250.03_a	4	206,50	176,07	245,57	190,02	4,91E-01	13881,79	12001,82	13876,25	11716,16	9,28E-01	218,27	191,34	5,74E-01	14708,31	12258,71	7,59E-01	306,55	352,14	3,72E-02	13896,84	12805,78	7,43E-01
SPAC1250.05_a	9	233,94	247,28	218,27	243,88	5,77E-01	9490,85	8985,44	8244,48	8589,66	1,15E-01	216,77	242,19	5,19E-01	4548,35	3737,71	8,67E-03	207,94	168,90	1,27E-01	6522,81	4947,61	5,15E-02
SPAC1296.01c_d	4	699,41	442,64	504,95	504,95	6,58E-01	3536,22	3233,95	4189,66	4342,58	3,50E-02	451,94	526,39	6,03E-01	5137,57	5110,06	7,53E-03	424,61	494,56	4,90E-01	4628,35	4167,51	6,67E-02
SPAC1296.02_a	16	630,35	556,41	855,13	709,18	1,47E-01	17438,64	18561,17	22536,88	20452,65	9,81E-02	729,11	580,04	5,39E-01	19215,73	18053,61	5,14E-01	734,19	803,41	7,42E-02	20594,91	23821,89	1,33E-01

SPAC1296.04_b	11	962,07	792,35	1314,23	1200,98	6,50E-02	6936,54	6472,02	6700,25	6122,90	5,12E-01	1323,37	1296,13	3,73E-02	6653,97	6038,61	4,51E-01	1468,37	1640,59	3,04E-02	5873,48	5220,60	1,02E-01
SPAC1296.05c_a	4	235,57	256,00	238,86	212,31	3,51E-01	1019,27	915,62	990,10	933,48	9,32E-01	207,94	227,54	1,86E-01	978,27	791,53	5,20E-01	261,38	238,86	8,02E-01	1180,54	935,27	5,67E-01
SPAC12B10.09_b	6	221,32	191,34	254,23	213,78	3,86E-01	374,91	393,19	465,70	459,64	1,47E-02	259,57	199,47	5,61E-01	580,09	435,61	2,31E-01	254,23	270,60	8,15E-02	506,24	380,80	4,47E-01
SPAC12B10.16c_a	6	328,56	300,25	308,69	278,20	4,20E-01	6598,57	6554,79	6624,80	6523,11	9,65E-01	308,69	308,69	7,26E-01	6596,89	6145,36	4,60E-01	519,15	464,65	2,86E-02	5441,19	5659,04	1,15E-02
SPAC12G12.11c_b	4	143,01	151,17	195,36	157,59	2,68E-01	1492,10	1490,65	1548,96	1472,86	6,59E-01	256,00	124,50	5,80E-01	1428,53	1354,26	1,15E-01	176,07	216,77	1,41E-01	1585,50	1342,93	8,44E-01
SPAC139.06_c	4	168,90	176,07	233,94	190,02	2,18E-01	10734,74	9216,48	10513,82	8779,97	8,02E-01	235,57	202,25	1,12E-01	8719,32	7750,10	1,93E-01	333,14	257,78	8,31E-02	9089,59	8248,98	2,71E-01
SPAC1399.05c_a	4	282,09	187,40	284,05	227,54	7,39E-01	4526,10	6694,56	4006,18	5106,40	4,77E-01	218,27	207,94	6,94E-01	3519,46	4426,55	2,98E-01	282,09	286,03	4,07E-01	5801,39	7631,23	5,17E-01
SPAC13A11.04c_d	4	474,41	491,14	413,00	413,00	1,41E-02	3055,64	2786,03	2888,75	2624,43	4,76E-01	442,64	340,14	2,21E-01	3133,24	2713,92	9,92E-01	568,10	413,00	9,30E-01	3430,82	2471,43	9,57E-01
SPAC13F5.04c_a	12	910,17	617,37	820,30	552,56	7,34E-01	10485,28	9040,48	9563,17	7863,13	4,46E-01	739,29	477,71	5,12E-01	8500,88	7411,82	1,84E-01	694,58	515,56	4,53E-01	8850,86	7367,18	2,51E-01
SPAC13F5.04c_c	9	580,04	352,14	433,53	272,48	5,03E-01	10485,28	9040,48	9563,17	7863,13	4,46E-01	349,71	268,73	3,24E-01	8500,88	7411,82	1,84E-01	436,55	256,00	4,96E-01	8850,86	7367,18	2,51E-01
SPAC13F5.06c_c	10	292,04	261,38	280,14	256,00	7,01E-01	3030,49	3078,81	3279,26	3204,96	5,16E-02	278,20	266,87	8,22E-01	3628,71	3407,71	5,47E-02	300,25	284,05	4,67E-01	3522,42	3635,28	1,34E-02
SPAC13F5.07c_a	8	265,03	259,57	300,25	259,57	4,81E-01	254,19	282,83	218,66	307,38	9,17E-01	354,59	317,37	5,94E-02	352,84	315,92	1,06E-01	302,33	294,07	1,85E-02	319,68	242,56	7,88E-01
SPAC13G6.04_b	6	219,79	181,02	298,17	274,37	6,36E-02	2486,67	1820,35	3326,99	2435,50	3,21E-01	216,77	218,27	4,71E-01	2352,53	2385,37	5,85E-01	232,32	254,23	1,94E-01	3125,78	2957,17	1,23E-01
SPAC13G6.05c_c	10	2120,22	2149,82	1260,69	982,29	1,85E-02	12746,07	12584,03	11423,15	10981,42	2,49E-02	1584,71	1770,57	3,98E-02	10163,14	10610,09	1,07E-02	1758,34	2019,80	2,03E-01	10829,81	12164,23	2,24E-01
SPAC13G6.09_a	5	218,27	183,55	221,32	200,85	6,64E-01	7090,39	6620,21	8215,69	6850,93	4,47E-01	354,59	206,50	4,05E-01	7740,79	6785,06	5,24E-01	280,14	286,03	4,30E-02	7419,06	7554,19	1,23E-01
SPAC13G6.11c_a	10	564,18	537,45	560,28	398,93	4,76E-01	2419,18	2461,50	2786,71	2651,65	5,88E-02	484,38	474,41	3,76E-02	2184,40	2097,77	2,50E-02	694,58	675,59	1,46E-02	2127,62	1932,97	5,42E-02
SPAC13G7.08c_b	4	292,04	245,57	501,46	451,94	2,56E-02	10513,82	9741,98	11190,60	10369,08	3,67E-01	390,72	484,38	8,40E-02	9345,14	8480,89	1,71E-01	372,22	448,82	8,71E-02	10297,45	9741,98	8,41E-01
SPAC144.03_b	16	364,56	330,84	385,34	317,37	9,32E-01	21800,04	20914,45	20670,52	20692,80	2,67E-01	413,00	445,72	7,37E-02	17776,48	16987,29	2,15E-02	430,54	413,00	6,00E-02	17071,27	16958,32	1,04E-02
SPAC1486.01_a	4	885,29	809,00	1629,26	1428,22	2,40E-02	31651,80	31433,17	32768,00	32768,00	7,86E-03	2105,58	2352,53	8,63E-03	33923,56	36358,37	9,86E-02	4240,45	4067,71	8,14E-04	30786,28	31651,80	5,44E-01
SPAC1486.08_a	5	210,84	232,32	268,73	190,02	8,66E-01	1207,26	1153,82	1964,68	1820,55	1,15E-02	296,11	249,00	1,88E-01	2122,68	2240,86	4,17E-03	282,09	276,28	3,54E-02	1914,80	1981,31	3,08E-03
SPAC1486.09_a	9	526,39	519,15	568,10	491,14	8,76E-01	1439,80	1270,94	1521,87	1420,08	3,62E-01	604,67	584,07	2,25E-02	1485,05	1320,52	7,26E-01	596,34	608,87	8,12E-03	1934,41	1556,58	2,00E-01
SPAC14C4.11_a	9	377,41	313,00	385,34	315,17	9,25E-01	4163,57	3863,04	6232,65	5243,43	7,93E-02	349,71	354,59	8,50E-01	4463,96	3204,63	8,08E-01	448,82	461,44	7,88E-02	4546,56	4576,71	6,82E-02
SPAC14C4.14_a	6	1833,01	1243,34	1686,71	1488,87	8,88E-01	29628,36	29409,40	27882,53	28821,18	1,36E-01	1552,09	1488,87	9,58E-01	25861,99	27222,91	4,97E-02	2817,11	2385,37	1,01E-01	27570,88	27178,76	1,08E-02
SPAC14C4.14_b	12	1009,90	982,29	1105,13	1016,93	2,95E-01	29628,36	29409,40	27882,53	28821,18	1,36E-01	1105,13	1067,48	6,09E-02	25861,99	27222,91	4,97E-02	1820,35	1509,65	5,03E-02	27570,88	27178,76	1,08E-02
SPAC1527.02_b	5	729,11	617,37	891,44	617,37	6,38E-01	3745,41	3205,15	3953,40	3558,19	4,90E-01	709,18	568,10	7,38E-01	3687,06	3471,27	7,55E-01	1016,93	765,36	2,54E-01	3359,87	2965,30	4,49E-01
SPAC1556.02c_a	4	564,18	445,72	556,41	484,38	8,44E-01	24299,62	24570,41	27350,89	27544,81	3,04E-03	596,34	494,56	6,56E-01	27453,81	27621,59	2,62E-03	714,11	903,89	1,13E-01	28062,94	29746,08	3,45E-02
SPAC1556.07_a	9	526,39	458,25	421,68	390,72	1,48E-01	21622,65	17584,28	21695,13	20199,45	5,96E-01	393,44	357,05	9,38E-02	19425,79	17105,95	6,24E-01	613,11	512,00	3,68E-01	17811,35	14689,84	3,19E-01
SPAC1565.04c_a	7	296,11	254,23	357,05	229,13	8,15E-01	762,82	741,05	717,30	625,22	2,30E-01	292,04	265,03	9,05E-01	476,74	450,08	3,54E-03	359,54	342,51	7,85E-02	650,37	534,74	1,14E-01
SPAC1565.06c_c	5	1176,27	1045,52	1351,18	1128,35	4,24E-01	13630,91	12117,18	13238,73	12604,25	9,59E-01	1260,69	1160,07	3,51E-01	12430,05	12246,14	5,55E-01	1260,69	1296,13	1,32E-01	11336,32	11998,51	2,82E-01
SPAC15A10.05c_c	4	359,54	315,17	324,03	284,05	3,81E-01	7946,63	6243,55	5541,23	5853,31	2,48E-01	333,14	330,84	8,32E-01	3270,91	3504,50	4,98E-02	352,14	354,59	5,46E-01	6832,32	5305,79	4,64E-01

SPAC15A10.06_a	21	1251,98	1016,93	1858,60	1595,73	7,82E-02	9152,82	9026,81	11993,79	12416,75	4,98E-03	1200,98	989,12	8,27E-01	11425,74	11746,96	4,74E-03	1045,52	948,83	3,93E-01	9026,81	9280,58	6,96E-01
SPAC15A10.08_b	5	196,72	233,94	192,67	192,67	3,48E-01	2316,56	2375,48	2698,81	2506,51	1,25E-01	219,79	203,66	8,75E-01	3054,58	2701,16	9,72E-02	173,65	187,40	2,21E-01	2471,31	2262,51	8,65E-01
SPAC15A10.12c_a	6	458,25	294,07	418,77	436,55	5,96E-01	918,76	794,18	969,54	896,20	4,01E-01	504,95	481,04	2,94E-01	848,76	855,15	9,49E-01	749,61	680,29	6,27E-02	1038,45	941,68	2,32E-01
SPAC15E1.03_a	7	427,57	319,57	541,19	398,93	3,93E-01	16612,71	14766,09	16384,00	15393,14	8,67E-01	401,71	330,84	9,20E-01	13969,57	13034,07	1,69E-01	455,09	342,51	7,77E-01	15608,02	15286,81	8,20E-01
SPAC15E1.08_b	13	652,58	533,74	634,73	552,56	9,95E-01	13335,60	13304,99	13731,58	13921,83	3,43E-02	515,56	508,46	3,06E-01	11909,20	12205,74	1,36E-02	639,15	580,04	8,28E-01	12962,85	14388,26	6,68E-01
SPAC15E1.08_d	8	955,43	1038,29	867,07	661,68	1,71E-01	13335,60	13304,99	13731,58	13921,83	3,43E-02	719,08	694,58	2,15E-02	11909,20	12205,74	1,36E-02	1128,35	1438,15	2,16E-01	12962,85	14388,26	6,68E-01
SPAC15E1.09_a	11	374,81	278,20	359,54	330,84	7,46E-01	3521,84	3357,89	4467,31	4155,51	3,85E-02	352,14	340,14	7,26E-01	4188,74	3976,54	4,09E-02	354,59	418,77	4,08E-01	3854,71	3668,82	1,22E-01
SPAC16.01_b	5	326,29	310,83	364,56	282,09	9,20E-01	20106,06	20345,11	19950,92	20447,66	9,33E-01	335,46	321,80	4,32E-01	22045,43	21161,80	9,49E-02	439,59	357,05	1,98E-01	22501,82	22350,03	4,12E-03
SPAC16.02c_d	8	5752,61	4672,57	4182,07	4153,18	1,93E-01	30747,20	29254,80	30836,68	28596,47	8,52E-01	4482,23	4737,79	3,91E-01	28952,49	28964,27	2,97E-01	6608,01	5996,90	2,21E-01	29862,13	29209,73	6,26E-01
SPAC1639.01c_b	36	3040,30	2336,28	3191,46	2574,36	7,18E-01	22601,65	17324,84	20994,11	18559,24	9,55E-01	2683,69	2225,63	6,34E-01	18161,07	15445,45	3,98E-01	3396,89	2721,15	5,27E-01	18781,26	15178,14	4,49E-01
SPAC1639.02c_a	8	803,41	699,41	770,69	652,58	6,63E-01	8149,00	7618,27	8115,58	7470,82	8,49E-01	760,08	744,43	9,89E-01	9759,33	8699,87	1,51E-01	792,35	1016,93	3,41E-01	8534,80	7951,05	4,59E-01
SPAC1687.03c_b	6	294,07	256,00	254,23	224,41	2,78E-01	3626,04	3295,87	4069,87	3904,49	1,04E-01	254,23	235,57	2,91E-01	3760,25	3374,79	7,15E-01	317,37	243,88	9,05E-01	3954,35	3169,26	8,35E-01
SPAC1687.11_c	6	266,87	194,01	284,05	224,41	6,64E-01	3456,82	3501,08	3697,00	3767,38	2,59E-02	300,25	296,11	2,05E-01	3329,94	3327,71	2,11E-02	354,59	250,73	3,73E-01	4232,87	3777,98	1,48E-01
SPAC1687.15_b	21	382,68	294,07	458,25	352,14	4,36E-01	24322,55	20951,33	23517,09	21668,65	9,84E-01	407,31	344,89	5,58E-01	20903,61	19608,91	3,18E-01	369,65	335,46	7,93E-01	18052,46	17315,82	1,03E-01
SPAC1687.21_a	6	494,56	439,59	530,06	477,71	4,34E-01	7038,64	6815,17	7020,42	7023,34	4,85E-01	526,39	504,95	2,41E-01	6872,19	7270,43	5,92E-01	739,29	861,08	3,79E-02	8305,44	10007,39	1,22E-01
SPAC16A10.04_c	4	317,37	317,37	342,51	354,59	3,55E-02	9293,80	9973,14	10001,16	8482,79	6,84E-01	451,94	442,64	1,28E-03	10452,79	9927,04	3,25E-01	333,14	427,57	3,14E-01	7182,05	7360,78	2,14E-02
SPAC16A10.04_d	4	433,53	427,57	792,35	613,11	9,36E-02	9280,58	9946,68	10015,87	8480,89	7,05E-01	831,75	724,08	2,33E-02	10441,20	9946,68	2,97E-01	685,02	694,58	4,72E-04	7181,15	7383,04	2,16E-02
SPAC16A10.06c_a	5	136,24	144,01	163,14	125,37	8,50E-01	1365,95	1350,54	1256,41	1138,20	1,14E-01	112,21	135,30	3,11E-01	1024,26	989,37	2,93E-03	116,97	112,21	3,04E-02	1199,92	1367,53	4,69E-01
SPAC16A10.06c_b	10	576,03	461,44	508,46	421,68	5,33E-01	1365,95	1350,54	1256,41	1138,20	1,14E-01	369,65	364,56	1,18E-01	1024,26	989,37	2,93E-03	451,94	451,94	3,64E-01	1199,92	1367,53	4,69E-01
SPAC16C9.01c_a	5	268,73	232,32	427,57	494,56	3,13E-02	2759,13	2998,45	4269,94	4451,27	1,01E-02	385,34	396,18	1,78E-02	5442,30	5042,77	9,57E-03	344,89	362,04	3,61E-02	3769,09	3691,52	2,11E-02
SPAC16E8.01_a	7	760,08	548,75	803,41	760,08	3,59E-01	14381,86	16061,78	13423,70	14887,57	4,40E-01	666,29	699,41	8,15E-01	12773,83	15132,99	4,73E-01	975,50	867,07	1,54E-01	12912,04	14598,46	3,43E-01
SPAC16E8.03_a	4	261,38	243,88	222,86	340,14	6,74E-01	1503,90	1634,20	2582,43	2901,27	2,09E-02	263,20	202,25	5,94E-01	2849,33	2743,04	4,66E-03	230,72	259,57	7,01E-01	2516,28	2479,67	5,27E-03
SPAC16E8.12c_a	13	337,79	284,05	481,04	364,56	2,23E-01	2936,74	2336,28	2977,74	2556,58	7,56E-01	501,46	413,00	1,06E-01	2538,92	2194,99	5,17E-01	491,14	380,04	1,81E-01	3061,45	2452,44	8,05E-01
SPAC16E8.14c_b	4	407,31	369,65	369,65	310,83	3,01E-01	2924,80	2780,51	3076,93	2671,63	9,29E-01	430,54	357,05	9,09E-01	2792,16	2215,56	3,61E-01	335,46	324,03	9,63E-02	3887,23	3483,33	6,04E-02
SPAC16E8.14c_c	4	142,02	164,28	163,14	156,50	6,24E-01	2924,80	2780,51	3076,93	2671,63	9,29E-01	156,50	141,04	7,77E-01	2792,16	2215,56	3,61E-01	172,45	200,85	2,05E-01	3887,23	3483,33	6,04E-02
SPAC16E8.16_a	5	1833,01	1746,20	2288,20	1782,89	4,39E-01	8736,12	7822,23	7645,07	6210,75	2,53E-01	4608,24	4096,00	1,01E-02	17102,55	12859,70	9,08E-02	4240,45	3590,58	2,30E-02	15535,99	13165,91	4,11E-02
SPAC16E8.18c_a	5	388,02	292,04	367,09	319,57	9,56E-01	2365,23	1835,23	2691,21	2012,75	6,18E-01	407,31	278,20	9,76E-01	2879,88	2389,14	2,77E-01	415,87	410,15	2,68E-01	2449,92	1991,53	7,64E-01
SPAC1705.02_a	8	256,00	229,13	245,57	213,78	5,99E-01	1148,04	1004,82	838,78	1082,42	4,99E-01	216,77	233,94	3,93E-01	1248,84	1029,70	6,79E-01	216,77	215,27	1,87E-01	1051,94	903,97	4,40E-01
SPAC1751.03_b	7	2452,44	2401,97	4153,18	4124,49	2,87E-04	5792,62	6122,90	5442,30	5442,30	8,91E-02	3848,29	3258,52	6,26E-02	6841,04	6295,04	1,96E-01	5792,62	7231,10	2,97E-02	7082,29	7434,40	3,28E-02
SPAC1751.04_b	17	1074,91	910,17	1052,79	873,10	8,31E-01	2720,51	2507,69	2515,86	2051,93	3,25E-01	814,63	754,83	1,41E-01	1698,73	1762,67	1,55E-02	1152,06	1332,57	1,78E-01	2137,77	2857,03	7,85E-01

SPAC1782.07_a	8	467,88	364,56	689,78	592,22	8,71E-02	6080,61	5518,27	9345,14	7696,57	8,90E-02	621,67	580,04	8,02E-02	9280,58	8135,41	4,49E-02	820,30	548,75	2,06E-01	10155,69	8659,09	4,57E-02
SPAC1782.07_c	4	477,71	359,54	604,67	617,37	8,36E-02	6080,61	5518,27	9345,14	7696,57	8,90E-02	433,53	556,41	4,65E-01	9280,58	8135,41	4,49E-02	439,59	415,87	8,94E-01	10155,69	8659,09	4,57E-02
SPAC1782.11_a	4	1509,65	1144,10	962,07	608,87	1,67E-01	44686,66	45720,04	43449,84	42820,35	7,59E-02	530,06	548,75	5,00E-02	39030,84	41578,66	7,05E-02	1120,56	1370,04	7,48E-01	38338,48	41123,69	6,64E-02
SPAC1783.02c_c	7	168,90	192,67	194,01	203,66	2,95E-01	2707,84	2641,33	2765,06	2542,41	8,74E-01	207,94	184,82	4,46E-01	2858,79	2506,08	9,69E-01	200,85	205,07	2,08E-01	3509,14	3155,70	6,73E-02
SPAC17A2.05_a	12	621,67	699,41	714,11	639,15	7,94E-01	12884,08	13251,22	14650,32	15572,09	5,42E-02	661,68	588,13	5,74E-01	13567,14	15008,70	2,43E-01	837,53	809,00	5,91E-02	9531,50	9578,38	2,76E-03
SPAC17A2.13c_a	32	4938,99	4240,45	6608,01	5873,48	8,27E-02	31433,17	31871,96	32316,87	31871,96	2,93E-01	6472,02	5673,41	1,08E-01	32093,64	33225,42	2,39E-01	7643,41	7967,99	1,40E-02	31000,42	31216,04	1,56E-01
SPAC17A5.07c_d	5	1552,09	1269,46	1024,00	770,69	1,14E-01	2100,99	2188,66	2169,13	2212,15	4,47E-01	1120,56	797,86	1,70E-01	2195,01	2320,74	2,78E-01	1468,37	1052,79	6,11E-01	2172,15	2196,91	4,75E-01
SPAC17A5.13_b	4	388,02	304,44	407,31	464,65	2,19E-01	3956,48	3147,52	4359,66	3615,55	5,11E-01	491,14	436,55	1,43E-01	3666,02	2856,44	6,62E-01	754,83	572,05	8,74E-02	4039,61	2740,08	8,52E-01
SPAC17A5.16_f	4	191,34	177,29	200,85	210,84	1,30E-01	9580,60	8139,70	9305,40	8344,71	9,71E-01	196,72	146,02	6,71E-01	8497,60	7670,32	4,49E-01	190,02	216,77	3,34E-01	8050,18	7219,62	2,79E-01
SPAC17C9.01c_b	4	164,28	178,53	138,14	159,79	2,25E-01	351,30	329,13	440,03	493,95	4,90E-02	144,01	147,03	7,09E-02	674,59	654,82	2,09E-03	181,02	137,19	6,47E-01	449,09	360,76	2,91E-01
SPAC17C9.08_b	4	286,03	233,94	243,88	245,57	6,17E-01	2436,48	2485,75	2420,55	2478,17	7,86E-01	407,31	380,04	4,51E-02	2865,07	2648,45	1,17E-01	364,56	382,68	5,41E-02	2739,45	2509,21	3,00E-01
SPAC17C9.11c_a	6	282,09	304,44	362,04	300,25	3,68E-01	4174,68	4159,33	3934,37	3951,63	2,65E-03	280,14	282,09	3,92E-01	4924,31	4598,91	6,75E-02	335,46	481,04	2,59E-01	4643,83	4699,55	3,26E-03
SPAC17C9.11c_b	6	219,79	205,07	306,55	247,28	1,69E-01	4182,07	4153,18	3929,15	3956,48	7,73E-03	203,66	215,27	7,81E-01	4938,99	4608,24	6,75E-02	270,60	268,73	1,64E-02	4640,29	4705,07	4,89E-03
SPAC17C9.12_b	10	1520,15	1287,18	820,30	661,68	4,24E-02	14543,94	13399,81	12881,90	12277,74	1,64E-01	897,64	897,64	4,91E-02	12570,79	13066,88	2,06E-01	996,00	1200,98	1,88E-01	13523,43	12328,62	3,33E-01
SPAC17C9.14_a	5	533,74	564,18	484,38	461,44	5,74E-02	2249,74	1994,29	2184,20	1881,03	6,96E-01	487,75	588,13	8,53E-01	1953,18	1864,50	2,56E-01	1002,93	942,27	6,35E-03	1695,97	1616,03	7,35E-02
SPAC17D4.01_a	10	243,88	240,52	199,47	183,55	2,48E-02	18680,01	17036,04	17969,43	17486,93	8,93E-01	263,20	186,11	6,94E-01	18096,98	17724,05	9,56E-01	265,03	296,11	1,34E-01	23237,32	21041,60	8,91E-02
SPAC17D4.01_b	5	680,29	541,19	564,18	508,46	4,25E-01	18680,01	17036,04	17969,43	17486,93	8,93E-01	942,27	849,22	7,64E-02	18096,98	17724,05	9,56E-01	1629,26	1640,59	4,61E-03	23237,32	21041,60	8,91E-02
SPAC17G6.12_c	4	184,82	173,65	172,45	178,53	6,16E-01	1935,64	2573,31	1922,44	2425,94	8,62E-01	186,11	173,65	9,46E-01	2287,70	2895,09	5,24E-01	195,36	154,34	8,56E-01	2366,10	2790,39	4,87E-01
SPAC17G6.12_d	7	263,20	270,60	300,25	292,04	3,39E-02	1935,64	2573,31	1922,44	2425,94	8,62E-01	572,05	401,71	1,23E-01	2287,70	2895,09	5,24E-01	803,41	670,92	1,93E-02	2366,10	2790,39	4,87E-01
SPAC17G6.16c_d	5	337,79	359,54	415,87	404,50	3,75E-02	10013,87	9380,32	10571,05	10031,22	2,84E-01	477,71	352,14	4,08E-01	10968,86	11371,45	5,92E-02	398,93	439,59	9,21E-02	11094,84	10498,00	1,27E-01
SPAC17G8.04c_b	4	210,84	179,77	266,87	233,94	1,35E-01	7967,99	6888,62	7912,95	6472,02	8,18E-01	174,85	254,23	6,96E-01	9741,98	9026,81	9,43E-02	310,83	313,00	1,74E-02	7967,99	7486,11	6,63E-01
SPAC17G8.08c_a	11	344,89	268,73	382,68	290,02	6,71E-01	5998,20	5848,17	8348,84	7360,09	6,10E-02	385,34	321,80	4,45E-01	7365,66	6594,78	1,15E-01	608,87	519,15	4,86E-02	8347,50	8477,91	1,59E-03
SPAC17G8.09_a	4	249,00	254,23	300,25	209,38	9,50E-01	1188,85	1329,83	1209,19	1405,27	7,30E-01	227,54	207,94	7,92E-02	1556,20	1576,88	4,98E-02	276,28	288,01	4,15E-02	1213,27	1343,68	8,60E-01
SPAC17G8.13c_b	4	218,27	209,38	256,00	259,57	1,17E-02	3693,36	3427,61	3107,72	3114,02	7,74E-02	256,00	160,90	9,21E-01	3142,66	3029,76	8,15E-02	315,17	280,14	4,35E-02	4045,65	3853,66	1,41E-01
SPAC17G8.15_a	4	393,44	451,94	280,14	261,38	3,85E-02	7979,22	7778,95	6795,53	6021,40	6,66E-02	290,02	342,51	1,14E-01	5476,65	5698,39	4,22E-03	238,86	315,17	9,38E-02	6013,55	7365,88	2,24E-01
SPAC17G8.15_b	4	232,32	210,84	205,07	172,45	2,35E-01	7979,22	7778,95	6795,53	6021,40	6,66E-02	233,94	200,85	8,52E-01	5476,65	5698,39	4,22E-03	186,11	212,31	3,17E-01	6013,55	7365,88	2,24E-01
SPAC17H9.07_b	5	146,02	174,85	177,29	188,71	2,83E-01	5115,52	4483,33	5231,33	4478,46	9,20E-01	182,28	157,59	6,66E-01	3194,75	3178,78	3,64E-02	172,45	162,02	7,01E-01	5213,76	4635,84	7,97E-01
SPAC17H9.13c_b	5	471,14	464,65	427,57	445,72	8,34E-02	3609,06	3617,55	4028,05	3809,19	1,08E-01	430,54	448,82	1,01E-01	3664,53	3610,15	4,74E-01	588,13	439,59	5,99E-01	3627,37	3412,23	4,77E-01
SPAC1805.07c_a	12	526,39	481,04	418,77	401,71	6,11E-02	2847,47	3117,49	2384,78	2492,24	6,45E-02	643,59	657,11	2,51E-02	3259,95	3458,62	1,54E-01	643,59	820,30	1,29E-01	3278,95	4254,61	2,61E-01
SPAC1805.13_a	10	996,00	685,02	1105,13	982,29	3,48E-01	32995,92	28133,47	34877,28	31651,80	4,52E-01	1009,90	843,36	6,74E-01	31433,17	27554,49	7,63E-01	1251,98	1251,98	1,18E-01	34159,52	29532,18	7,39E-01

SPAC1805.15c_b	4	177,29	188,71	219,79	225,97	2,55E-02	837,53	929,30	803,41	982,29	9,34E-01	238,86	183,55	4,23E-01	1038,29	1038,29	7,77E-02	230,72	286,03	1,16E-01	1184,45	1120,56	4,06E-02
SPAC1834.07_b	8	257,78	237,21	292,04	315,17	6,84E-02	968,76	968,76	968,76	942,27	4,23E-01	313,00	284,05	1,03E-01	1009,90	942,27	8,49E-01	398,93	410,15	5,52E-03	1488,87	1243,34	8,37E-02
SPAC1851.03_b	8	300,25	268,73	393,44	342,51	1,08E-01	12854,63	11268,44	12330,98	10809,41	6,99E-01	308,69	319,57	2,17E-01	13493,72	11665,82	7,10E-01	390,72	382,68	2,44E-02	13969,57	12245,81	4,66E-01
SPAC186.08c_a	5	142,02	150,12	132,51	141,04	2,55E-01	56,55	64,06	57,09	61,27	8,19E-01	146,02	155,42	5,32E-01	73,03	74,25	7,27E-02	155,42	153,28	1,87E-01	100,46	82,34	8,67E-02
SPAC18B11.04_b	4	526,39	421,68	401,71	354,59	2,37E-01	1597,65	1162,79	1336,93	1229,67	7,07E-01	385,34	404,50	2,76E-01	1140,79	966,25	2,98E-01	471,14	544,96	6,49E-01	1294,48	903,54	4,38E-01
SPAC18B11.07c_b	9	826,00	709,18	975,50	1024,00	6,69E-02	28724,62	27175,14	28526,20	27175,14	9,32E-01	855,13	621,67	8,44E-01	27364,16	29944,43	6,86E-01	643,59	639,15	1,63E-01	23987,58	26068,14	1,53E-01
SPAC18B11.08c_a	5	337,79	342,51	413,00	388,02	4,16E-02	15537,92	13419,01	13349,01	13554,19	4,36E-01	390,72	415,87	3,87E-02	12889,90	12671,35	2,52E-01	413,00	390,72	3,24E-02	16717,52	13955,70	6,71E-01
SPAC18B11.09c_a	4	1595,73	1858,60	2048,00	1606,83	7,34E-01	2580,79	2872,69	4568,42	3082,93	2,84E-01	1618,00	1595,73	4,58E-01	2913,24	2523,40	9,76E-01	1640,59	2019,80	6,99E-01	1978,33	2911,58	6,23E-01
SPAC18B11.10_a	8	418,77	461,44	498,00	477,71	1,81E-01	4742,48	4657,87	4590,16	4561,18	1,08E-01	433,53	357,05	4,14E-01	4591,20	4816,63	9,78E-01	380,04	477,71	8,53E-01	4595,33	4156,80	2,84E-01
SPAC18G6.03_a	11	1002,93	996,00	1488,87	1370,04	1,86E-02	32093,64	31216,04	30786,28	29944,43	1,68E-01	1323,37	1184,45	6,73E-02	28526,20	28133,47	2,03E-02	1389,16	1217,75	7,12E-02	26801,01	28724,62	6,65E-02
SPAC18G6.04c_a	5	296,11	347,29	385,34	335,46	3,92E-01	4550,56	4694,37	4166,03	4457,77	1,96E-01	498,00	418,77	1,01E-01	3060,65	3170,80	3,59E-03	657,11	584,07	2,15E-02	3380,58	3230,59	6,17E-03
SPAC1952.01_a	10	814,63	600,49	734,19	564,18	7,11E-01	4843,54	3727,66	4952,57	3683,60	9,73E-01	935,76	1082,39	1,46E-01	3959,61	2795,35	3,77E-01	2120,22	1758,34	2,79E-02	3889,25	3022,21	3,61E-01
SPAC1952.04c_a	7	3590,58	3104,19	3590,58	2272,40	6,14E-01	11190,10	11278,44	12091,60	10852,67	7,39E-01	2592,27	1710,26	1,41E-01	11356,56	9343,17	4,73E-01	3541,14	4182,07	3,29E-01	12041,86	12856,57	9,74E-02
SPAC1952.04c_c	9	522,76	526,39	487,75	556,41	9,49E-01	11190,10	11278,44	12091,60	10852,67	7,39E-01	501,46	424,61	2,51E-01	11356,56	9343,17	4,73E-01	670,92	675,59	3,96E-04	12041,86	12856,57	9,74E-02
SPAC1952.07_a	5	292,04	278,20	296,11	257,78	7,27E-01	1459,97	1572,19	1342,88	1401,15	1,50E-01	296,11	213,78	5,45E-01	1458,66	1396,12	3,01E-01	484,38	461,44	5,05E-03	1698,65	1637,02	1,41E-01
SPAC1952.10c_a	5	1710,26	1562,89	3019,30	2592,27	3,54E-02	5792,62	6122,90	5442,30	5442,30	8,91E-02	3373,43	2896,31	2,67E-02	6841,04	6295,04	1,96E-01	3373,43	3743,05	1,06E-02	7082,29	7434,40	3,28E-02
SPAC1952.13_b	6	625,99	652,58	666,29	680,29	1,52E-01	7003,80	7025,88	7288,13	7744,67	1,59E-01	630,35	596,34	3,52E-01	7651,10	8237,08	8,68E-02	754,83	617,37	5,72E-01	5065,58	4814,21	3,68E-03
SPAC1952.15c_a	6	209,38	210,84	227,54	203,66	6,91E-01	249,43	248,39	229,05	235,28	3,38E-02	232,32	230,72	2,55E-03	367,59	296,71	1,43E-01	218,27	205,07	8,36E-01	552,82	355,01	1,74E-01
SPAC19A8.04_a	15	843,36	831,75	770,69	729,11	5,56E-02	25156,82	24234,35	25356,63	24813,16	5,43E-01	843,36	680,29	4,52E-01	22482,74	23915,27	2,21E-01	1746,20	2062,24	2,13E-02	22228,50	23375,27	1,24E-01
SPAC19B12.06c_b	4	191,34	192,67	191,34	164,28	4,05E-01	17432,81	15035,10	16557,84	15049,90	7,90E-01	162,02	165,42	4,15E-03	14829,95	12648,47	2,64E-01	147,03	221,32	8,53E-01	15555,65	14588,24	4,64E-01
SPAC19B12.08_a	5	1332,57	975,50	942,27	781,44	2,74E-01	7076,79	6425,57	5340,32	4703,50	6,29E-02	867,07	739,29	2,05E-01	5738,26	4898,39	1,14E-01	968,76	661,68	2,87E-01	5546,96	5457,57	6,28E-02
SPAC19B12.10_b	4	719,08	564,18	744,43	714,11	3,82E-01	1771,97	1641,61	1657,00	1633,15	4,50E-01	657,11	724,08	6,20E-01	1596,87	1588,53	2,23E-01	935,76	608,87	5,45E-01	1857,50	1767,06	3,15E-01
SPAC19D5.03_a	4	191,34	124,50	176,07	128,00	8,99E-01	3361,36	3345,30	3444,58	3322,62	6,71E-01	280,14	150,12	5,16E-01	3803,83	3304,99	5,05E-01	199,47	155,42	6,74E-01	5287,99	5385,06	6,15E-04
SPAC19E9.01c_a	7	388,02	385,34	335,46	266,87	1,30E-01	10331,12	8637,14	10263,86	9513,69	7,05E-01	335,46	330,84	2,48E-03	9753,40	9032,52	9,30E-01	302,33	354,59	1,56E-01	9407,00	8306,40	5,98E-01
SPAC19E9.02_a	12	207,94	216,77	227,54	227,54	7,51E-02	11927,52	14624,63	11830,36	12432,50	4,95E-01	265,03	256,00	1,68E-02	13124,42	13997,31	8,59E-01	230,72	265,03	1,83E-01	10734,73	13127,26	5,33E-01
SPAC19G12.08_a	11	4096,00	2721,15	4803,93	3691,52	4,43E-01	33225,42	30573,63	31216,04	29125,60	4,14E-01	4329,55	3350,13	6,60E-01	24322,43	23331,64	2,94E-02	3929,15	3236,01	8,42E-01	25888,07	26068,14	4,69E-02
SPAC19G12.17_b	4	2105,58	1734,13	2574,36	2256,70	1,80E-01	7804,01	6208,38	7912,95	6700,25	7,93E-01	2957,17	1758,34	5,58E-01	7082,29	5832,91	6,42E-01	3191,46	2385,37	1,89E-01	7383,04	6165,49	8,39E-01
SPAC1B1.01_a	8	765,36	512,00	580,04	552,56	6,27E-01	29713,45	28173,07	26166,23	25858,75	6,49E-02	604,67	596,34	7,92E-01	24326,52	24898,71	3,42E-02	1260,69	1144,10	5,61E-02	29724,55	26713,35	7,10E-01
SPAC1B1.04c_f	5	621,67	533,74	661,68	385,34	7,44E-01	15033,17	14664,33	13842,28	13502,15	4,26E-02	467,88	427,57	1,15E-01	12736,46	13539,12	6,06E-02	451,94	401,71	9,66E-02	14183,66	13888,85	7,51E-02
SPAC1B2.04_a	16	600,49	342,51	754,83	484,38	5,11E-01	34877,28	32316,87	32316,87	30362,44	2,96E-01	694,58	471,14	5,81E-01	29944,43	32316,87	2,93E-01	699,41	494,56	5,26E-01	32316,87	33689,23	7,22E-01

SPAC1B3.01c_b	9	1217,75	916,51	1509,65	1031,12	5,47E-01	25623,71	25772,27	26579,51	25612,68	5,01E-01	1287,18	1438,15	2,21E-01	20801,14	20657,32	4,33E-04	3743,05	3565,78	4,53E-03	25346,12	24393,79	2,28E-01
SPAC1B3.02c_a	6	300,25	313,00	891,44	1009,90	8,45E-03	5113,16	4389,98	8719,32	9541,50	1,53E-02	1031,12	1200,98	1,09E-02	9809,75	10884,59	1,31E-02	474,41	436,55	1,75E-02	7750,10	5712,87	2,08E-01
SPAC1B3.04c_a	5	215,27	257,78	249,00	235,57	8,20E-01	863,53	871,65	1004,44	979,12	1,13E-02	243,88	265,03	5,29E-01	835,23	824,01	3,17E-02	209,38	216,77	3,91E-01	1217,77	1001,99	1,54E-01
SPAC1B3.04c_b	4	335,46	308,69	533,74	657,11	4,94E-02	861,08	873,10	1002,93	982,29	8,93E-03	749,61	657,11	1,56E-02	837,53	826,00	5,13E-02	407,31	474,41	8,13E-02	1217,75	1002,93	1,52E-01
SPAC1B3.12c_b	13	608,87	604,67	685,02	533,74	9,76E-01	27165,83	29029,26	27539,94	26047,36	3,89E-01	699,41	714,11	5,79E-03	25200,96	26484,28	1,84E-01	814,63	1024,00	9,63E-02	26986,59	29951,46	8,52E-01
SPAC1B3.13_a	6	1924,14	1640,59	1992,00	1663,49	8,54E-01	5629,28	5612,58	6068,72	6134,20	4,91E-03	2241,11	1782,89	4,84E-01	5013,99	4790,43	2,35E-02	2936,74	2977,74	1,45E-02	6001,60	5777,05	1,40E-01
SPAC1B3.14_c	6	324,03	266,87	298,17	261,38	6,90E-01	23075,29	22905,24	24790,92	23371,30	2,67E-01	286,03	290,02	8,20E-01	23655,32	23334,36	1,09E-01	280,14	477,71	5,02E-01	24480,11	23096,96	3,71E-01
SPAC1B9.02c_c	4	243,88	200,85	250,73	235,57	4,58E-01	6044,40	6421,61	6101,07	6689,66	6,88E-01	207,94	177,29	3,77E-01	5456,44	5861,61	1,74E-01	240,52	274,37	3,28E-01	5066,75	5505,36	8,20E-02
SPAC1D4.02c_b	4	2048,00	1332,57	1136,20	849,22	2,12E-01	19587,66	15863,35	16289,64	14724,24	3,87E-01	1226,22	1112,82	2,87E-01	16828,81	16795,36	6,72E-01	1675,06	1097,50	5,76E-01	15153,24	14508,51	2,65E-01
SPAC1D4.08_a	11	2005,85	1509,65	1629,26	1746,20	8,09E-01	18709,65	17672,60	17353,78	16655,24	1,98E-01	1418,35	1595,73	4,42E-01	15722,69	15090,98	4,44E-02	2418,67	2352,53	1,29E-01	15675,35	14712,66	5,15E-02
SPAC1F12.10c_b	7	294,07	225,97	418,77	362,04	9,87E-02	4240,45	3983,99	6122,90	5752,61	1,49E-02	439,59	467,88	3,44E-02	6382,92	5595,30	4,54E-02	424,61	359,54	1,07E-01	6700,25	6080,61	2,10E-02
SPAC1F3.04c_a	5	634,73	699,41	617,37	552,56	2,15E-01	11652,68	10911,20	11200,70	10682,71	5,30E-01	576,03	634,73	2,93E-01	9860,77	9707,31	5,83E-02	1136,20	1120,56	5,16E-03	11087,24	11981,01	7,06E-01
SPAC1F3.05_c	6	3769,09	3304,00	3615,55	3929,15	4,89E-01	18543,53	17448,63	17026,60	16917,37	2,04E-01	4240,45	3590,58	4,43E-01	13203,19	13827,45	1,92E-02	7131,55	6841,04	6,26E-03	13749,11	13626,11	1,60E-02
SPAC1F3.09_b	5	855,13	694,58	982,29	1120,56	1,21E-01	11425,74	9809,75	11190,60	10369,08	8,75E-01	996,00	809,00	4,09E-01	12416,75	11993,79	1,98E-01	1287,18	1243,34	2,76E-02	10809,41	10226,32	9,18E-01
SPAC1F5.03c_a	9	685,02	625,99	891,44	729,11	2,15E-01	16728,26	15181,22	18053,61	16728,26	2,94E-01	666,29	548,75	5,41E-01	13216,02	11910,94	7,87E-02	897,64	617,37	5,50E-01	15286,81	14868,79	3,88E-01
SPAC1F7.04_a	14	1351,18	1016,93	1698,45	1296,13	3,54E-01	35364,15	33923,56	34159,52	32995,92	3,69E-01	1618,00	1370,04	2,75E-01	31433,17	32093,64	6,80E-02	1924,14	1458,23	2,19E-01	31000,42	30362,44	3,73E-02
SPAC1F7.05_a	14	1296,13	1351,18	1509,65	1746,20	1,29E-01	15935,98	17318,18	17079,76	18305,63	3,68E-01	1897,65	2048,00	1,49E-02	14562,80	15716,58	2,40E-01	2896,31	2702,35	4,63E-03	17438,64	18305,63	2,67E-01
SPAC20G4.06c_a	6	347,29	249,00	364,56	304,44	5,93E-01	32503,12	30563,39	30684,03	29435,88	3,30E-01	282,09	290,02	8,29E-01	28660,51	29574,56	1,53E-01	407,31	401,71	1,63E-01	25791,49	24470,54	3,20E-02
SPAC20G8.05c_b	8	494,56	541,19	630,35	734,19	1,02E-01	3875,05	4211,15	5042,77	4837,35	4,50E-02	760,08	666,29	6,50E-02	5752,61	5518,27	1,62E-02	754,83	776,05	1,05E-02	3743,05	3769,09	2,31E-01
SPAC20H4.03c_c	7	680,29	544,96	572,05	427,57	3,73E-01	8454,16	7465,24	7743,46	7112,12	4,60E-01	625,99	572,05	8,69E-01	6489,51	6509,80	9,81E-02	903,89	996,00	5,41E-02	6875,11	6818,20	1,54E-01
SPAC20H4.11c_a	4	120,26	107,63	157,59	137,19	1,08E-01	948,83	1105,13	935,76	843,36	2,69E-01	178,53	152,22	7,19E-02	1468,37	1002,93	4,85E-01	142,02	127,12	1,69E-01	1287,18	1128,35	2,46E-01
SPAC21E11.05c_b	6	203,66	233,94	206,50	227,54	9,32E-01	1436,59	1286,01	1563,31	1466,90	2,28E-01	230,72	232,32	4,90E-01	1362,13	1184,75	5,29E-01	247,28	235,57	2,98E-01	1387,41	1150,39	5,78E-01
SPAC222.03c_a	15	814,63	588,13	1024,00	809,00	3,02E-01	20594,91	19349,38	21920,61	20452,65	3,34E-01	903,89	837,53	2,88E-01	21321,18	20882,40	2,29E-01	855,13	903,89	2,64E-01	20171,07	20311,37	7,09E-01
SPAC222.09_a	7	1089,92	1052,79	1897,65	1629,26	3,62E-02	23987,58	22851,48	29125,60	26432,04	9,64E-02	1845,76	1770,57	3,22E-03	25355,30	24661,96	1,40E-01	1992,00	2076,59	2,29E-03	22073,07	20031,74	1,80E-01
SPAC222.13c_c	5	298,17	306,55	340,14	388,02	1,26E-01	2143,26	2286,04	2393,21	2472,03	1,16E-01	335,46	347,29	3,28E-02	2631,01	2632,14	2,81E-02	306,55	390,72	3,88E-01	2207,93	2249,56	8,67E-01
SPAC222.14c_a	4	133,44	141,04	138,14	156,50	4,17E-01	5770,53	6410,39	5933,45	5802,82	5,66E-01	114,56	129,79	2,19E-01	5338,57	5887,77	3,75E-01	162,02	108,38	9,47E-01	4908,71	5477,29	1,71E-01
SPAC222.14c_c	4	284,05	247,28	374,81	224,41	7,04E-01	5770,53	6410,39	5933,45	5802,82	5,66E-01	252,48	286,03	8,99E-01	5338,57	5887,77	3,75E-01	313,00	235,57	8,59E-01	4908,71	5477,29	1,71E-01
SPAC222.15_d	5	139,10	167,73	162,02	145,01	9,96E-01	63,98	75,76	62,78	76,02	9,63E-01	139,10	141,04	4,51E-01	99,53	76,67	2,92E-01	184,82	147,03	6,50E-01	92,76	75,36	3,09E-01
SPAC227.11c_a	5	200,85	149,09	210,84	195,36	4,07E-01	5641,90	4500,00	5149,72	4125,81	6,29E-01	203,66	187,40	5,28E-01	5281,44	4180,78	7,10E-01	243,88	237,21	1,29E-01	5240,75	3949,75	6,36E-01
SPAC227.19c_a	4	1038,29	975,50	2556,58	1845,76	7,88E-02	3281,18	2556,58	6382,92	5404,70	3,94E-02	2194,99	2164,77	8,81E-04	5518,27	5042,77	3,21E-02	1488,87	1234,75	1,13E-01	3019,30	2721,15	9,13E-01

SPAC22E12.02_a	8	564,18	474,41	897,64	837,53	2,32E-02	4240,45	3640,70	3956,48	3640,70	7,16E-01	922,88	809,00	4,11E-02	4182,07	3169,41	6,97E-01	820,30	734,19	5,35E-02	4451,27	3743,05	7,68E-01
SPAC22E12.04_b	6	237,21	194,01	315,17	247,28	2,44E-01	14664,09	12765,83	15181,22	13400,51	7,01E-01	252,48	270,60	1,89E-01	15608,02	13682,08	5,63E-01	272,48	313,00	1,21E-01	14164,58	12765,83	8,52E-01
SPAC22E12.13c_a	9	413,00	374,81	390,72	445,72	5,43E-01	16190,93	15154,13	17151,07	15680,93	4,95E-01	413,00	481,04	3,06E-01	13053,50	14188,33	1,16E-01	526,39	487,75	5,31E-02	17089,90	15863,39	4,22E-01
SPAC22E12.16c_a	6	2418,67	2435,50	2876,30	2538,92	2,39E-01	3436,83	3702,49	3176,73	3508,33	3,97E-01	3640,70	3147,52	5,94E-02	3570,67	3640,11	8,19E-01	4870,99	5955,47	3,14E-02	3902,41	4061,36	1,17E-01
SPAC22F3.09c_a	6	377,41	276,28	410,15	407,31	2,47E-01	1770,57	2076,59	1897,65	2210,26	6,12E-01	385,34	498,00	2,69E-01	3040,30	3281,18	2,39E-02	442,64	487,75	1,30E-01	2134,97	2418,67	2,33E-01
SPAC22F3.10c_a	10	396,18	385,34	427,57	433,53	2,33E-02	3963,53	4001,88	4667,84	4781,39	6,46E-03	474,41	477,71	4,38E-03	4644,30	4803,09	1,19E-02	461,44	552,56	1,27E-01	4553,84	4692,48	1,24E-02
SPAC22F3.15_a	5	458,25	413,00	760,08	694,58	1,81E-02	4329,55	3743,05	4039,61	3929,15	8,78E-01	680,29	548,75	1,24E-01	2033,85	2210,26	2,46E-02	809,00	685,02	4,21E-02	3492,39	2957,17	1,78E-01
SPAC22G7.01c_a	4	494,56	313,00	458,25	427,57	7,12E-01	18866,76	21114,12	19849,08	21168,46	7,29E-01	415,87	369,65	9,17E-01	16684,64	18070,83	1,86E-01	415,87	512,00	6,17E-01	14903,21	16007,54	6,85E-02
SPAC22G7.04_c	5	448,82	474,41	455,09	467,88	9,93E-01	9087,51	9481,26	9192,96	9137,46	6,10E-01	317,37	526,39	7,42E-01	8383,00	8873,33	1,72E-01	487,75	491,14	1,64E-01	8560,30	9488,85	6,58E-01
SPAC22G7.05_a	5	154,34	167,73	187,40	166,57	3,27E-01	2279,97	2111,15	2351,82	2368,81	1,92E-01	196,72	166,57	3,38E-01	2053,38	2086,16	2,81E-01	132,51	176,07	7,95E-01	2491,70	2533,42	6,77E-02
SPAC22H10.02_b	5	2019,80	2033,85	2288,20	2194,99	4,49E-02	3388,53	3580,01	3753,37	3486,73	4,95E-01	1858,60	2019,80	3,92E-01	3341,09	3124,06	2,24E-01	2368,90	2683,69	8,68E-02	2899,30	3212,00	1,44E-01
SPAC22H10.04_b	4	344,89	294,07	401,71	278,20	7,88E-01	2414,92	2209,15	2853,74	2652,24	9,21E-02	296,11	284,05	3,77E-01	2930,83	2837,71	3,68E-02	364,56	347,29	3,07E-01	2795,05	2400,82	3,27E-01
SPAC22H12.02_a	8	754,83	680,29	1160,07	1052,79	2,71E-02	4153,18	3640,70	5518,27	5518,27	2,41E-02	1296,13	1314,23	4,23E-03	6746,86	6251,56	1,82E-02	689,78	652,58	3,81E-01	3902,01	3191,46	5,08E-01
SPAC23A1.03_a	7	1176,27	1031,12	935,76	604,67	2,06E-01	4326,65	3910,60	4566,96	4314,89	3,16E-01	975,50	744,43	2,16E-01	3545,23	2861,46	1,49E-01	2320,15	2385,37	4,03E-03	4260,24	3585,18	6,70E-01
SPAC23A1.06c_a	13	474,41	415,87	504,95	372,22	9,36E-01	11758,87	11117,82	12208,12	11233,29	6,76E-01	572,05	424,61	5,72E-01	11641,97	10378,88	6,07E-01	634,73	512,00	2,00E-01	11849,62	11237,36	8,35E-01
SPAC23A1.19c_a	7	321,80	300,25	436,55	491,14	3,50E-02	2385,37	2469,49	2352,53	2401,97	4,12E-01	519,15	382,68	1,80E-01	3304,00	3326,99	2,40E-03	455,09	372,22	1,39E-01	2646,74	2916,45	1,29E-01
SPAC23C11.08_a	6	996,00	989,12	1398,83	1488,87	9,86E-03	10085,54	9152,82	9410,14	9152,82	5,57E-01	1458,23	1105,13	2,43E-01	7967,99	7131,55	8,07E-02	2574,36	1937,53	5,81E-02	10369,08	9152,82	8,70E-01
SPAC23C11.08_c	7	792,35	685,02	849,22	724,08	6,20E-01	10097,90	9125,17	9398,78	9137,84	5,66E-01	770,69	613,11	6,72E-01	7945,49	7118,84	8,27E-02	1136,20	968,76	8,75E-02	10389,57	9141,55	8,64E-01
SPAC23C11.11_b	6	302,33	296,11	313,00	300,25	4,07E-01	11794,92	10375,00	9827,82	10323,92	3,12E-01	372,22	286,03	5,60E-01	9751,68	9921,95	2,23E-01	354,59	280,14	6,75E-01	10653,65	9288,29	3,75E-01
SPAC23C11.13c_a	12	1045,52	803,41	935,76	661,68	5,63E-01	37513,91	37508,12	37361,49	35168,59	3,74E-01	948,83	760,08	6,93E-01	28980,27	31650,71	3,28E-02	1520,15	1478,58	4,27E-02	32119,64	34447,01	6,81E-02
SPAC23C4.08_b	13	675,59	541,19	809,00	494,56	8,23E-01	28567,56	27993,23	29251,06	27513,75	9,21E-01	1045,52	634,73	3,96E-01	27437,02	27278,27	9,03E-02	1389,16	942,27	1,40E-01	27008,89	27767,97	2,02E-01
SPAC23C4.09c_a	5	218,27	202,25	263,20	218,27	3,30E-01	3310,06	3383,30	4176,69	3820,50	6,97E-02	215,27	192,67	6,94E-01	3523,56	3569,02	4,36E-02	224,41	259,57	2,42E-01	3713,86	3197,55	7,16E-01
SPAC23C4.09c_d	10	196,72	155,42	151,17	165,42	5,01E-01	3310,06	3383,30	4176,69	3820,50	6,97E-02	173,65	173,65	9,17E-01	3523,56	3569,02	4,36E-02	151,17	179,77	7,14E-01	3713,86	3197,55	7,16E-01
SPAC23C4.12_a	5	491,14	427,57	613,11	306,55	9,98E-01	8956,03	8308,21	9294,98	7488,67	8,26E-01	621,67	407,31	6,71E-01	8736,90	8288,69	7,91E-01	1002,93	689,78	1,36E-01	8834,96	7733,68	6,41E-01
SPAC23C4.15_b	9	436,55	421,68	648,07	349,71	6,86E-01	9531,47	9128,37	10488,04	9491,50	3,44E-01	680,29	709,18	3,72E-03	8588,29	8429,84	6,31E-02	1398,83	1269,46	5,14E-03	10353,02	10298,43	3,93E-02
SPAC23D3.14c_b	5	139,10	192,67	158,68	174,85	9,78E-01	1397,14	1306,80	1138,40	1086,90	4,41E-02	152,22	140,07	5,47E-01	1506,03	1382,23	3,52E-01	130,69	141,04	3,86E-01	1901,89	1761,71	2,89E-02
SPAC23E2.02_a	6	176,07	213,78	240,52	227,54	1,89E-01	3737,88	3887,56	4175,16	3773,05	5,30E-01	186,11	247,28	6,06E-01	4783,21	4587,39	1,94E-02	210,84	202,25	6,09E-01	3995,28	4013,42	1,26E-01
SPAC23G3.02c_a	10	2574,36	2538,92	1663,49	1858,60	1,52E-02	3548,65	3734,71	2452,30	2851,76	4,62E-02	1910,85	2469,49	3,21E-01	2980,62	3524,20	3,08E-01	2241,11	2320,15	2,37E-02	3324,87	3784,82	7,60E-01
SPAC23G3.07c_a	6	689,78	666,29	855,13	879,17	7,81E-03	7231,10	7181,15	7131,55	6936,54	2,29E-01	929,30	922,88	2,40E-03	7181,15	6936,54	3,59E-01	942,27	897,64	1,07E-02	7131,55	7383,04	7,28E-01
SPAC23H3.13c_a	17	1640,59	1314,23	1323,37	1305,15	4,23E-01	4829,49	4195,79	3721,78	3465,72	1,15E-01	1351,18	1278,29	4,33E-01	3854,23	3864,89	1,75E-01	1722,16	1418,35	7,18E-01	4300,88	4227,37	5,17E-01

SPAC23H3.14_b	4	724,08	442,64	648,07	533,74	9,65E-01	8284,97	6994,57	7502,12	6654,07	5,43E-01	613,11	418,77	7,31E-01	6947,47	6937,94	3,93E-01	1089,92	734,19	2,84E-01	7031,96	6740,23	3,73E-01
SPAC23H4.02_a	7	5996,90	4904,87	4482,23	4576,41	2,35E-01	21080,81	20295,56	16209,55	17065,49	1,99E-02	3743,05	3848,29	9,45E-02	13615,43	15018,32	1,55E-02	5673,41	4870,99	8,17E-01	15501,20	15400,62	5,66E-03
SPAC23H4.03c_a	5	284,05	292,04	319,57	296,11	2,51E-01	7671,98	7451,02	7974,98	7533,37	5,17E-01	357,05	324,03	9,07E-02	7209,12	6919,39	1,12E-01	530,06	410,15	9,38E-02	6049,40	6502,55	3,64E-02
SPAC23H4.16c_b	5	948,83	600,49	1305,15	996,00	2,48E-01	9152,82	7590,61	8599,28	7590,61	7,94E-01	1428,22	1074,91	1,94E-01	9026,81	8539,88	6,65E-01	1379,57	1209,34	1,15E-01	7750,10	8023,41	6,03E-01
SPAC23H4.18c_a	7	145,01	147,03	156,50	148,06	2,86E-01	669,46	686,28	958,97	948,71	1,27E-03	144,01	160,90	5,28E-01	980,68	889,36	3,11E-02	155,42	152,22	5,42E-02	1032,90	792,58	1,90E-01
SPAC24C9.06c_a	28	1652,00	1488,87	1770,57	1686,71	2,27E-01	29247,60	29494,93	31687,66	30800,99	5,54E-02	2048,00	2149,82	3,15E-02	31290,34	32681,09	6,59E-02	2401,97	2225,63	2,51E-02	32523,09	33018,05	6,56E-03
SPAC24C9.16c_b	8	814,63	776,05	809,00	613,11	4,87E-01	6866,90	6168,21	7021,94	5965,69	9,73E-01	719,08	749,61	1,31E-01	6760,40	6100,44	8,73E-01	1573,76	1243,34	6,63E-02	9326,23	6934,37	3,25E-01
SPAC25G10.05c_a	6	855,13	689,78	704,28	608,87	3,49E-01	38637,45	39090,16	37934,99	36659,41	1,47E-01	729,11	657,11	4,72E-01	29259,20	28300,16	2,75E-03	1314,23	1067,48	1,06E-01	33642,66	32047,40	1,85E-02
SPAC25G10.06_a	6	754,83	448,82	661,68	548,75	9,85E-01	33780,90	32467,09	33300,88	32478,39	7,91E-01	471,14	380,04	3,85E-01	28129,57	29728,96	5,58E-02	504,95	634,73	8,65E-01	29130,90	25382,94	9,80E-02
SPAC26A3.02_a	4	294,07	369,65	424,61	347,29	4,22E-01	5723,41	7348,84	5874,49	5791,34	4,79E-01	225,97	464,65	9,24E-01	5327,25	6263,18	5,12E-01	259,57	333,14	5,70E-01	5707,29	6960,39	8,62E-01
SPAC26A3.06_b	4	188,71	177,29	247,28	290,02	6,07E-02	8306,36	8248,98	9152,82	8023,41	6,38E-01	179,77	296,11	4,46E-01	8964,45	7231,10	8,55E-01	265,03	282,09	1,26E-02	10226,32	9345,14	7,61E-02
SPAC26A3.10_b	12	504,95	481,04	530,06	508,46	2,45E-01	3528,62	3506,56	3235,31	3602,43	6,45E-01	826,00	903,89	1,18E-02	4889,92	4786,18	1,61E-03	837,53	776,05	1,09E-02	4485,59	4305,93	1,05E-02
SPAC26H5.07c_a	11	1937,53	1269,46	2005,85	1871,53	4,29E-01	31000,42	29125,60	30152,71	28329,16	5,94E-01	1992,00	1833,01	4,63E-01	26432,04	26068,14	5,74E-02	3590,58	2998,45	6,31E-02	25888,07	26801,01	7,04E-02
SPAC27E2.10c_b	5	576,03	576,03	533,74	491,14	9,63E-02	1027,09	971,39	1157,55	1074,94	1,43E-01	430,54	362,04	3,44E-02	1113,14	971,83	6,27E-01	797,86	652,58	1,76E-01	1379,84	1111,17	2,15E-01
SPAC27E2.10c_e	5	1418,35	1488,87	1152,06	1002,93	4,49E-02	1027,09	971,39	1157,55	1074,94	1,43E-01	1468,37	820,30	4,43E-01	1113,14	971,83	6,27E-01	1924,14	1698,45	9,41E-02	1379,84	1111,17	2,15E-01
SPAC27E2.12_a	7	170,07	209,38	163,14	229,13	8,83E-01	275,46	275,17	214,86	328,29	9,53E-01	227,54	210,84	3,02E-01	340,02	260,26	5,97E-01	205,07	205,07	5,17E-01	277,32	176,65	4,38E-01
SPAC29A4.02c_a	12	1795,29	1618,00	2179,83	2164,77	3,46E-02	34159,52	32995,92	33923,56	32768,00	8,04E-01	2241,11	2256,70	2,59E-02	32768,00	33456,53	5,62E-01	3258,52	3691,52	1,71E-02	32093,64	31433,17	1,13E-01
SPAC29A4.08c_b	20	357,05	308,69	335,46	290,02	6,06E-01	7242,84	6254,73	7077,43	6148,82	8,60E-01	382,68	317,37	7,14E-01	5978,43	4839,04	2,18E-01	467,88	404,50	1,22E-01	7131,09	6331,26	9,80E-01
SPAC29A4.14c_b	4	639,15	537,45	729,11	584,07	5,21E-01	3963,77	3223,09	3507,66	2668,42	4,62E-01	608,87	568,10	9,98E-01	3714,92	3081,85	7,28E-01	1160,07	765,36	2,08E-01	3984,82	3252,15	9,66E-01
SPAC29B12.08_a	25	7804,01	7538,18	6338,83	5792,62	3,40E-02	31058,98	31259,01	25542,93	25547,93	3,17E-04	5518,27	4737,79	2,53E-02	22824,34	23653,73	2,89E-03	5367,37	5184,54	4,51E-03	25119,53	26869,84	2,79E-02
SPAC2E12.02_a	14	1360,57	14766,09	1488,87	16961,78	9,20E-01	14622,83	1428,22	15182,78	1370,04	9,81E-01	1734,13	21469,49	7,95E-01	14351,79	1845,76	9,94E-01	1845,76	22536,88	7,70E-01	13527,08	1910,85	9,75E-01
SPAC2F3.06c_a	4	390,72	935,76	436,55	996,00	9,04E-01	10884,59	14391,90	11346,82	14472,78	9,19E-01	308,69	1152,06	9,06E-01	10809,41	14405,15	9,91E-01	487,75	1323,37	6,75E-01	10015,87	14602,90	9,20E-01
SPAC2F3.18c_a	5	1209,34	354,59	1418,35	487,75	8,12E-01	7175,93	11268,44	7450,27	12161,22	8,69E-01	1287,18	519,15	8,52E-01	6402,01	10809,41	8,57E-01	2797,65	508,46	5,50E-01	7836,93	10369,08	9,65E-01
SPAC2F3.18c_b	4	962,07	1097,50	1074,91	1351,18	3,56E-01	7181,15	7533,97	7434,40	6480,78	5,14E-01	891,44	1562,89	6,23E-01	6382,92	6504,49	3,92E-02	1758,34	3492,39	2,08E-01	7858,29	8718,52	1,83E-01
SPAC2F7.17_a	5	263,20	734,19	349,71	982,29	7,13E-01	699,52	7538,18	697,55	6472,02	9,16E-01	369,65	942,27	7,13E-01	607,37	6517,03	9,13E-01	324,03	1782,89	5,44E-01	697,24	8719,32	9,21E-01
SPAC2F7.17_b	7	188,71	280,14	173,65	221,32	5,48E-01	699,52	731,58	697,55	718,31	7,28E-01	186,11	315,17	8,56E-01	607,37	611,88	2,26E-02	232,32	342,51	5,36E-01	697,24	635,81	2,93E-01
SPAC2G11.06_b	4	259,57	156,50	306,55	133,44	9,16E-01	9877,98	731,58	9410,14	718,31	9,73E-01	349,71	190,02	5,82E-01	9089,59	611,88	9,49E-01	347,29	165,42	6,89E-01	9152,82	635,81	9,54E-01
SPAC2G11.07c_b	10	390,72	243,88	393,44	315,17	7,00E-01	23215,68	8902,53	23648,00	8659,09	9,94E-01	433,53	249,00	8,58E-01	21494,30	8422,31	9,20E-01	315,17	333,14	9,35E-01	20156,10	8364,13	8,64E-01
SPAC2G11.09_a	5	1964,57	344,89	2646,74	286,03	8,48E-01	15500,21	23670,02	13777,25	22742,04	8,47E-01	2048,00	372,22	9,66E-01	11993,79	20955,43	6,59E-01	2149,82	257,78	9,72E-01	12416,75	20684,97	6,54E-01
SPAC2G11.10c_b	9	192,67	1951,00	183,55	2721,15	8,28E-01	5405,65	15286,81	5538,73	14164,58	9,47E-01	171,25	1807,78	9,52E-01	5759,38	12677,65	8,69E-01	194,01	2225,63	9,28E-01	5832,84	14066,74	9,56E-01

SPAC30.02c_a	5	359,54	171,25	522,76	159,79	7,46E-01	1379,57	5710,53	1910,85	5500,73	9,60E-01	498,00	179,77	7,29E-01	1937,53	5708,10	9,32E-01	474,41	183,55	7,49E-01	1652,00	5985,71	9,37E-01
SPAC30C2.08_a	5	477,71	288,01	675,59	357,05	5,46E-01	17438,64	1408,55	17198,56	1833,01	9,94E-01	458,25	382,68	7,48E-01	15500,21	1833,01	9,49E-01	564,18	407,31	4,91E-01	14263,10	1845,76	9,05E-01
SPAC30D11.02c_a	4	319,57	382,68	342,51	634,73	4,55E-01	147,58	17928,91	158,01	17928,91	1,00E+00	436,55	530,06	1,44E-01	154,14	17318,18	9,83E-01	354,59	541,19	4,29E-01	185,82	15076,35	9,15E-01
SPAC30D11.05_a	19	313,00	347,29	249,00	284,05	1,22E-01	2693,40	154,89	2474,30	171,70	9,58E-01	278,20	274,37	8,91E-02	2214,43	158,46	8,98E-01	362,04	337,79	4,46E-01	2662,66	157,24	9,94E-01
SPAC30D11.06c_b	8	885,29	286,03	1168,14	245,57	8,46E-01	7804,01	2463,14	6936,54	2215,94	8,90E-01	1067,48	270,60	8,83E-01	6251,56	1780,59	7,79E-01	1458,23	304,44	6,94E-01	6165,49	2625,03	8,39E-01
SPAC30D11.13_d	9	814,63	916,51	648,07	1251,98	8,09E-01	3527,90	7538,18	3940,66	7082,29	9,94E-01	749,61	724,08	1,34E-01	3660,31	5712,87	7,43E-01	809,00	1562,89	4,88E-01	5269,48	6165,49	9,37E-01
SPAC31A2.02_b	4	407,31	613,11	689,78	625,99	3,04E-01	4182,07	3311,69	4904,87	3605,01	5,83E-01	661,68	760,08	2,21E-01	3848,29	3627,85	9,86E-01	849,22	797,86	9,80E-02	4240,45	5261,95	2,73E-01
SPAC31A2.10_b	4	424,61	385,34	427,57	564,18	3,29E-01	1986,09	3902,01	2007,19	4482,23	8,65E-01	344,89	533,74	7,56E-01	1838,28	3350,13	8,01E-01	372,22	792,35	4,89E-01	2325,05	3983,99	8,83E-01
SPAC31G5.02_b	9	464,65	354,59	467,88	286,03	7,88E-01	6415,57	1734,33	7361,76	1621,29	9,21E-01	481,04	342,51	9,83E-01	6178,69	1547,12	9,55E-01	652,58	278,20	8,02E-01	7449,17	1880,39	8,86E-01
SPAC31G5.12c_a	15	572,05	442,64	487,75	474,41	7,25E-01	10314,40	5897,22	8648,23	7318,01	9,62E-01	661,68	508,46	5,19E-01	9207,92	5878,47	8,58E-01	1031,12	657,11	2,31E-01	11457,49	7039,69	7,50E-01
SPAC31G5.16c_a	5	257,78	433,53	257,78	404,50	9,11E-01	5613,53	9418,77	5554,48	7989,81	7,73E-01	184,82	530,06	9,57E-01	5790,60	8155,43	8,31E-01	225,97	982,29	5,74E-01	5412,30	10913,96	8,64E-01
SPAC31G5.17c_a	9	3444,31	222,86	1937,53	298,17	7,30E-01	23604,98	4517,42	22043,00	4884,13	9,67E-01	1937,53	210,84	7,18E-01	17157,20	5547,96	8,31E-01	4608,24	250,73	8,46E-01	18899,59	4751,45	8,68E-01
SPAC323.02c_b	6	996,00	3615,55	1897,65	2091,03	8,35E-01	24154,43	21284,71	24661,96	20388,83	9,47E-01	1552,09	2179,83	7,75E-01	24833,50	15137,56	6,43E-01	2179,83	4482,23	6,16E-01	21469,49	16394,38	3,23E-01
SPAC323.04_a	7	471,14	1136,20	552,56	2048,00	6,06E-01	14854,47	24154,43	15108,49	23987,58	9,95E-01	398,93	1686,71	7,73E-01	12714,17	26432,04	9,94E-01	526,39	2856,44	5,40E-01	11340,05	25006,23	8,87E-01
SPAC328.05_b	8	259,57	372,22	477,71	359,54	3,35E-01	12854,63	13647,91	11346,82	13370,25	4,98E-01	474,41	401,71	2,10E-01	11746,96	11931,12	7,40E-02	354,59	526,39	3,49E-01	11036,54	10624,60	3,24E-02
SPAC328.08c_a	11	298,17	288,01	342,51	604,67	3,03E-01	6051,62	12677,65	4653,76	11268,44	7,93E-01	337,79	657,11	3,29E-01	6141,86	12416,75	9,87E-01	324,03	401,71	2,17E-01	7567,85	10809,41	9,66E-01
SPAC328.09_a	11	1520,15	319,57	1082,39	328,56	7,91E-01	15856,46	5616,83	13647,93	4092,21	8,15E-01	1082,39	321,80	7,88E-01	11638,36	4955,96	7,28E-01	2091,03	308,69	8,19E-01	14282,40	6293,55	9,51E-01
SPAC328.10c_a	12	7858,29	1652,00	7538,18	1002,93	9,24E-01	24087,71	15359,09	24740,23	11987,49	8,77E-01	6382,92	1144,10	8,30E-01	19991,28	10503,14	5,59E-01	12416,75	2320,15	7,02E-01	20528,11	15154,46	7,49E-01
SPAC343.03_b	9	3258,52	6295,04	2998,45	5634,22	8,40E-01	8029,40	22383,85	6747,23	23829,82	9,95E-01	3373,43	5712,87	9,14E-01	8059,47	19554,45	8,93E-01	4182,07	13307,94	4,96E-01	8963,28	20689,65	9,71E-01
SPAC343.04c_a	5	284,05	2936,74	404,50	2977,74	9,69E-01	5220,60	8693,31	5955,47	6788,33	7,74E-01	310,83	3590,58	8,87E-01	6038,61	7860,81	9,97E-01	608,87	4240,45	7,52E-01	5220,60	9629,81	8,83E-01
SPAC343.08c_b	4	266,87	263,20	357,05	310,83	9,70E-02	2062,24	4359,66	2486,67	5518,27	7,18E-01	238,86	313,00	7,97E-01	2486,67	5367,37	7,35E-01	274,37	451,94	3,84E-01	2998,45	4513,40	7,30E-01
SPAC3A11.03_a	5	249,00	218,27	266,87	284,05	1,41E-01	1515,20	1978,24	1193,47	2225,63	9,54E-01	245,57	235,57	7,10E-01	1094,31	2418,67	9,90E-01	304,44	333,14	5,59E-02	1732,73	2521,38	4,93E-01
SPAC3A11.04_b	6	942,27	229,13	1360,57	237,21	7,79E-01	12245,81	1217,34	12503,12	1185,09	9,90E-01	1964,57	259,57	6,26E-01	11585,24	809,93	9,51E-01	1845,76	315,17	6,17E-01	10226,32	1251,55	9,02E-01
SPAC3A11.05c_a	5	2683,69	891,44	2048,00	1341,84	9,32E-01	2948,05	12503,12	2695,64	11993,79	9,60E-01	2288,20	1488,87	9,27E-01	3403,89	12416,75	9,80E-01	2916,45	1722,16	6,70E-01	3484,82	11665,82	9,83E-01
SPAC3A11.06_i	6	968,76	2876,30	724,08	1530,73	5,23E-01	2533,90	2895,43	2806,40	2573,44	9,19E-01	1045,52	2336,28	8,59E-01	3370,01	3418,80	6,51E-02	1136,20	3396,89	8,38E-01	3403,33	3356,33	6,76E-02
SPAC3A11.09_a	10	308,69	451,94	385,34	382,68	9,64E-01	3025,28	2317,05	4062,26	2631,20	4,87E-01	317,37	584,07	6,88E-01	4894,02	3216,14	2,68E-01	380,04	613,11	4,85E-01	4003,55	3335,74	1,77E-01
SPAC3A11.12c_a	7	765,36	280,14	968,76	330,84	7,81E-01	6989,43	3768,93	7593,09	4546,45	7,85E-01	989,12	330,84	7,69E-01	9184,92	4616,68	6,41E-01	996,00	380,04	7,14E-01	6892,41	3861,96	9,99E-01
SPAC3A11.13_a	5	781,44	754,83	1629,26	879,17	3,25E-01	19215,73	6503,41	17928,91	7726,80	9,97E-01	1552,09	996,00	2,11E-01	14664,09	9060,27	8,99E-01	2062,24	1105,13	2,31E-01	14263,10	6412,87	7,68E-01
SPAC3A11.14c_c	5	372,22	685,02	1089,92	1305,15	7,20E-02	1067,48	16158,44	982,29	16497,96	9,92E-01	929,30	1573,76	1,81E-01	1184,45	15393,14	9,78E-01	504,95	2556,58	4,36E-01	1370,04	14868,79	9,66E-01
SPAC3A12.02_a	4	1964,57	390,72	1640,59	797,86	9,67E-01	7496,35	1024,00	6499,10	955,43	9,12E-01	1924,14	613,11	9,37E-01	5647,11	1184,45	8,50E-01	2702,35	592,22	7,55E-01	6368,69	1332,57	9,30E-01

SPAC3A12.14_a	5	765,36	1618,00	734,19	1360,57	8,11E-01	22612,69	6544,09	21587,46	5960,04	9,49E-01	837,53	1251,98	7,86E-01	22040,93	5666,39	9,55E-01	996,00	1795,29	7,60E-01	20582,39	5494,08	9,02E-01
SPAC3A12.18_a	4	1305,15	526,39	1209,34	501,46	9,19E-01	23010,79	20347,63	23432,58	20262,58	9,43E-01	922,88	526,39	7,04E-01	23259,80	21816,39	6,28E-01	916,51	814,63	9,10E-01	17238,65	18479,08	1,21E-01
SPAC3F10.06c_a	11	1458,23	831,75	1016,93	754,83	5,25E-01	10223,00	21450,54	8121,19	23452,33	9,96E-01	1269,46	867,07	8,56E-01	8376,64	24267,30	9,65E-01	1629,26	1458,23	3,44E-01	10270,17	16497,10	7,39E-01
SPAC3F10.09_b	11	455,09	1217,75	689,78	861,08	8,90E-01	5184,54	10502,08	5673,41	8284,49	7,98E-01	1217,75	916,51	6,30E-01	4705,07	7974,26	6,78E-01	831,75	1398,83	6,17E-01	4938,99	9456,51	8,70E-01
SPAC3F10.16c_a	4	216,77	382,68	205,07	709,18	6,13E-01	10003,29	4837,35	9593,46	5442,30	9,79E-01	221,32	1351,18	4,84E-01	8344,22	4870,99	8,18E-01	280,14	820,30	4,69E-01	9760,49	4870,99	9,79E-01
SPAC3F10.17_a	5	259,57	186,11	254,23	227,54	6,90E-01	3761,45	9059,87	3354,60	8492,44	9,07E-01	221,32	191,34	7,18E-01	3548,74	6924,90	7,45E-01	250,73	222,86	7,56E-01	3720,88	9022,91	9,93E-01
SPAC3G6.02_a	4	304,44	216,77	477,71	237,21	5,28E-01	21769,19	3784,83	20882,40	3338,00	9,62E-01	474,41	216,77	5,96E-01	22381,20	3487,57	9,91E-01	407,31	265,03	4,61E-01	20311,37	3509,50	9,50E-01
SPAC3G6.03c_a	5	491,14	213,78	515,56	415,87	5,23E-01	12189,43	19755,96	12898,94	20171,07	9,24E-01	680,29	317,37	5,87E-01	9997,29	19755,96	8,75E-01	592,22	388,02	5,08E-01	10404,68	21618,82	9,96E-01
SPAC3G6.03c_c	11	494,56	572,05	467,88	515,56	4,57E-01	12189,43	15044,27	12898,94	13234,55	7,39E-01	508,46	471,14	4,18E-01	9997,29	12040,00	2,77E-01	754,83	760,08	2,87E-02	10404,68	13392,09	4,93E-01
SPAC3G6.06c_a	17	233,94	537,45	238,86	415,87	7,71E-01	1930,37	15044,27	1927,33	13234,55	9,26E-01	261,38	481,04	9,45E-01	2003,19	12040,00	8,75E-01	265,03	709,18	7,42E-01	2187,50	13392,09	9,43E-01
SPAC3G9.06_b	6	588,13	196,72	739,29	181,02	8,61E-01	4904,87	1896,68	5480,15	1880,72	9,16E-01	867,07	233,94	7,12E-01	4211,15	1800,48	8,57E-01	1243,34	286,03	5,46E-01	4359,66	1730,79	8,75E-01
SPAC3G9.10c_b	4	174,85	467,88	172,45	709,18	7,34E-01	2047,27	3902,01	2052,01	4299,64	9,03E-01	163,14	576,03	8,67E-01	1856,97	3213,66	7,39E-01	184,82	942,27	6,11E-01	2367,40	3326,99	9,14E-01
SPAC3G9.13c_a	5	344,89	195,36	396,18	170,07	9,32E-01	5327,79	2221,35	5615,05	2191,57	9,61E-01	504,95	212,31	6,44E-01	5292,15	1765,90	9,26E-01	584,07	196,72	6,21E-01	5336,20	1922,20	9,56E-01
SPAC3H5.07_a	12	319,57	377,41	259,57	415,87	9,09E-01	17975,64	5319,87	17755,12	5178,13	9,86E-01	235,57	448,82	9,60E-01	11163,74	4688,85	6,53E-01	388,02	501,46	2,70E-01	14317,35	5227,84	8,32E-01
SPAC3H8.03_a	4	401,71	261,38	421,68	225,97	9,55E-01	5172,56	15610,49	5004,46	15769,20	1,00E+00	544,96	245,57	7,37E-01	4882,28	10104,55	6,69E-01	484,38	385,34	3,52E-01	5271,08	12881,08	8,57E-01
SPAC3H8.10_b	7	396,18	393,44	481,04	333,14	8,83E-01	19619,49	4280,37	19349,38	4422,27	9,96E-01	458,25	630,35	2,25E-01	19483,97	4494,77	9,97E-01	572,05	455,09	1,79E-01	16497,96	5424,39	9,26E-01
SPAC458.02c_a	4	179,77	296,11	162,02	413,00	7,54E-01	10122,57	18820,27	10470,35	18305,63	9,90E-01	144,01	393,44	8,44E-01	8277,84	18951,18	9,12E-01	182,28	464,65	6,32E-01	7883,86	16384,00	7,38E-01
SPAC458.02c_b	5	124,50	190,02	110,66	153,28	5,84E-01	10122,57	9823,80	10470,35	10196,35	2,18E-01	150,12	131,60	6,78E-01	8277,84	8859,71	5,02E-02	122,79	166,57	7,80E-01	7883,86	7599,49	8,43E-03
SPAC458.07_c	4	190,02	116,97	254,23	138,14	5,97E-01	1858,60	9823,80	2368,90	10196,35	9,44E-01	227,54	109,14	8,51E-01	2721,15	8859,71	9,93E-01	254,23	121,94	6,92E-01	2062,24	7599,49	8,54E-01
SPAC4A8.10_a	6	298,17	178,53	359,54	202,25	7,09E-01	2983,55	1858,60	3016,64	2368,90	7,16E-01	205,07	222,86	7,26E-01	2680,04	2521,38	7,82E-01	238,86	249,00	9,34E-01	1826,68	1807,78	3,95E-01
SPAC4C5.02c_a	4	272,48	292,04	359,54	330,84	6,84E-02	6338,83	2830,13	5955,47	2920,72	9,55E-01	372,22	257,78	6,30E-01	5367,37	2714,34	8,28E-01	310,83	222,86	7,65E-01	5595,30	1692,04	7,54E-01
SPAC4D7.02c_a	6	1002,93	252,48	929,30	344,89	9,86E-01	12240,47	4737,79	11302,93	4608,24	9,25E-01	1060,11	252,48	9,63E-01	14154,97	4451,27	9,07E-01	1217,75	252,48	8,77E-01	14482,93	4608,24	8,80E-01
SPAC4D7.05_b	15	643,59	1112,82	814,63	942,27	9,99E-01	17318,18	12619,65	17682,08	12190,41	9,94E-01	744,43	1097,50	8,98E-01	16961,78	15812,94	6,17E-01	916,51	2062,24	4,28E-01	17318,18	16508,58	5,00E-01
SPAC4D7.06c_a	7	362,04	634,73	568,10	809,00	4,06E-01	8248,98	15825,90	9674,69	17079,76	8,24E-01	471,14	617,37	7,95E-01	9026,81	16728,26	8,91E-01	487,75	897,64	5,13E-01	8480,89	15825,90	9,84E-01
SPAC4D7.09_a	4	288,01	250,73	245,57	541,19	4,93E-01	7391,88	6793,79	7949,17	9809,75	2,09E-01	224,41	413,00	6,59E-01	6073,84	7696,57	8,33E-01	294,07	442,64	3,25E-01	6850,06	6472,02	3,47E-01
SPAC4D7.09_b	5	382,68	250,73	364,56	292,04	8,92E-01	7391,88	6627,97	7949,17	7261,05	3,67E-01	398,93	216,77	9,44E-01	6073,84	5563,06	1,22E-01	418,77	282,09	7,57E-01	6850,06	5761,65	4,01E-01
SPAC4D7.11_b	4	903,89	292,04	1176,27	304,44	8,14E-01	10660,59	6627,97	10586,95	7261,05	9,25E-01	1105,13	413,00	7,61E-01	10226,32	5563,06	8,31E-01	1097,50	451,94	7,29E-01	10660,59	5761,65	9,04E-01
SPAC4F10.03c_a	6	250,73	694,58	294,07	1067,48	6,87E-01	15376,14	10015,87	15024,79	9946,68	9,60E-01	288,01	1024,00	7,11E-01	15689,83	9345,14	9,70E-01	390,72	1120,56	5,76E-01	17419,30	10085,54	8,38E-01
SPAC4F10.07c_a	10	739,29	317,37	1038,29	227,54	8,40E-01	10809,41	14727,84	9946,68	14682,19	8,96E-01	948,83	265,03	8,63E-01	12077,21	15771,58	7,10E-01	1243,34	278,20	7,02E-01	9877,98	17974,61	8,21E-01
SPAC4F10.13c_a	4	2702,35	560,28	3125,78	831,75	8,45E-01	11548,68	10809,41	11898,44	10513,82	9,76E-01	2225,63	843,36	9,46E-01	10483,08	12854,63	7,31E-01	2164,77	867,07	9,35E-01	10302,36	10441,20	1,65E-01

SPAC4F10.14c_a	6	3040,30	2916,45	2876,30	2469,49	2,87E-01	43775,67	12092,17	42483,63	12078,11	9,79E-01	3061,45	2288,20	5,19E-01	38917,68	11856,81	9,14E-01	5752,61	2896,31	4,46E-01	39585,20	10924,95	9,12E-01
SPAC4F10.18_b	6	254,23	2210,26	306,55	2721,15	8,73E-01	3852,40	41843,17	4257,73	40848,07	9,92E-01	247,28	3236,01	8,02E-01	4219,82	40380,38	9,85E-01	333,14	6338,83	5,74E-01	3968,11	39072,67	9,64E-01
SPAC4F10.19c_a	4	474,41	265,03	803,41	237,21	6,67E-01	5832,91	3327,40	5518,27	3607,04	9,92E-01	648,07	242,19	7,73E-01	4904,87	3464,89	8,10E-01	962,07	282,09	5,52E-01	5442,30	3124,36	8,78E-01
SPAC4F10.20_b	7	843,36	413,00	2149,82	613,11	4,45E-01	6038,61	5480,15	18432,96	5595,30	4,33E-01	1323,37	867,07	2,75E-01	21173,91	6038,61	4,09E-01	837,53	1251,98	2,98E-01	8964,45	5914,33	3,92E-01
SPAC4F10.20_c	5	282,09	770,69	792,35	1323,37	2,79E-01	6038,61	4672,57	18432,96	13124,73	6,27E-02	471,14	962,07	6,38E-01	21173,91	20452,65	2,49E-03	442,64	891,44	7,13E-01	8964,45	8192,00	5,45E-02
SPAC4F8.01_a	7	182,28	263,20	153,28	584,07	5,74E-01	3428,52	4672,57	4015,65	13124,73	4,29E-01	165,42	548,75	5,64E-01	4638,28	20452,65	3,96E-01	183,55	340,14	7,01E-01	4451,65	8192,00	3,68E-01
SPAC4F8.03_a	9	190,02	172,45	205,07	172,45	7,24E-01	3267,71	3637,29	3091,92	4033,88	8,48E-01	230,72	171,25	5,89E-01	3090,62	4459,83	6,94E-01	333,14	174,85	4,57E-01	3531,98	3856,12	4,29E-01
SPAC4F8.14c_a	7	544,96	162,02	596,34	206,50	8,77E-01	24193,13	2362,40	24000,53	2518,69	9,99E-01	652,58	265,03	7,36E-01	22163,77	2568,33	9,56E-01	942,27	222,86	6,31E-01	20019,83	2750,67	9,04E-01
SPAC4G8.02c_a	6	188,71	498,00	268,73	515,56	8,28E-01	4067,71	23780,57	5996,90	23793,69	9,48E-01	207,94	522,76	9,30E-01	6517,03	23363,98	9,45E-01	232,32	843,36	6,27E-01	5792,62	19647,14	9,29E-01
SPAC4G8.02c_b	4	187,40	181,02	247,28	213,78	1,13E-01	4067,71	2721,15	5996,90	5184,54	1,08E-01	157,59	198,09	7,85E-01	6517,03	4640,29	1,99E-01	221,32	237,21	3,43E-02	5792,62	3516,68	4,41E-01
SPAC4G8.07c_a	6	148,06	150,12	166,57	178,53	6,08E-02	7604,40	2721,15	8159,12	5184,54	6,50E-01	164,28	148,06	4,78E-01	8411,29	4640,29	7,02E-01	148,06	187,40	4,44E-01	9046,18	3516,68	7,90E-01
SPAC4G8.07c_c	4	349,71	195,36	484,38	186,11	7,45E-01	7604,40	7551,60	8159,12	7591,22	4,07E-01	404,50	170,07	9,26E-01	8411,29	7884,88	1,64E-01	680,29	151,17	6,55E-01	9046,18	9556,85	2,15E-02
SPAC4G8.08_b	5	140,07	292,04	167,73	308,69	8,51E-01	2856,44	7551,60	3565,78	7591,22	9,15E-01	159,79	467,88	6,27E-01	3350,13	7884,88	9,11E-01	122,79	508,46	6,78E-01	3541,14	9556,85	7,58E-01
SPAC4G9.13c_a	4	218,27	124,50	233,94	156,50	7,33E-01	1310,54	2936,74	1710,18	3326,99	7,63E-01	199,47	136,24	9,56E-01	1884,39	2856,44	8,19E-01	272,48	142,02	6,99E-01	1628,07	2977,74	8,81E-01
SPAC4G9.14_c	4	306,55	210,84	247,28	199,47	5,77E-01	6179,69	1233,90	5063,60	1671,82	9,20E-01	230,72	276,28	9,31E-01	4396,54	1654,91	8,32E-01	259,57	328,56	6,10E-01	4909,34	1536,62	8,86E-01
SPAC4G9.17c_a	5	213,78	290,02	286,03	261,38	6,41E-01	5634,22	5167,03	6793,79	4659,56	7,93E-01	212,31	224,41	4,76E-01	5556,65	3595,37	4,99E-01	274,37	282,09	5,63E-01	5404,70	4408,08	4,64E-01
SPAC4G9.20c_a	14	1746,20	199,47	1458,23	304,44	9,33E-01	31878,35	5367,37	27933,82	6208,38	9,36E-01	1595,73	280,14	9,76E-01	22828,62	4973,34	7,95E-01	2091,03	237,21	8,89E-01	27969,18	5792,62	9,29E-01
SPAC4H3.07c_c	5	689,78	1360,57	630,35	1105,13	7,38E-01	17648,04	29239,37	18551,64	25114,85	8,31E-01	522,76	1192,69	7,58E-01	16006,25	20642,20	4,98E-01	820,30	1833,01	6,69E-01	16485,35	25415,37	7,66E-01
SPAC4H3.09_a	6	929,30	584,07	989,12	512,00	9,85E-01	22688,51	17161,76	24952,78	17286,61	8,24E-01	675,59	576,03	5,42E-01	23061,95	16836,27	9,96E-01	903,89	786,88	6,75E-01	23574,63	17172,74	9,25E-01
SPAC56E4.04c_a	28	2320,15	689,78	2304,12	657,11	9,85E-01	28156,05	22809,32	28530,32	23634,77	8,84E-01	1686,71	604,67	7,49E-01	24111,56	20978,76	4,43E-01	1269,46	982,29	6,92E-01	18222,36	23163,81	3,19E-01
SPAC56F8.05c_b	5	261,38	2225,63	306,55	2149,82	9,92E-01	5201,36	29847,03	5800,38	30108,91	9,82E-01	294,07	1499,22	7,92E-01	4452,77	27027,91	9,25E-01	298,17	1217,75	6,98E-01	4251,81	21243,60	7,80E-01
SPAC56F8.05c_d	7	831,75	261,38	922,88	288,01	9,03E-01	5184,54	3627,45	5792,62	4476,02	5,49E-01	568,10	284,05	7,42E-01	4451,27	3269,47	6,33E-01	675,59	304,44	8,83E-01	4240,45	3113,78	5,27E-01
SPAC56F8.08_a	5	666,29	600,49	837,53	885,29	3,03E-02	14462,21	3615,55	14263,10	4482,23	9,68E-01	689,78	670,92	3,04E-01	13969,57	3258,52	9,61E-01	1584,71	1060,11	1,21E-01	13307,94	3104,19	9,21E-01
SPAC56F8.11_a	7	792,35	541,19	885,29	670,92	5,70E-01	11897,50	13587,57	12455,17	13034,07	9,98E-01	891,44	916,51	2,01E-01	10067,09	12330,98	3,89E-01	831,75	1562,89	3,04E-01	9999,46	12854,63	5,11E-01
SPAC57A10.03_c	9	183,55	652,58	230,72	689,78	9,09E-01	4374,90	11538,02	4363,78	10687,73	9,36E-01	190,02	922,88	7,81E-01	3534,12	8699,34	7,17E-01	219,79	849,22	7,95E-01	5015,79	10244,73	9,48E-01
SPAC57A10.09c_a	11	1428,22	163,14	1217,75	172,45	9,14E-01	9046,43	3450,18	9538,83	3312,20	9,70E-01	1652,00	186,11	9,10E-01	9201,82	2693,51	9,51E-01	1652,00	203,66	9,03E-01	8291,49	4475,63	9,72E-01
SPAC57A10.14_a	10	592,22	1184,45	617,37	1002,93	8,45E-01	15944,70	8588,18	14098,89	8752,41	8,70E-01	552,56	1184,45	9,68E-01	12717,42	9368,52	7,91E-01	809,00	1675,06	5,70E-01	11607,55	9040,09	6,67E-01
SPAC57A7.12_a	5	170,07	548,75	209,38	526,39	9,76E-01	22742,07	14415,30	24316,48	13890,36	9,44E-01	154,34	639,15	9,14E-01	20690,78	12629,22	7,72E-01	203,66	1045,52	6,24E-01	22480,81	9830,75	7,79E-01
SPAC589.05c_a	6	522,76	205,07	494,56	178,53	9,14E-01	4615,15	23956,56	4193,30	25599,31	9,70E-01	749,61	176,07	7,91E-01	3894,97	22190,65	9,34E-01	621,67	202,25	8,72E-01	3638,16	22919,42	9,48E-01
SPAC589.06c_a	5	572,05	458,25	1200,98	430,54	5,21E-01	17928,91	4546,80	18951,18	3489,68	9,99E-01	719,08	608,87	2,01E-01	18432,96	3136,01	9,68E-01	544,96	680,29	3,85E-01	14362,31	3390,17	8,11E-01

SPAC589.07c_e	7	661,68	445,72	826,00	910,17	1,13E-01	2702,35	17438,64	2936,74	18179,19	9,68E-01	515,56	552,56	8,74E-01	2304,12	18820,27	9,69E-01	474,41	544,96	7,36E-01	2288,20	12245,81	7,82E-01
SPAC589.11_a	12	321,80	541,19	240,52	729,11	8,61E-01	945,98	2628,46	690,43	2628,46	9,30E-01	404,50	464,65	9,81E-01	960,73	1871,53	7,35E-01	388,02	445,72	9,09E-01	1006,88	1992,00	7,96E-01
SPAC589.12_a	7	744,43	292,04	657,11	210,84	8,16E-01	10906,38	864,83	11141,66	738,07	9,95E-01	760,08	377,41	8,80E-01	8915,87	837,19	8,90E-01	1016,93	396,18	6,72E-01	9113,28	1015,01	9,10E-01
SPAC5D6.04_c	5	224,41	744,43	224,41	657,11	9,09E-01	12776,67	11510,90	11851,37	11774,90	6,54E-01	183,55	797,86	9,89E-01	12851,36	8891,56	6,03E-01	215,27	831,75	9,32E-01	11684,23	9631,68	3,43E-01
SPAC5D6.05_c	4	188,71	200,85	310,83	209,38	3,29E-01	1770,57	12495,89	2628,46	10967,73	9,65E-01	237,21	190,02	5,20E-01	2048,00	11812,58	9,80E-01	282,09	240,52	9,16E-02	1884,54	11616,18	9,63E-01
SPAC607.03c_a	6	372,22	192,67	377,41	455,09	3,05E-01	5753,03	1629,26	5802,27	2628,46	8,59E-01	288,01	367,09	6,91E-01	6439,86	2164,77	8,56E-01	310,83	230,72	9,16E-01	7345,80	1746,20	8,29E-01
SPAC630.03_c	4	600,49	209,38	621,67	256,00	9,11E-01	20502,27	3952,75	20832,77	5094,73	9,54E-01	648,07	219,79	9,29E-01	20659,03	4716,28	9,72E-01	935,76	333,14	5,88E-01	17811,89	4334,10	9,24E-01
SPAC630.11_d	5	418,77	481,04	404,50	604,67	6,54E-01	10414,38	18748,80	12508,47	19402,06	8,23E-01	247,28	652,58	1,00E+00	12935,68	19423,70	7,91E-01	326,29	1296,13	5,35E-01	13742,77	16940,10	8,80E-01
SPAC630.14c_a	11	1629,26	290,02	1314,23	294,07	8,70E-01	16763,53	9453,59	14914,68	10838,22	9,61E-01	1408,55	288,01	9,10E-01	15815,00	11048,55	9,48E-01	2134,97	390,72	8,09E-01	15555,74	12240,85	8,62E-01
SPAC630.14c_b	17	1038,29	1488,87	1243,34	1243,34	9,37E-01	16728,26	19504,90	14868,79	17541,14	4,26E-01	1192,69	1226,22	8,33E-01	15825,90	18731,84	7,17E-01	1251,98	2091,03	4,82E-01	15608,02	17392,28	4,31E-01
SPAC631.01c_b	7	357,05	982,29	288,01	1251,98	8,77E-01	21579,37	19483,97	20924,61	17559,94	5,82E-01	315,17	1438,15	7,78E-01	20155,57	18690,27	4,77E-01	354,59	1226,22	8,43E-01	18149,80	17438,64	1,32E-01
SPAC644.09_a	15	491,14	313,00	544,96	326,29	8,34E-01	2608,03	20558,44	3133,91	19653,94	9,89E-01	556,41	347,29	7,52E-01	2966,96	19838,14	9,90E-01	617,37	374,81	5,96E-01	2493,26	17480,74	9,04E-01
SPAC644.09_b	4	831,75	382,68	803,41	430,54	9,76E-01	2608,03	2659,54	3133,91	2946,48	5,27E-02	861,08	451,94	8,86E-01	2966,96	2878,58	2,99E-02	792,35	501,46	8,96E-01	2493,26	2717,77	8,29E-01
SPAC644.15_a	4	347,29	652,58	286,03	786,88	9,12E-01	10571,02	2659,54	11251,99	2946,48	9,40E-01	199,47	754,83	9,49E-01	8459,17	2878,58	8,63E-01	410,15	643,59	9,01E-01	9930,64	2717,77	9,62E-01
SPAC664.02c_b	5	235,57	237,21	243,88	183,55	5,31E-01	5741,94	8702,02	5417,00	9299,11	9,61E-01	216,77	215,27	2,96E-03	5144,82	6584,22	4,96E-01	292,04	290,02	5,65E-04	5822,14	7199,33	7,06E-01
SPAC664.04c_a	6	268,73	250,73	250,73	261,38	7,59E-01	30243,86	5511,99	31447,79	5552,67	9,75E-01	227,54	188,71	1,37E-01	25570,03	5237,12	8,91E-01	268,73	221,32	6,21E-01	27066,46	5774,66	9,37E-01
SPAC664.06_a	8	1530,73	254,23	1217,75	276,28	8,71E-01	4318,82	28137,48	4437,91	28425,48	9,91E-01	1209,34	274,37	8,67E-01	4451,72	25031,37	9,33E-01	1287,18	233,94	8,88E-01	6146,74	25108,50	9,72E-01
SPAC688.02c_a	4	1323,37	1296,13	1045,52	1136,20	4,37E-02	2260,74	3804,03	2581,77	4211,67	7,76E-01	1260,69	1217,75	1,09E-01	2473,77	4100,30	8,41E-01	1234,75	1160,07	1,06E-01	2459,07	5231,29	6,59E-01
SPAC688.03c_a	5	219,79	1074,91	222,86	1074,91	9,98E-01	653,56	2511,26	889,48	2552,04	9,22E-01	298,17	1260,69	8,56E-01	1469,89	2464,04	7,50E-01	266,87	1606,83	7,51E-01	1226,17	2360,65	8,64E-01
SPAC694.04c_a	14	754,83	238,86	760,08	198,09	9,67E-01	6013,51	714,62	7892,37	739,78	8,50E-01	982,29	296,11	7,72E-01	6480,51	1275,92	9,03E-01	1488,87	272,48	6,20E-01	6608,01	949,51	9,25E-01
SPAC6B12.08_a	5	270,60	580,04	276,28	625,99	9,22E-01	1994,72	5841,34	1715,45	7577,01	8,55E-01	266,87	734,19	8,14E-01	1686,78	5974,45	9,79E-01	415,87	1389,16	4,49E-01	1912,39	6711,78	9,10E-01
SPAC6B12.13_a	9	580,04	195,36	709,18	278,20	7,49E-01	7033,37	1621,78	5556,65	1571,77	8,41E-01	576,03	294,07	8,61E-01	5556,65	1490,45	8,34E-01	786,88	315,17	6,45E-01	6653,97	1703,17	9,71E-01
SPAC6B12.15_a	8	4973,34	544,96	3929,15	744,43	8,91E-01	46685,39	6080,61	46702,34	5752,61	9,96E-01	3902,01	843,36	8,99E-01	42601,36	5914,33	9,45E-01	6165,49	849,22	8,49E-01	43048,63	6427,31	9,57E-01
SPAC6B12.15_b	12	2120,22	4240,45	2033,85	3236,01	6,98E-01	46685,39	45194,16	46702,34	44307,62	7,87E-01	1782,89	3717,20	7,93E-01	42601,36	43842,75	1,07E-01	2721,15	6608,01	5,72E-01	43048,63	42605,27	5,71E-02
SPAC6C3.04_a	33	1897,65	1820,35	1448,15	1845,76	4,05E-01	15423,74	45194,16	15566,86	44307,62	9,87E-01	1278,29	1770,57	3,11E-01	14596,00	43842,75	9,63E-01	1833,01	2628,46	4,50E-01	15087,57	42605,27	9,49E-01
SPAC6C3.08_a	5	172,45	1168,14	166,57	1024,00	9,20E-01	1132,20	14338,59	1223,45	14785,17	9,80E-01	127,12	942,27	8,53E-01	1271,45	13722,44	9,81E-01	147,03	1379,57	9,17E-01	1163,42	14702,86	9,85E-01
SPAC6C3.09_c	4	803,41	151,17	803,41	178,53	9,79E-01	4414,87	947,75	4454,41	987,37	9,89E-01	719,08	157,59	9,36E-01	3994,77	1131,29	9,63E-01	685,02	191,34	9,33E-01	3392,74	963,26	8,34E-01
SPAC6F12.07_a	13	867,07	643,59	897,64	749,61	6,61E-01	13460,93	3732,02	13476,68	4223,87	9,73E-01	922,88	467,88	8,35E-01	11898,19	3582,62	9,06E-01	1499,22	948,83	2,55E-01	11757,89	3373,58	8,87E-01
SPAC6F12.11c_a	4	596,34	689,78	648,07	652,58	8,91E-01	2746,32	12649,92	2717,83	13684,38	9,52E-01	576,03	831,75	6,99E-01	4582,68	10988,91	9,89E-01	512,00	1217,75	5,97E-01	3488,09	11480,16	9,76E-01
SPAC6F12.13c_a	9	313,00	448,82	298,17	364,56	5,80E-01	9359,39	2660,42	11013,29	2670,61	8,91E-01	398,93	333,14	8,62E-01	9294,78	4298,00	8,68E-01	560,28	491,14	1,98E-01	7785,58	3294,07	9,18E-01

SPAC6F12.14_a	14	357,05	242,19	357,05	276,28	8,31E-01	779,80	8190,04	711,58	9560,38	9,20E-01	464,65	310,83	4,56E-01	872,23	7904,57	9,87E-01	471,14	564,18	9,83E-02	917,26	7599,52	9,68E-01
SPAC6F6.03c_a	17	996,00	306,55	1120,56	337,79	8,95E-01	5284,22	714,15	5407,22	593,83	1,00E+00	855,13	380,04	9,43E-01	5371,00	709,68	9,91E-01	1128,35	451,94	8,01E-01	5925,34	782,33	9,27E-01
SPAC6F6.08c_a	6	781,44	781,44	685,02	916,51	8,83E-01	18988,15	4959,07	16817,60	5157,15	9,24E-01	685,02	803,41	5,94E-01	16623,71	4912,46	9,07E-01	639,15	1323,37	6,18E-01	17269,73	5343,00	9,49E-01
SPAC6F6.19_a	7	1031,12	661,68	1152,06	487,75	9,51E-01	1959,60	18973,74	2375,07	15602,72	9,03E-01	837,53	544,96	5,78E-01	2815,28	15932,36	9,28E-01	724,08	786,88	6,75E-01	2755,19	16632,31	9,50E-01
SPAC6G10.04c_a	7	333,14	739,29	396,18	724,08	9,35E-01	11007,63	1639,26	11686,60	2040,39	9,43E-01	458,25	885,29	6,91E-01	11754,29	2169,48	9,33E-01	319,57	760,08	9,91E-01	11120,17	2202,01	9,63E-01
SPAC6G9.01c_b	17	657,11	326,29	1176,27	330,84	6,22E-01	1351,18	10183,60	2368,90	10333,95	9,31E-01	1251,98	372,22	5,66E-01	2538,92	11969,47	8,39E-01	1200,98	448,82	5,03E-01	2033,85	11191,20	9,06E-01
SPAC6G9.07c_a	7	537,45	643,59	491,14	1217,75	5,47E-01	10985,92	1323,37	8680,92	2164,77	9,12E-01	385,34	1323,37	6,32E-01	10163,08	2241,11	9,95E-01	548,75	1278,29	4,73E-01	10680,01	1675,06	9,98E-01
SPAC6G9.07c_b	5	268,73	380,04	385,34	377,41	4,14E-01	10960,30	11628,55	8659,09	9424,64	4,73E-02	296,11	421,68	7,21E-01	10155,69	11232,11	4,43E-01	278,20	533,74	6,18E-01	10660,59	12383,25	8,28E-01
SPAC6G9.13c_a	4	166,57	222,86	216,77	357,05	3,47E-01	439,54	11665,82	689,45	9410,14	9,01E-01	212,31	268,73	3,69E-01	581,23	11268,44	9,88E-01	196,72	306,55	4,54E-01	1019,56	12416,75	9,41E-01
SPAC6G9.16c_b	6	494,56	167,73	467,88	163,14	9,51E-01	863,29	421,36	755,19	665,30	7,92E-01	709,18	148,06	7,92E-01	1038,28	644,39	5,71E-01	760,08	192,67	7,01E-01	1227,21	991,76	2,03E-01
SPAC732.01_e	11	240,52	552,56	268,73	415,87	7,83E-01	12139,31	929,42	14022,04	653,52	9,35E-01	288,01	464,65	9,21E-01	14715,45	844,40	9,02E-01	266,87	781,44	7,13E-01	14385,33	1280,81	8,94E-01
SPAC7D4.02c_a	7	541,19	225,97	560,28	237,21	9,53E-01	12227,57	11024,71	10656,01	11986,25	7,66E-01	458,25	218,27	8,40E-01	8912,57	11078,42	3,19E-01	588,13	232,32	9,21E-01	10080,57	11747,69	5,60E-01
SPAC7D4.02c_b	4	1152,06	576,03	1332,57	390,72	9,97E-01	12245,81	11518,54	10660,59	10313,74	7,42E-02	1964,57	382,68	7,48E-01	8902,53	9003,68	1,53E-02	2336,28	592,22	5,80E-01	10085,54	10299,36	4,68E-02
SPAC7D4.10_a	11	556,41	996,00	552,56	1314,23	7,55E-01	17185,19	11505,21	16622,89	10297,45	8,54E-01	477,71	1562,89	7,17E-01	15832,05	9026,81	7,08E-01	390,72	2610,30	5,88E-01	13829,97	10297,45	5,66E-01
SPAC7D4.10_b	6	461,44	504,95	461,44	519,15	8,62E-01	17185,19	15305,45	16622,89	15852,15	9,95E-01	455,09	548,75	7,52E-01	15832,05	15463,24	5,96E-01	501,46	481,04	7,69E-01	13829,97	12848,30	1,11E-01
SPAC821.05_b	7	685,02	552,56	867,07	396,18	9,63E-01	18820,27	15305,45	20452,65	15852,15	7,43E-01	729,11	461,44	8,89E-01	17682,08	15463,24	8,35E-01	955,43	584,07	5,24E-01	17682,08	12848,30	6,09E-01
SPAC821.06_c	4	181,02	592,22	206,50	729,11	8,30E-01	19787,45	16612,71	18618,55	18561,17	8,29E-01	229,13	675,59	8,49E-01	15318,53	16270,83	2,84E-01	227,54	916,51	6,89E-01	17375,94	15935,98	4,69E-01
SPAC821.12_a	10	374,81	195,36	533,74	199,47	7,09E-01	14362,31	19195,23	13216,02	17698,94	7,27E-01	501,46	206,50	7,28E-01	12677,65	14523,67	3,44E-01	592,22	224,41	6,08E-01	13216,02	18322,30	8,01E-01
SPAC823.14_b	9	494,56	362,04	481,04	464,65	5,73E-01	12666,27	13777,25	11611,74	12245,81	1,81E-01	467,88	477,71	5,72E-01	10120,31	12245,81	2,31E-01	491,14	661,68	3,04E-01	10136,81	12590,08	3,02E-01
SPAC824.07_a	5	739,29	464,65	809,00	424,61	9,56E-01	4622,29	11128,42	5837,56	11009,72	9,07E-01	855,13	467,88	8,25E-01	7469,16	9532,95	8,71E-01	935,76	515,56	6,71E-01	6105,48	10699,12	9,07E-01
SPAC824.09c_a	5	2210,26	820,30	2538,92	689,78	9,40E-01	18677,11	4621,03	18251,06	6379,87	9,49E-01	2503,97	760,08	9,26E-01	17456,16	7820,12	9,18E-01	3848,29	873,10	6,58E-01	16440,81	6125,29	9,70E-01
SPAC890.03_c	7	765,36	1629,26	776,05	1341,84	8,14E-01	13943,85	17852,44	11274,71	16869,44	6,46E-01	699,41	1910,85	8,98E-01	12268,29	17822,07	8,25E-01	739,29	3125,78	6,21E-01	12249,68	18041,00	8,49E-01
SPAC890.04c_a	14	2817,11	608,87	3717,20	729,11	8,09E-01	13577,24	13398,52	13797,67	11355,19	5,34E-01	3061,45	613,11	9,47E-01	12347,55	12679,73	3,55E-02	3468,27	867,07	8,15E-01	13452,96	12476,26	4,02E-01
SPAC890.07c_f	7	1009,90	2592,27	891,44	2336,28	8,77E-01	18269,50	12136,69	17931,47	12649,78	9,85E-01	1031,12	2048,00	8,07E-01	12387,96	11412,31	3,99E-01	1251,98	3281,18	7,52E-01	14353,58	12537,36	6,38E-01
SPAC890.07c_g	11	1168,14	1016,93	1234,75	855,13	8,37E-01	18269,50	16676,46	17931,47	16421,05	8,12E-01	1038,29	942,27	3,72E-01	12387,96	12176,85	2,31E-02	1782,89	1209,34	3,07E-01	14353,58	13151,45	6,50E-02
SPAC890.08_a	5	1152,06	916,51	739,29	942,27	3,39E-01	52535,69	16676,46	50013,21	16421,05	9,60E-01	584,07	873,10	2,43E-01	47648,73	12176,85	8,70E-01	797,86	1499,22	7,87E-01	47594,25	13151,45	8,80E-01
SPAC8C9.03_a	10	661,68	820,30	588,13	526,39	1,63E-01	3481,22	47916,58	4189,17	46885,87	9,96E-01	714,11	584,07	4,65E-01	5049,54	44702,58	9,80E-01	600,49	814,63	8,25E-01	3883,41	46498,29	9,88E-01
SPAC8C9.05_a	4	317,37	544,96	242,19	401,71	5,14E-01	6740,49	3742,17	7529,42	4820,76	6,89E-01	190,02	621,67	9,27E-01	4863,88	5071,55	8,72E-01	205,07	584,07	8,84E-01	6669,18	3897,89	9,85E-01
SPAC8C9.05_c	4	1251,98	192,67	1200,98	302,33	9,70E-01	6740,49	6523,95	7529,42	6796,78	2,99E-01	955,43	230,72	8,59E-01	4863,88	5172,06	1,33E-02	1499,22	207,94	8,90E-01	6669,18	6172,04	5,17E-01
SPAC8C9.08_a	8	1663,49	1120,56	1192,69	1060,11	4,42E-01	48505,51	6523,95	48536,45	6796,78	9,96E-01	1136,20	867,07	3,27E-01	45641,21	5172,06	9,49E-01	1629,26	1458,23	6,47E-01	44134,63	6172,04	9,41E-01

SPAC8C9.11_b	4	455,09	1128,35	458,25	935,76	8,40E-01	10274,79	48859,61	10535,85	46887,14	9,77E-01	481,04	922,88	8,44E-01	9155,09	46823,76	9,59E-01	424,61	1606,83	7,73E-01	9864,59	43901,68	9,26E-01
SPAC8C9.14_b	29	7912,95	508,46	6517,03	448,82	8,93E-01	16888,82	11035,91	17936,58	8599,60	9,11E-01	5220,60	584,07	7,93E-01	16688,40	9524,33	8,70E-01	5634,22	576,03	8,28E-01	15569,13	12132,22	9,77E-01
SPAC8C9.14_d	6	1144,10	5442,30	1530,73	4770,75	9,63E-01	16888,82	16279,41	17936,58	17825,28	5,26E-02	975,50	4240,45	8,23E-01	16688,40	17443,59	4,25E-01	1168,14	5077,84	9,59E-01	15569,13	16588,53	4,84E-01
SPAC8E11.02c_a	17	3956,48	867,07	5148,73	826,00	8,49E-01	43538,38	16279,41	42938,97	17825,28	9,82E-01	4672,57	982,29	8,79E-01	42347,82	17443,59	9,99E-01	5220,60	1009,90	8,13E-01	39786,74	16588,53	9,32E-01
SPAC8E11.04c_b	4	174,85	2628,46	216,77	4608,24	7,27E-01	3686,26	42938,97	4119,26	42347,82	9,98E-01	209,38	3821,70	8,05E-01	3976,62	43841,21	9,85E-01	195,36	4182,07	7,69E-01	4092,89	39511,91	9,60E-01
SPAC8E11.04c_d	4	163,14	232,32	172,45	242,19	8,63E-01	3686,26	3742,95	4119,26	3555,40	7,07E-01	154,34	181,02	5,03E-01	3976,62	3488,29	9,49E-01	141,04	188,71	5,16E-01	4092,89	4088,69	5,66E-03
SPAC8E11.07c_b	5	1243,34	112,21	1820,35	138,14	7,94E-01	9152,82	3742,95	8248,98	3555,40	8,93E-01	1758,34	137,19	8,10E-01	8192,00	3488,29	8,81E-01	1884,54	154,34	7,72E-01	6653,97	4088,69	7,54E-01
SPAC9.02c_a	4	3492,39	1067,48	3516,68	1458,23	9,08E-01	25673,67	9741,98	24828,98	8719,32	9,42E-01	2876,30	1978,24	9,20E-01	22489,38	9410,14	8,80E-01	4011,71	1584,71	7,91E-01	22112,21	8023,41	8,27E-01
SPAC926.02_a	7	268,73	2521,38	261,38	2876,30	9,29E-01	1733,84	24567,09	1527,12	24133,41	9,86E-01	247,28	2977,74	9,13E-01	1750,68	23710,01	9,81E-01	265,03	4299,64	7,38E-01	2045,76	23817,32	9,90E-01
SPAC959.02_c	4	276,28	276,28	347,29	257,78	6,17E-01	3640,70	1935,92	4576,41	1602,70	8,77E-01	288,01	232,32	6,21E-01	4904,87	1719,10	7,99E-01	498,00	229,13	5,83E-01	5184,54	1965,95	7,08E-01
SPAC959.04c_a	4	227,54	218,27	282,09	304,44	2,83E-02	6685,58	3169,41	7811,55	4240,45	7,04E-01	225,97	310,83	3,98E-01	5868,20	3956,48	9,95E-01	230,72	377,41	3,85E-01	5347,31	4299,64	9,60E-01
SPAC9E9.04_a	12	274,37	218,27	296,11	259,57	4,46E-01	20276,79	7211,78	19487,17	7004,29	9,61E-01	321,80	222,86	6,92E-01	22793,75	5935,38	9,59E-01	337,79	278,20	2,71E-01	20755,89	6735,81	1,00E+00
SPAC9E9.13_a	8	831,75	225,97	3396,89	254,23	5,03E-01	24491,61	18815,91	30573,63	20325,43	5,83E-01	2019,80	242,19	5,87E-01	31216,04	24638,58	2,86E-01	604,67	302,33	8,45E-01	18561,17	22895,18	8,20E-01
SPAC9E9.17c_a	9	207,94	831,75	219,79	3236,01	5,15E-01	57,44	23170,48	58,20	30573,63	8,64E-01	206,50	2179,83	5,82E-01	87,58	34397,12	8,11E-01	222,86	699,41	8,95E-01	69,43	17805,06	8,71E-01
SPAC9G1.05_d	4	250,73	227,54	259,57	224,41	9,05E-01	9651,67	96,45	10094,56	62,33	9,79E-01	306,55	233,94	5,00E-01	7513,65	59,80	8,74E-01	282,09	242,19	4,24E-01	8183,81	95,35	9,17E-01
SPACUNK4.10_b	11	1200,98	306,55	1448,15	272,48	8,99E-01	32093,64	8866,41	31216,04	8988,12	9,83E-01	2256,70	257,78	6,91E-01	31651,80	6964,34	9,51E-01	2916,45	252,48	6,14E-01	31216,04	7661,68	9,56E-01
SPAP14E8.02_a	4	968,76	1448,15	1120,56	2033,85	5,49E-01	10028,67	31871,96	10478,56	31216,04	9,95E-01	861,08	2665,15	6,13E-01	8701,75	31433,17	9,60E-01	1379,57	3125,78	3,68E-01	10674,05	30573,63	9,84E-01
SPAP27G11.12_b	6	903,89	955,43	760,08	1024,00	8,06E-01	13485,48	11108,43	12951,99	10589,70	7,83E-01	831,75	935,76	5,12E-01	12010,64	8746,92	4,42E-01	792,35	1924,14	5,28E-01	12778,45	10730,53	7,62E-01
SPAP32A8.03c_a	6	670,92	617,37	955,43	634,73	4,51E-01	15825,90	13259,66	15500,21	13147,75	9,11E-01	699,41	814,63	2,18E-01	14972,21	13205,69	7,98E-01	843,36	680,29	3,04E-01	13493,72	13611,10	5,21E-01
SPAP7G5.02c_a	7	548,75	464,65	689,78	765,36	5,97E-02	16514,24	13682,08	16454,82	14066,74	9,38E-01	666,29	639,15	8,06E-02	12716,73	13873,08	3,60E-01	922,88	639,15	2,05E-01	15205,15	11425,74	5,29E-01
SPAP8A3.07c_a	10	522,76	471,14	448,82	515,56	7,60E-01	32318,16	15217,71	32029,55	15099,36	9,88E-01	393,44	588,13	9,57E-01	23766,14	11733,00	6,23E-01	530,06	709,18	3,19E-01	30777,54	14008,41	9,19E-01
SPAPB17E12.06_a	4	600,49	433,53	477,71	380,04	4,58E-01	2276,63	31437,04	1743,58	32051,12	9,99E-01	424,61	369,65	3,06E-01	1809,19	23719,88	8,43E-01	501,46	552,56	9,19E-01	2265,45	29409,65	9,64E-01
SPAPB17E12.10c_b	7	250,73	604,67	206,50	418,77	6,33E-01	915,98	2108,32	765,86	1739,20	7,68E-01	235,57	448,82	7,19E-01	787,44	1700,56	7,55E-01	319,57	494,56	9,26E-01	1051,34	2285,77	8,72E-01
SPAPB18E9.01_c	4	634,73	194,01	666,29	179,77	9,81E-01	1886,64	835,95	2058,28	646,10	9,93E-01	704,28	173,65	9,50E-01	1780,94	556,85	8,34E-01	776,05	280,14	7,64E-01	2458,05	943,75	7,48E-01
SPAPB18E9.02c_a	17	1698,45	484,38	1978,24	621,67	8,40E-01	5167,66	1537,28	3758,18	1598,70	7,80E-01	1807,78	445,72	9,73E-01	3849,22	1495,82	7,83E-01	2401,97	719,08	6,95E-01	4604,56	2172,88	9,88E-01
SPAPB1A10.10c_b	16	471,14	1992,00	560,28	2320,15	8,74E-01	7197,77	5527,23	7155,06	4152,23	7,20E-01	621,67	1663,49	9,32E-01	7502,91	4069,33	7,91E-01	781,44	2418,67	7,73E-01	7311,82	4968,63	8,91E-01
SPAPB1A10.12c_a	6	2076,59	474,41	1595,73	519,15	8,42E-01	23632,17	7158,69	23747,01	7053,74	1,00E+00	1710,26	474,41	8,73E-01	20759,10	7366,12	9,12E-01	2916,45	666,29	7,45E-01	19332,56	7308,74	8,58E-01
SPAPB1A10.15_a	13	560,28	2033,85	388,02	1652,00	8,02E-01	2386,80	21980,35	2535,42	21766,11	9,98E-01	380,04	1734,13	8,33E-01	2129,69	20507,27	9,55E-01	481,04	3281,18	7,47E-01	2866,76	20683,02	9,78E-01
SPAPB1A11.02_a	4	163,14	515,56	142,02	401,71	7,87E-01	78,69	1983,08	67,49	2224,28	9,44E-01	163,14	367,09	7,50E-01	106,91	1626,95	9,05E-01	174,85	404,50	8,35E-01	339,32	2207,52	8,72E-01
SPAPB2B4.02_a	14	396,18	166,57	410,15	224,41	8,31E-01	2494,82	91,95	2884,61	91,47	9,25E-01	445,72	186,11	8,60E-01	2524,55	95,12	9,93E-01	491,14	181,02	8,03E-01	2849,66	330,00	8,80E-01

SPAPB2B4.05_a	4	240,52	433,53	225,97	344,89	6,94E-01	5385,70	2727,19	5928,58	2298,50	9,82E-01	245,57	430,54	9,95E-01	5341,61	1951,18	8,67E-01	259,57	675,59	6,27E-01	4656,60	3295,84	9,62E-01
SPAPB2B4.05_d	4	604,67	219,79	739,29	210,84	8,65E-01	5385,70	5584,81	5928,58	5684,82	1,78E-01	541,19	266,87	9,75E-01	5341,61	5114,25	2,31E-01	621,67	265,03	9,16E-01	4656,60	5458,54	4,09E-01
SPAPB8E5.05_a	4	216,77	504,95	261,38	512,00	9,05E-01	401,71	5584,81	393,44	5684,82	9,91E-01	221,32	564,18	9,00E-01	257,78	5114,25	9,39E-01	219,79	572,05	8,92E-01	313,00	5458,54	9,79E-01
SPAPJ691.03_a	7	164,28	203,66	136,24	300,25	7,24E-01	15987,43	388,02	15667,01	298,17	9,87E-01	134,36	224,41	9,34E-01	15086,68	247,28	9,66E-01	160,90	216,77	9,00E-01	18389,65	296,11	9,32E-01
SPAPYUG7.04c_c	4	2272,40	154,34	2194,99	133,44	9,76E-01	26313,64	16292,95	27557,88	13977,23	9,55E-01	1509,65	154,34	7,90E-01	26662,72	14670,79	9,42E-01	1807,78	173,65	8,83E-01	26722,94	16553,22	9,67E-01
SPBC106.12c_a	6	458,25	1770,57	418,77	1418,35	8,34E-01	8974,57	27567,90	7210,28	27621,23	9,56E-01	442,64	1160,07	7,16E-01	5635,21	25154,30	8,51E-01	544,96	1871,53	9,29E-01	11427,79	27846,81	9,22E-01
SPBC106.12c_b	4	240,52	337,79	213,78	418,77	8,33E-01	8974,57	7937,05	7210,28	6207,66	1,36E-01	233,94	407,31	7,82E-01	5635,21	4144,74	5,91E-02	266,87	461,44	5,62E-01	11427,79	8473,14	4,41E-01
SPBC106.18_a	10	1910,85	200,85	1782,89	243,88	9,74E-01	13179,28	7937,05	13164,74	6207,66	8,60E-01	1698,45	202,25	9,34E-01	10422,42	4144,74	5,07E-01	2856,44	237,21	7,83E-01	11536,75	8473,14	8,72E-01
SPBC1105.08_a	5	820,30	1782,89	968,76	1209,34	7,10E-01	18082,53	12424,43	15895,48	14057,87	9,34E-01	820,30	1448,15	7,98E-01	14401,98	11596,03	5,49E-01	935,76	3281,18	5,90E-01	15028,87	11797,59	6,29E-01
SPBC1105.10_a	4	541,19	903,89	685,02	942,27	7,22E-01	2556,25	18215,23	2364,06	15260,76	8,91E-01	929,30	897,64	4,04E-01	2904,28	14498,09	8,79E-01	1251,98	1176,27	1,18E-01	2646,81	16333,87	9,39E-01
SPBC119.02_b	16	873,10	714,11	1082,39	781,44	5,02E-01	12379,19	2638,90	13060,78	2548,05	9,71E-01	1128,35	1287,18	6,64E-02	15303,10	3176,60	8,45E-01	903,89	1251,98	2,76E-01	14399,80	2873,83	8,95E-01
SPBC119.08_a	4	580,04	929,30	855,13	955,43	4,94E-01	19755,96	11825,65	19483,97	11858,77	9,85E-01	837,53	1031,12	4,63E-01	19755,96	14857,83	7,76E-01	982,29	760,08	6,30E-01	17682,08	13147,69	9,42E-01
SPBC119.08_b	11	754,83	369,65	600,49	685,02	7,23E-01	19728,87	17805,06	19510,86	17198,56	8,10E-01	670,92	588,13	7,65E-01	19788,80	18690,27	7,11E-01	630,35	709,18	6,39E-01	17736,98	17198,56	3,23E-01
SPBC119.14_b	6	179,77	484,38	128,00	427,57	8,23E-01	327,10	17840,73	346,02	17139,90	9,80E-01	186,11	494,56	9,73E-01	549,06	18668,25	9,71E-01	176,07	508,46	9,68E-01	753,55	17153,80	9,92E-01
SPBC1198.08_a	4	237,21	164,28	421,68	150,12	6,06E-01	13034,07	442,56	13307,94	439,36	9,89E-01	280,14	177,29	7,01E-01	12503,12	498,72	9,81E-01	451,94	183,55	4,89E-01	11746,96	662,71	9,55E-01
SPBC1198.13c_a	4	206,50	319,57	227,54	382,68	7,04E-01	9998,55	11910,94	11458,64	12245,81	4,77E-01	176,07	328,56	9,20E-01	12409,72	10369,08	7,85E-01	210,84	617,37	5,48E-01	11708,70	10441,20	9,26E-01
SPBC11B10.01_c	6	249,00	207,94	290,02	240,52	3,71E-01	2404,79	9901,13	2113,05	11271,19	9,36E-01	284,05	245,57	3,26E-01	1883,28	12674,51	8,80E-01	218,27	233,94	9,24E-01	2256,36	11812,04	8,98E-01
SPBC11B10.02c_b	7	3468,27	286,03	4576,41	357,05	8,44E-01	23444,29	2179,50	22637,83	1931,07	9,75E-01	4451,27	302,33	8,66E-01	18513,97	1596,78	8,58E-01	8192,00	290,02	6,35E-01	17770,83	1997,05	8,45E-01
SPBC11B10.07c_a	10	508,46	2876,30	685,02	3169,41	9,04E-01	15181,22	21722,19	15608,02	20809,01	9,59E-01	680,29	4039,61	7,76E-01	15716,58	19455,45	8,40E-01	625,99	7696,57	5,76E-01	13034,07	19697,95	6,99E-01
SPBC11G11.01_a	4	284,05	474,41	257,78	564,18	8,76E-01	4738,79	14562,80	4562,59	14868,79	9,94E-01	280,14	588,13	7,90E-01	6125,16	16046,82	8,56E-01	294,07	689,78	6,59E-01	5149,35	13400,51	9,59E-01
SPBC11G11.02c_a	8	385,34	261,38	430,54	265,03	8,35E-01	10567,28	4910,11	11091,60	4516,60	9,89E-01	580,04	261,38	6,27E-01	9814,96	5468,44	9,81E-01	885,29	328,56	4,25E-01	8605,23	5265,32	8,30E-01
SPBC11G11.03_a	13	298,17	471,14	369,65	374,81	8,99E-01	6517,03	10074,77	8779,97	10711,56	5,48E-01	319,57	584,07	7,12E-01	6208,38	9719,44	9,06E-01	477,71	897,64	3,14E-01	7912,95	9558,75	8,43E-01
SPBC11G11.06c_a	8	867,07	261,38	803,41	321,80	9,97E-01	3753,78	5330,30	2596,21	7231,10	8,93E-01	770,69	337,79	9,81E-01	2412,43	5220,60	6,96E-01	955,43	377,41	8,30E-01	2688,02	6208,38	9,66E-01
SPBC11G11.06c_b	4	661,68	689,78	526,39	797,86	9,30E-01	3753,78	2333,64	2596,21	2159,24	4,65E-01	498,00	826,00	9,41E-01	2412,43	1702,24	3,40E-01	596,34	989,12	6,13E-01	2688,02	2295,88	5,32E-01
SPBC1215.02c_f	9	190,02	544,96	210,84	494,56	9,54E-01	7989,53	2333,64	7738,74	2159,24	9,62E-01	195,36	458,25	8,71E-01	5772,75	1702,24	7,22E-01	229,13	689,78	7,82E-01	6231,03	2295,88	8,19E-01
SPBC1271.04c_a	5	225,97	207,94	232,32	163,14	6,45E-01	8086,08	7771,69	8641,72	8036,96	3,52E-01	238,86	173,65	7,82E-01	7911,26	5916,67	4,21E-01	344,89	221,32	4,00E-01	7826,53	6053,50	3,87E-01
SPBC1271.12_b	6	1640,59	256,00	1978,24	209,38	9,09E-01	6338,83	7454,46	6984,79	8995,55	4,42E-01	2836,70	196,72	7,40E-01	5792,62	7441,70	8,05E-01	2628,46	324,03	7,32E-01	5832,91	6270,32	2,94E-01
SPBC1271.12_c	6	1269,46	1260,69	1595,73	1722,16	2,49E-02	6338,83	5442,30	6984,79	6427,31	2,62E-01	1541,37	2702,35	2,78E-01	5792,62	5293,48	5,68E-01	1698,45	3373,43	2,68E-01	5832,91	5148,73	5,52E-01
SPBC1271.12_d	6	1795,29	1097,50	1552,09	1458,23	8,83E-01	6350,71	5442,30	6966,39	6427,31	2,69E-01	1458,23	1136,20	7,35E-01	5774,49	5293,48	5,54E-01	1552,09	1698,45	6,66E-01	5836,28	5148,73	5,52E-01
SPBC1289.02c_a	4	149,09	1398,83	229,13	1024,00	8,61E-01	3104,19	5459,89	3821,70	6416,64	6,80E-01	221,32	1217,75	9,52E-01	4482,23	5292,43	6,75E-01	210,84	1323,37	9,94E-01	3444,31	5142,69	9,94E-01

SPBC1289.03c_a	5	749,61	179,77	786,88	225,97	9,26E-01	33762,45	2936,74	33088,53	4096,00	9,92E-01	556,41	216,77	8,36E-01	31403,52	4124,49	9,80E-01	530,06	232,32	8,19E-01	31519,22	3125,78	9,65E-01
SPBC1289.03c_b	8	675,59	533,74	1269,46	719,08	3,04E-01	33689,23	29629,43	32995,92	31290,24	8,46E-01	955,43	617,37	4,26E-01	31433,17	31021,87	8,52E-01	1269,46	580,04	4,59E-01	31433,17	29450,96	6,44E-01
SPBC1289.04c_a	8	1351,18	600,49	1278,29	1152,06	5,94E-01	16600,81	29532,18	15692,88	31216,04	9,73E-01	1341,84	1052,79	6,37E-01	14208,03	31000,42	9,69E-01	1807,78	1305,15	3,27E-01	13926,24	29532,18	9,07E-01
SPBC12C2.08_a	4	224,41	1082,39	240,52	1278,29	8,89E-01	7304,94	16591,89	7702,19	14944,72	9,25E-01	212,31	1296,13	8,97E-01	7704,27	14583,64	9,02E-01	200,85	1746,20	7,52E-01	6468,20	14568,30	8,38E-01
SPBC12C2.10c_a	4	232,32	198,09	249,00	190,02	9,11E-01	9239,35	7542,28	8886,27	7398,00	8,46E-01	164,28	294,07	8,54E-01	9379,09	7683,99	9,17E-01	274,37	210,84	5,27E-01	8435,92	6848,90	5,85E-01
SPBC12C2.11_a	7	797,86	202,25	630,35	182,28	8,25E-01	9124,46	9228,54	8999,94	8585,77	2,14E-01	613,11	219,79	8,37E-01	9674,59	9372,74	1,62E-01	797,86	268,73	9,41E-01	10237,08	8699,78	7,41E-01
SPBC12D12.03_c	9	237,21	694,58	243,88	724,08	9,61E-01	10432,55	8383,42	10776,73	9373,69	6,45E-01	265,03	572,05	8,79E-01	9316,91	10009,52	8,35E-01	321,80	820,30	7,85E-01	9417,14	9359,13	9,86E-01
SPBC12D12.06_a	16	1184,45	245,57	1168,14	250,73	9,94E-01	4056,71	9411,63	3864,15	10249,70	9,45E-01	1332,57	278,20	9,10E-01	3662,22	8385,69	8,61E-01	1871,53	330,84	7,10E-01	4561,08	8929,19	9,98E-01
SPBC1347.07_c	5	1243,34	1112,82	1176,27	837,53	4,45E-01	2748,21	3984,15	2595,07	3500,42	7,18E-01	1060,11	1176,27	5,64E-01	2410,50	3480,88	6,58E-01	1884,54	1807,78	1,26E-02	2754,20	4173,30	9,27E-01
SPBC1348.12_b	5	337,79	1082,39	222,86	968,76	8,48E-01	205,74	2537,91	169,60	2537,08	9,92E-01	306,55	1052,79	9,59E-01	239,19	2072,53	8,98E-01	274,37	1360,57	8,85E-01	231,89	2483,14	9,94E-01
SPBC1348.12_d	4	455,09	187,40	330,84	184,82	7,18E-01	205,74	174,93	169,60	162,94	2,66E-01	388,02	272,48	9,56E-01	239,19	208,75	2,61E-01	364,56	242,19	9,14E-01	231,89	170,83	7,78E-01
SPBC13A2.01c_b	4	487,75	321,80	744,43	337,79	5,98E-01	10660,59	174,93	11505,21	162,94	9,62E-01	548,75	292,04	9,28E-01	9089,59	208,75	9,21E-01	522,76	430,54	5,28E-01	10015,87	170,83	9,68E-01
SPBC13A2.03_a	6	2062,24	359,54	1858,60	564,18	1,00E+00	17899,20	9026,81	17642,46	8841,04	9,75E-01	1795,29	445,72	9,41E-01	15105,77	6793,79	7,19E-01	2628,46	474,41	8,27E-01	14935,25	9541,50	8,35E-01
SPBC13E7.08c_a	7	630,35	1795,29	820,30	1520,15	9,56E-01	2778,33	15456,43	2778,33	15168,80	9,89E-01	776,05	1758,34	9,50E-01	2646,74	13129,71	8,95E-01	675,59	2759,13	7,14E-01	2469,49	14085,36	9,31E-01
SPBC13E7.10c_a	7	630,35	522,76	634,73	634,73	3,93E-01	1221,11	2936,74	1061,50	3040,30	9,85E-01	719,08	1016,93	2,07E-01	1179,72	2665,15	9,03E-01	760,08	1097,50	1,85E-01	1669,84	2628,46	9,50E-01
SPBC13G1.02_b	4	709,18	544,96	786,88	600,49	6,45E-01	17567,96	1178,96	17402,54	1017,78	9,90E-01	849,22	526,39	7,69E-01	16890,01	921,47	9,71E-01	1287,18	675,59	3,79E-01	14609,55	1415,30	9,09E-01
SPBC13G1.12_c	8	508,46	776,05	467,88	765,36	9,10E-01	3252,13	17025,14	3516,14	17255,46	9,82E-01	354,59	1260,69	7,60E-01	3619,32	17517,00	9,69E-01	584,07	1698,45	4,76E-01	3792,67	13473,77	8,75E-01
SPBC14C8.04_b	20	855,13	442,64	935,76	364,56	9,97E-01	19106,21	2758,52	20922,57	3104,83	9,37E-01	867,07	302,33	8,71E-01	17545,76	3062,81	9,59E-01	1112,82	380,04	8,38E-01	18068,66	3648,73	9,95E-01
SPBC14F5.06_a	17	1251,98	765,36	1332,57	855,13	8,26E-01	12174,61	17992,90	13534,17	20072,87	7,32E-01	1269,46	760,08	9,88E-01	13160,59	15090,96	7,84E-01	2452,44	1052,79	4,21E-01	14269,08	15997,16	9,88E-01
SPBC14F5.11c_a	5	448,82	975,50	481,04	1045,52	9,07E-01	3884,83	11990,46	3656,02	13810,54	9,14E-01	319,57	916,51	8,35E-01	4164,87	12923,18	9,28E-01	362,04	1951,00	6,49E-01	3822,67	14646,18	8,66E-01
SPBC14F5.13c_a	5	1217,75	347,29	1629,26	439,59	7,65E-01	8539,88	4141,50	9089,59	3771,12	9,82E-01	1675,06	317,37	8,16E-01	8135,41	4145,98	9,52E-01	1782,89	458,25	7,11E-01	7231,10	3694,56	7,85E-01
SPBC1539.04_a	8	310,83	873,10	362,04	1595,73	6,26E-01	25355,30	7590,61	29328,18	8599,28	8,72E-01	250,73	1686,71	6,73E-01	31000,42	7967,99	8,55E-01	242,19	1428,22	7,46E-01	25006,23	7231,10	9,80E-01
SPBC1539.07c_a	4	222,86	245,57	170,07	344,89	8,17E-01	3402,19	21769,19	3616,13	26987,43	8,72E-01	177,29	232,32	4,27E-01	3991,74	31651,80	7,82E-01	202,25	232,32	4,64E-01	4322,03	22381,20	9,58E-01
SPBC1539.07c_b	6	128,00	210,84	121,10	263,20	8,08E-01	3402,19	3173,70	3616,13	3638,21	9,80E-02	134,36	259,57	7,49E-01	3991,74	3323,34	4,05E-01	130,69	224,41	9,08E-01	4322,03	3579,64	2,30E-01
SPBC1539.08_b	8	1618,00	130,69	2019,80	130,69	8,83E-01	17438,64	3173,70	18561,17	3638,21	9,46E-01	2062,24	140,07	8,69E-01	18179,19	3323,34	9,69E-01	3125,78	133,44	6,96E-01	17559,94	3579,64	9,81E-01
SPBC1539.10_a	5	177,29	1562,89	194,01	1992,00	8,62E-01	1753,14	17559,94	2459,96	19349,38	9,24E-01	182,28	1710,26	9,48E-01	1822,76	19083,00	9,52E-01	225,97	2401,97	7,64E-01	1955,05	17559,94	9,94E-01
SPBC15C4.02_a	4	216,77	184,82	219,79	199,47	6,87E-01	2305,35	1671,45	2319,88	1800,16	8,77E-01	216,77	191,34	8,88E-01	2671,97	1604,54	8,32E-01	221,32	199,47	6,69E-01	2734,42	2503,76	2,02E-01
SPBC15D4.01c_a	5	729,11	272,48	739,29	202,25	9,40E-01	5698,73	1815,38	5476,85	2067,19	9,96E-01	831,75	194,01	9,78E-01	6792,17	2383,50	8,04E-01	734,19	254,23	9,86E-01	5062,04	2159,83	9,57E-01
SPBC15D4.11c_b	6	259,57	613,11	265,03	689,78	8,95E-01	3085,86	5472,72	2891,08	5312,60	9,26E-01	250,73	704,28	8,99E-01	3074,99	6568,28	8,22E-01	270,60	568,10	9,48E-01	2915,94	4931,37	8,41E-01
SPBC1604.21c_b	4	675,59	209,38	809,00	265,03	8,17E-01	20171,07	3184,69	20031,74	2859,37	9,86E-01	826,00	219,79	8,53E-01	19083,00	3118,55	9,65E-01	1360,57	242,19	6,14E-01	17318,18	3387,89	9,15E-01

SPBC1685.03_a	7	541,19	719,08	809,00	1074,91	1,91E-01	5113,16	20452,65	4608,24	19483,97	9,51E-01	1009,90	613,11	4,92E-01	4011,71	19755,96	9,42E-01	820,30	1287,18	2,32E-01	4420,52	18951,18	9,27E-01
SPBC1685.08_a	6	202,25	568,10	148,06	797,86	8,36E-01	679,94	4513,40	679,08	4153,18	9,51E-01	165,42	1176,27	6,48E-01	703,30	3396,89	8,37E-01	183,55	855,13	7,59E-01	840,76	3565,78	8,83E-01
SPBC1685.10_b	9	792,35	179,77	699,41	142,02	8,89E-01	15363,99	730,03	17622,40	829,15	9,25E-01	630,35	129,79	8,14E-01	16894,61	815,38	9,48E-01	652,58	183,55	8,76E-01	18158,05	725,97	9,14E-01
SPBC16A3.01_b	9	333,14	604,67	367,09	448,82	7,09E-01	21027,65	12126,61	20311,37	14409,87	8,97E-01	357,05	519,15	8,64E-01	18951,18	12288,27	8,79E-01	458,25	576,03	7,75E-01	18690,27	14050,23	9,71E-01
SPBC16A3.05c_b	19	388,02	300,25	337,79	410,15	6,52E-01	10568,95	22536,88	10624,54	20738,16	9,22E-01	337,79	369,65	8,56E-01	9224,80	18820,27	7,73E-01	372,22	519,15	3,57E-01	11272,87	21173,91	9,70E-01
SPBC16A3.08c_a	11	2936,74	354,59	1858,60	290,02	7,42E-01	31343,11	8822,30	30639,75	9462,22	9,99E-01	1468,37	300,25	6,45E-01	24077,37	8341,51	8,04E-01	2368,90	377,41	8,83E-01	21802,10	10071,30	7,75E-01
SPBC16A3.09c_b	7	333,14	2435,50	302,33	1269,46	6,57E-01	5139,85	29585,68	6152,13	30075,68	9,69E-01	272,48	1458,23	7,09E-01	5955,01	25007,14	9,14E-01	340,14	2304,12	9,69E-01	4878,54	21855,99	8,13E-01
SPBC16A3.12c_b	7	786,88	302,33	1136,20	317,37	7,39E-01	4269,94	4688,13	4513,40	5429,09	4,31E-01	809,00	286,03	9,94E-01	4153,18	4742,15	9,39E-01	982,29	306,55	8,33E-01	3875,05	4438,03	4,55E-01
SPBC16C6.07c_a	8	288,01	765,36	274,37	897,64	8,94E-01	5817,50	3304,00	6259,28	3902,01	7,91E-01	374,81	770,69	8,96E-01	7357,83	3304,00	7,77E-01	364,56	873,10	8,16E-01	6312,10	3848,29	7,96E-01
SPBC16C6.08c_c	4	413,00	304,44	471,14	319,57	7,32E-01	7522,79	6468,91	7841,98	6635,09	7,91E-01	415,87	319,57	9,13E-01	7649,89	7725,95	3,20E-01	530,06	354,59	5,03E-01	8696,38	6417,07	6,99E-01
SPBC16D10.07c_c	4	171,25	471,14	164,28	433,53	9,22E-01	663,33	7840,82	753,86	6992,88	9,44E-01	159,79	498,00	9,76E-01	531,07	7487,41	9,66E-01	202,25	504,95	8,93E-01	888,67	8227,07	9,58E-01
SPBC16D10.07c_e	6	213,78	188,71	215,27	177,29	8,48E-01	663,33	621,48	753,86	744,73	3,79E-02	242,19	188,71	6,78E-01	531,07	478,16	5,50E-02	315,17	195,36	4,71E-01	888,67	688,36	2,89E-01
SPBC16D10.11c_a	7	245,57	215,27	315,17	156,50	9,53E-01	15344,07	621,48	15811,45	744,73	9,80E-01	310,83	233,94	4,17E-01	13260,72	478,16	9,20E-01	302,33	296,11	4,70E-02	11669,59	688,36	8,62E-01
SPBC16E9.02c_a	9	1734,13	235,57	2702,35	263,20	7,61E-01	7434,40	13855,05	7434,40	15948,96	8,62E-01	2149,82	335,46	8,47E-01	6653,97	13165,84	8,87E-01	2610,30	317,37	7,60E-01	6746,86	9795,09	5,73E-01
SPBC16E9.11c_a	13	584,07	1992,00	621,67	2977,74	7,45E-01	11685,71	8079,22	11884,01	8079,22	9,73E-01	625,99	2241,11	9,04E-01	11590,59	6841,04	8,44E-01	689,78	2856,44	7,43E-01	11874,95	7281,40	9,27E-01
SPBC16E9.19_a	9	333,14	451,94	284,05	617,37	7,74E-01	2352,37	12869,98	2131,15	12457,55	9,70E-01	354,59	537,45	6,72E-01	2747,34	12524,02	9,98E-01	367,09	541,19	6,18E-01	3186,46	12211,39	9,91E-01
SPBC16G5.05c_a	10	729,11	288,01	501,46	288,01	6,88E-01	30261,33	1927,26	26460,01	1942,99	9,29E-01	652,58	304,44	9,25E-01	24632,52	1895,31	8,90E-01	916,51	362,04	7,48E-01	23819,10	1920,59	8,74E-01
SPBC16G5.07c_b	4	146,02	504,95	128,00	548,75	9,67E-01	3161,15	28302,66	3361,04	26409,59	9,65E-01	195,36	592,22	8,22E-01	3768,86	26490,62	9,75E-01	132,51	929,30	6,85E-01	4004,77	23515,84	9,13E-01
SPBC16G5.10_a	4	584,07	141,04	709,18	128,00	8,92E-01	13034,07	3161,41	13873,08	3302,03	9,52E-01	519,15	162,02	9,45E-01	10586,95	3373,31	8,72E-01	1314,23	136,24	6,23E-01	12590,08	3664,34	9,97E-01
SPBC16G5.12c_a	4	335,46	556,41	296,11	814,63	7,35E-01	4673,29	13682,08	4604,42	12416,75	9,21E-01	427,57	831,75	5,09E-01	5558,24	10586,95	8,50E-01	385,34	1332,57	4,85E-01	5585,09	13969,57	9,31E-01
SPBC16G5.14c_a	11	1606,83	335,46	1209,34	374,81	8,36E-01	55527,36	5678,19	55473,71	5174,48	9,94E-01	1002,93	436,55	7,52E-01	51418,08	6265,55	9,63E-01	2256,70	369,65	7,92E-01	52666,80	6445,48	9,78E-01
SPBC16G5.15c_a	12	288,01	1351,18	324,03	942,27	7,90E-01	7402,19	55512,38	8647,77	55811,80	9,84E-01	335,46	935,76	7,92E-01	8556,41	53455,63	9,90E-01	362,04	2435,50	6,68E-01	6215,92	54645,21	9,79E-01
SPBC16G5.16_a	10	1192,69	280,14	1089,92	342,51	9,76E-01	10455,00	7796,19	9327,77	8491,73	8,91E-01	1499,22	342,51	8,26E-01	10274,28	9174,81	7,18E-01	1858,60	352,14	7,16E-01	9615,40	6500,58	6,54E-01
SPBC16G5.16_b	5	765,36	867,07	680,29	1045,52	8,28E-01	10455,00	11416,62	9327,77	10416,64	2,81E-01	797,86	996,00	5,44E-01	10274,28	11381,61	8,97E-01	982,29	1488,87	2,46E-01	9615,40	10907,62	4,91E-01
SPBC16H5.04_a	4	393,44	670,92	396,18	648,07	9,62E-01	16751,78	11416,62	16577,44	10416,64	8,99E-01	458,25	699,41	8,23E-01	13348,85	11381,61	6,07E-01	474,41	837,53	6,42E-01	15346,70	10907,62	8,09E-01
SPBC16H5.07c_a	13	288,01	369,65	448,82	445,72	1,01E-01	17079,76	16560,67	17198,56	16358,03	9,40E-01	342,51	349,71	7,14E-01	17928,91	13956,22	7,04E-01	335,46	487,75	4,39E-01	15393,14	17891,03	9,02E-01
SPBC16H5.08c_a	4	195,36	224,41	218,27	340,14	3,84E-01	25766,91	14868,79	26442,34	16270,83	9,02E-01	179,77	294,07	6,92E-01	23287,63	16384,00	9,47E-01	199,47	321,80	5,04E-01	24023,31	13777,25	8,67E-01
SPBC16H5.12c_b	12	1031,12	199,47	1251,98	221,32	8,71E-01	7059,10	24522,71	6473,62	25552,13	9,88E-01	1200,98	225,97	8,92E-01	7416,98	23758,10	9,88E-01	1438,15	219,79	7,99E-01	6149,41	24553,58	9,76E-01
SPBC1703.04_a	5	148,06	873,10	188,71	1031,12	8,75E-01	556,64	7034,40	508,73	6799,45	9,78E-01	219,79	942,27	9,03E-01	870,59	7171,98	9,65E-01	154,34	1323,37	7,72E-01	900,23	5854,88	9,28E-01
SPBC1703.05_a	6	163,14	173,65	209,38	154,34	6,78E-01	957,70	563,27	938,21	514,65	9,17E-01	218,27	178,53	2,82E-01	910,31	796,99	6,94E-01	209,38	164,28	5,09E-01	1130,47	723,72	6,16E-01

SPBC1703.07_b	11	292,04	173,65	254,23	154,34	7,48E-01	15023,13	834,21	14770,00	911,23	9,94E-01	321,80	187,40	8,31E-01	13267,53	755,90	9,32E-01	380,04	190,02	6,87E-01	13117,23	890,08	9,30E-01
SPBC1703.10_a	7	445,72	219,79	464,65	230,72	9,35E-01	12239,94	13687,33	11772,99	15001,05	8,33E-01	337,79	268,73	8,26E-01	10755,21	12287,45	3,05E-01	415,87	315,17	8,16E-01	9812,76	11224,16	1,37E-01
SPBC1703.10_d	7	2210,26	324,03	3902,01	398,93	7,00E-01	12245,81	11057,70	11746,96	11630,65	9,56E-01	3769,09	306,55	7,34E-01	10734,74	10702,35	2,57E-01	6208,38	481,04	5,62E-01	9809,75	11103,98	3,07E-01
SPBC1703.11_b	5	464,65	1858,60	734,19	3444,31	6,05E-01	3281,18	11036,54	4124,49	11665,82	9,04E-01	734,19	3169,41	6,30E-01	3902,01	10734,74	9,78E-01	1618,00	6427,31	3,71E-01	3875,05	11113,30	9,55E-01
SPBC1709.02c_a	5	163,14	433,53	174,85	670,92	7,02E-01	18235,69	2610,30	17951,86	3541,14	9,78E-01	177,29	494,56	8,74E-01	13754,63	3492,39	8,65E-01	219,79	1234,75	5,00E-01	13667,71	3258,52	8,54E-01
SPBC1709.06_a	9	219,79	173,65	235,57	202,25	5,17E-01	556,88	17367,30	569,09	17230,00	9,96E-01	252,48	160,90	8,64E-01	464,71	13513,93	8,70E-01	227,54	242,19	2,56E-01	615,83	13573,32	8,77E-01
SPBC1709.10c_a	5	968,76	188,71	879,17	230,72	9,67E-01	28614,37	513,55	27788,46	546,12	9,86E-01	781,44	188,71	8,66E-01	25837,68	400,94	9,46E-01	608,87	256,00	7,65E-01	24278,72	477,52	9,16E-01
SPBC1709.12_a	6	481,04	724,08	596,34	916,51	5,24E-01	5480,15	29553,40	4576,41	27295,01	9,33E-01	552,56	657,11	9,88E-01	4672,57	26307,51	9,12E-01	630,35	879,17	4,74E-01	4870,99	27801,07	9,50E-01
SPBC1709.16c_c	7	415,87	448,82	369,65	600,49	6,95E-01	3077,51	5148,73	2922,06	4359,66	7,44E-01	427,57	548,75	4,68E-01	3654,03	4124,49	8,53E-01	436,55	797,86	4,15E-01	4181,25	4389,98	8,84E-01
SPBC1709.17_b	13	268,73	393,44	284,05	410,15	8,73E-01	1899,01	2890,65	2277,66	2898,85	7,72E-01	401,71	445,72	2,96E-01	1968,65	3220,37	8,26E-01	344,89	596,34	4,25E-01	2564,33	3810,54	4,24E-01
SPBC1711.03_c	8	436,55	235,57	580,04	224,41	7,77E-01	843,36	1840,75	1060,11	2100,39	7,72E-01	580,04	333,14	5,28E-01	935,76	1668,96	9,55E-01	666,29	333,14	4,89E-01	891,44	2349,63	7,82E-01
SPBC1711.08_a	17	512,00	369,65	2048,00	481,04	4,05E-01	11505,21	820,30	17805,06	897,64	7,80E-01	1314,23	544,96	3,38E-01	21920,61	831,75	7,02E-01	498,00	792,35	3,38E-01	7590,61	891,44	7,89E-01
SPBC1718.05_c	9	296,11	430,54	477,71	1468,37	3,47E-01	989,12	11190,60	1105,13	17198,56	7,78E-01	333,14	897,64	4,77E-01	1038,29	22073,07	6,86E-01	390,72	404,50	6,62E-01	1160,07	6793,79	7,52E-01
SPBC1734.05c_a	9	190,02	310,83	230,72	349,71	6,85E-01	1428,59	1082,39	1711,94	1200,98	5,82E-01	195,36	393,44	7,41E-01	1718,79	1192,69	5,90E-01	225,97	377,41	6,49E-01	1620,01	1120,56	7,42E-01
SPBC1734.08_b	8	282,09	205,07	333,14	227,54	6,30E-01	2229,79	1326,99	3877,02	1542,83	5,34E-01	274,37	206,50	9,57E-01	4143,09	1452,48	5,47E-01	288,01	238,86	7,06E-01	2870,80	1606,43	6,14E-01
SPBC1734.10c_a	18	922,88	256,00	1024,00	286,03	9,07E-01	12597,83	2655,48	13013,18	3504,59	9,35E-01	1152,06	296,11	8,27E-01	13237,09	3605,81	9,19E-01	1060,11	304,44	8,71E-01	12206,43	2568,42	9,76E-01
SPBC1734.14c_b	4	739,29	604,67	724,08	724,08	5,20E-01	6128,59	11191,11	6067,38	12163,85	9,19E-01	608,87	734,19	9,97E-01	5256,79	12539,09	9,62E-01	694,58	670,92	8,89E-01	5671,00	11266,27	9,64E-01
SPBC1773.07c_b	7	304,44	533,74	310,83	776,05	6,79E-01	6716,28	5714,08	7231,14	5966,99	6,81E-01	317,37	643,59	7,87E-01	8643,79	4969,26	7,86E-01	364,56	541,19	8,37E-01	7906,26	6143,39	5,08E-01
SPBC1773.09c_a	6	2452,44	286,03	1992,00	280,14	8,81E-01	2324,09	6553,70	2129,92	7491,71	9,23E-01	1746,20	317,37	8,19E-01	1933,91	8330,71	8,73E-01	1858,60	328,56	8,55E-01	2151,86	6811,62	9,90E-01
SPBC1778.03c_a	6	975,50	2418,67	1038,29	1746,20	7,41E-01	2202,42	1967,95	2175,23	2067,09	8,07E-01	1176,27	1698,45	7,67E-01	2616,41	1859,54	7,37E-01	1209,34	2336,28	9,42E-01	2563,76	2075,87	4,77E-01
SPBC1778.05c_a	5	487,75	797,86	533,74	1031,12	6,81E-01	2907,94	2072,98	3329,46	2040,06	8,24E-01	541,19	1160,07	6,09E-01	2872,62	2015,96	9,45E-01	680,29	1089,92	4,45E-01	4124,17	1917,23	6,97E-01
SPBC1778.07_a	4	242,19	541,19	240,52	515,56	9,53E-01	3710,99	1963,74	3688,99	2162,43	9,46E-01	257,78	474,41	9,02E-01	3108,68	2317,06	9,09E-01	349,71	580,04	7,36E-01	3562,12	3152,66	6,21E-01
SPBC17A3.02_d	9	1562,89	274,37	996,00	278,20	7,39E-01	4217,99	3067,85	3283,76	3335,97	6,21E-01	1370,04	229,13	9,03E-01	3850,18	2777,37	7,16E-01	1758,34	344,89	9,02E-01	3639,26	2931,82	6,49E-01
SPBC17A3.03c_c	4	268,73	1296,13	252,48	873,10	7,49E-01	5274,85	3616,44	4618,38	3200,59	6,72E-01	225,97	1278,29	9,71E-01	4366,85	3449,93	6,28E-01	254,23	2076,59	7,49E-01	5307,76	3653,82	9,79E-01
SPBC17A3.08_b	6	304,44	242,19	326,29	233,94	9,14E-01	3328,33	4786,95	3204,13	4262,90	7,54E-01	280,14	242,19	7,71E-01	2866,24	3759,78	4,76E-01	266,87	225,97	5,45E-01	3096,65	4599,87	8,60E-01
SPBC17D1.01_b	4	2998,45	242,19	2105,58	282,09	8,20E-01	32425,22	2794,32	29202,08	2826,09	9,43E-01	2272,40	272,48	8,57E-01	30184,50	2460,75	9,55E-01	2336,28	282,09	8,73E-01	31255,11	2505,29	9,75E-01
SPBC17D1.03c_a	4	179,77	2241,11	148,06	2179,83	9,77E-01	2405,09	31645,95	3278,28	27654,38	9,42E-01	146,02	2076,59	9,50E-01	3526,27	31500,10	9,83E-01	124,50	2435,50	9,68E-01	2770,03	31446,89	9,97E-01
SPBC17D11.06_b	8	526,39	152,22	404,50	129,79	7,85E-01	4532,31	1978,76	4111,62	2904,65	8,75E-01	436,55	137,19	8,47E-01	3999,53	2916,74	8,97E-01	657,11	146,02	8,62E-01	4523,41	2181,61	9,60E-01
SPBC17D11.08_a	4	843,36	477,71	1038,29	352,14	9,37E-01	7163,47	4309,68	6795,02	3655,64	8,32E-01	1002,93	512,00	7,81E-01	7385,63	3673,48	9,38E-01	922,88	580,04	7,52E-01	6773,58	4626,95	9,86E-01
SPBC17G9.08c_c	4	159,79	749,61	181,02	837,53	9,13E-01	4359,00	6795,61	4388,11	6459,90	9,32E-01	170,07	781,44	9,65E-01	3951,87	6912,97	9,47E-01	163,14	948,83	8,56E-01	4120,00	6512,92	8,93E-01

SPBC1861.04c_b	4	207,94	203,66	164,28	212,31	5,43E-01	2137,24	3977,60	2032,90	4358,50	9,34E-01	209,38	159,79	4,84E-01	1915,21	3572,73	8,24E-01	250,73	172,45	8,96E-01	2429,07	3996,16	9,10E-01
SPBC18H10.02_a	5	560,28	242,19	1112,82	194,01	6,56E-01	25709,25	2240,74	25709,25	1958,93	9,94E-01	515,56	237,21	9,17E-01	22381,20	1915,44	9,17E-01	491,14	212,31	8,37E-01	15181,22	2532,40	7,38E-01
SPBC18H10.04c_a	6	1820,35	352,14	2225,63	699,41	7,56E-01	26801,01	25709,25	29737,59	26249,46	4,42E-01	1992,00	544,96	8,76E-01	25355,30	24322,43	2,00E-01	2048,00	526,39	8,67E-01	26432,04	16158,44	4,38E-01
SPBC18H10.08c_a	8	975,50	1398,83	982,29	1734,13	7,30E-01	6956,85	27364,16	6378,60	29328,18	9,68E-01	1002,93	1260,69	8,44E-01	5707,76	23331,64	8,63E-01	1468,37	1782,89	2,38E-01	6889,13	24154,43	9,14E-01
SPBC18H10.12c_a	19	1924,14	803,41	1438,15	867,07	7,69E-01	44667,30	6833,36	43203,51	6210,87	9,72E-01	1341,84	1112,82	8,34E-01	38883,81	6137,93	9,09E-01	1698,45	2149,82	4,52E-01	40082,76	7118,21	9,40E-01
SPBC18H10.14_a	7	179,77	1478,58	237,21	1234,75	9,20E-01	38200,44	44444,58	38658,74	42262,48	8,33E-01	212,31	1468,37	9,91E-01	35187,79	41515,66	5,73E-01	227,54	1884,54	8,49E-01	35697,15	39377,46	4,06E-01
SPBC18H10.19_a	10	1351,18	238,86	1562,89	187,40	9,36E-01	21260,27	36075,70	19289,38	35808,85	9,29E-01	1120,56	179,77	8,61E-01	19673,84	32621,07	8,22E-01	1351,18	195,36	9,81E-01	20349,76	32016,61	8,17E-01
SPBC1921.01c_a	5	1360,57	1060,11	1332,57	955,43	8,09E-01	36669,58	19826,27	34557,17	19015,43	9,10E-01	1209,34	1060,11	6,96E-01	29379,85	20363,27	7,58E-01	1675,06	1176,27	5,37E-01	32415,38	22134,24	9,30E-01
SPBC19C2.08_a	8	219,79	1045,52	404,50	1192,69	7,99E-01	439,59	32740,50	694,58	32437,50	9,99E-01	191,34	1389,16	8,49E-01	617,37	30895,00	9,73E-01	192,67	1820,35	7,22E-01	617,37	30874,48	9,73E-01
SPBC19C2.10_a	4	680,29	212,31	689,78	328,56	8,51E-01	6165,30	458,25	6022,88	814,63	9,80E-01	666,29	194,01	9,66E-01	6228,60	526,39	9,88E-01	962,07	232,32	7,61E-01	5695,53	477,71	9,59E-01
SPBC19C2.12_b	4	256,00	699,41	276,28	455,09	6,85E-01	5336,32	5960,35	5700,28	5578,58	9,80E-01	215,27	754,83	9,85E-01	4875,76	5638,18	5,10E-01	290,02	910,17	7,79E-01	5000,92	5766,65	6,46E-01
SPBC19F5.04_a	16	1640,59	278,20	1370,04	250,73	8,81E-01	15660,40	4538,65	15055,36	5317,15	9,92E-01	2076,59	252,48	8,74E-01	12017,62	5017,74	8,32E-01	2702,35	315,17	7,28E-01	13057,12	4325,33	8,61E-01
SPBC19F5.05c_a	7	1060,11	1398,83	1360,57	1200,98	8,10E-01	6608,01	15740,48	7434,40	14714,76	9,88E-01	1184,45	1910,85	5,10E-01	6793,79	11798,56	7,53E-01	1152,06	2486,67	4,82E-01	7082,29	13077,96	8,60E-01
SPBC19F8.01c_c	5	337,79	948,83	377,41	1152,06	8,29E-01	1575,25	6562,36	1472,06	6562,36	9,90E-01	458,25	968,76	8,76E-01	2173,96	6080,61	9,87E-01	367,09	1640,59	6,60E-01	2022,58	6472,02	9,62E-01
SPBC19G7.03c_a	5	461,44	328,56	418,77	369,65	9,92E-01	19116,93	1644,65	18924,81	1539,70	9,91E-01	487,75	484,38	3,04E-01	15571,00	2270,52	9,06E-01	430,54	380,04	8,98E-01	18158,51	2173,21	9,87E-01
SPBC19G7.06_b	6	3590,58	430,54	2418,67	388,02	7,77E-01	10076,35	15120,40	7305,87	17871,65	9,99E-01	1992,00	396,18	6,90E-01	7586,00	13734,05	6,74E-01	2120,22	421,68	7,20E-01	8074,89	15349,95	8,60E-01
SPBC19G7.17_d	8	837,53	3304,00	891,44	2225,63	7,50E-01	20297,23	10460,62	20362,65	7473,35	8,74E-01	734,19	1795,29	6,09E-01	17840,58	7635,08	7,45E-01	885,29	2503,97	8,23E-01	17022,96	8225,77	7,17E-01
SPBC1D7.01_a	5	407,31	584,07	326,29	501,46	5,78E-01	5001,34	18842,36	4882,28	18901,14	9,98E-01	268,73	501,46	5,28E-01	4837,58	15537,70	8,61E-01	335,46	929,30	7,02E-01	5115,40	15628,90	8,75E-01
SPBC20F10.09_a	4	203,66	324,03	222,86	300,25	9,77E-01	22602,06	4986,72	24415,14	4709,24	9,59E-01	238,86	265,03	8,65E-01	19876,71	5067,83	9,19E-01	259,57	347,29	6,48E-01	20752,55	6268,41	9,82E-01
SPBC21.03c_b	6	170,07	190,02	172,45	221,32	5,89E-01	716,31	20371,87	807,92	19651,99	9,84E-01	177,29	232,32	4,87E-01	836,72	18854,59	9,63E-01	196,72	237,21	2,43E-01	834,38	20917,53	9,83E-01
SPBC21.04_a	5	296,11	154,34	300,25	182,28	8,78E-01	11357,50	722,26	10437,07	800,04	9,59E-01	190,02	153,28	5,40E-01	9745,80	762,02	9,20E-01	259,57	206,50	9,27E-01	9422,65	727,11	9,01E-01
SPBC21B10.08c_a	10	1038,29	294,07	1871,53	278,20	6,88E-01	28133,47	10791,48	29328,18	10415,28	9,77E-01	1418,35	310,83	7,94E-01	29944,43	10401,09	9,62E-01	1038,29	333,14	9,73E-01	23331,64	10796,91	8,43E-01
SPBC21B10.09_c	4	252,48	1105,13	252,48	1924,14	7,05E-01	8907,53	27939,14	7886,60	29532,18	9,86E-01	265,03	1686,71	7,54E-01	7038,67	32093,64	9,49E-01	347,29	1097,50	9,46E-01	7311,44	26249,46	9,14E-01
SPBC21C3.05_a	13	1897,65	212,31	1951,00	151,17	9,98E-01	12739,90	8055,77	12441,52	7730,27	9,34E-01	2538,92	216,77	8,43E-01	13279,43	6449,18	9,09E-01	2856,44	290,02	7,68E-01	12852,75	6732,84	8,90E-01
SPBC21C3.09c_a	6	3795,30	1332,57	3326,99	1458,23	9,22E-01	15806,40	11549,42	15729,27	10807,33	9,11E-01	3040,30	1910,85	9,54E-01	13338,86	11893,90	6,83E-01	4240,45	2721,15	5,91E-01	17025,53	12146,59	8,05E-01
SPBC21C3.13_a	5	1217,75	3565,78	704,28	3420,52	8,71E-01	22110,57	15501,33	22392,97	15156,90	9,96E-01	576,03	4576,41	9,44E-01	19460,07	13832,16	6,68E-01	826,00	5184,54	8,27E-01	20093,77	17306,74	9,79E-01
SPBC21C3.18_a	5	498,00	942,27	415,87	560,28	4,25E-01	161,59	19749,93	158,62	19830,87	9,98E-01	342,51	504,95	3,37E-01	215,85	18305,45	9,63E-01	304,44	897,64	7,78E-01	166,90	17053,63	9,27E-01
SPBC21C3.18_b	8	249,00	243,88	235,57	290,02	6,10E-01	161,59	204,34	158,62	181,71	6,51E-01	235,57	284,05	6,38E-01	215,85	192,12	4,80E-01	192,67	268,73	7,20E-01	166,90	170,39	5,73E-01
SPBC21D10.09c_a	6	826,00	247,28	1341,84	202,25	7,48E-01	5330,30	204,34	5712,87	181,71	9,66E-01	1074,91	200,85	8,65E-01	5442,30	192,12	9,90E-01	1168,14	209,38	8,11E-01	4576,41	170,39	9,18E-01
SPBC21H7.06c_a	4	1722,16	781,44	1782,89	897,64	9,04E-01	18132,52	4973,34	19243,39	5556,65	9,37E-01	1341,84	724,08	7,35E-01	22039,58	5367,37	8,58E-01	1530,73	942,27	9,81E-01	18160,40	4737,79	9,92E-01

SPBC23E6.05_a	4	639,15	1341,84	689,78	1509,65	8,58E-01	23815,37	17615,12	25308,39	18588,65	8,13E-01	484,38	1686,71	9,04E-01	21216,60	23125,79	6,97E-01	739,29	2149,82	6,23E-01	23935,21	19308,19	8,37E-01
SPBC23E6.08_a	11	849,22	744,43	760,08	639,15	3,48E-01	2398,98	23167,96	2376,78	23864,42	9,84E-01	982,29	568,10	9,29E-01	2363,21	21309,78	9,52E-01	1192,69	843,36	3,49E-01	2992,28	24044,78	9,65E-01
SPBC23G7.05_a	9	163,14	803,41	183,55	625,99	8,59E-01	1762,41	2677,23	2115,76	2598,76	8,15E-01	188,71	867,07	9,33E-01	2041,72	2213,32	8,61E-01	177,29	1009,90	8,53E-01	1867,89	2670,50	9,43E-01
SPBC244.01c_a	10	694,58	172,45	1112,82	173,65	7,34E-01	3640,70	1711,27	3717,20	2266,40	8,18E-01	1499,22	182,28	6,23E-01	4737,79	1718,66	7,87E-01	2352,53	192,67	5,29E-01	4705,07	1750,75	7,84E-01
SPBC24C6.02_a	5	427,57	670,92	461,44	1024,00	5,92E-01	5408,64	3983,99	5776,43	3717,20	9,71E-01	407,31	1217,75	5,97E-01	5043,34	5042,77	6,75E-01	501,46	2336,28	4,47E-01	6294,35	5148,73	3,79E-01
SPBC24C6.10c_a	4	541,19	306,55	600,49	278,20	9,45E-01	11848,42	4872,79	10232,01	5331,38	9,04E-01	935,76	357,05	5,50E-01	10379,43	4546,60	8,62E-01	1209,34	552,56	3,20E-01	11132,72	5897,34	9,75E-01
SPBC24C6.13_a	6	252,48	354,59	230,72	461,44	7,68E-01	4795,13	10990,77	3547,78	8396,48	6,74E-01	232,32	568,10	6,37E-01	5529,10	9076,43	8,84E-01	280,14	739,29	4,73E-01	6336,03	10911,89	8,67E-01
SPBC25B2.03_a	10	948,83	227,54	873,10	221,32	9,40E-01	16773,08	3634,43	15832,11	4512,10	9,97E-01	719,08	215,27	8,09E-01	16157,68	4349,61	9,96E-01	685,02	233,94	7,91E-01	14914,14	5061,07	9,81E-01
SPBC25B2.06c_a	8	337,79	530,06	388,02	544,96	8,18E-01	1881,10	16861,20	3241,80	15476,94	9,99E-01	302,33	436,55	6,38E-01	2730,57	16726,30	9,75E-01	377,41	588,13	7,65E-01	2231,79	15664,23	9,70E-01
SPBC25H2.10c_a	5	388,02	272,48	439,59	282,09	7,84E-01	3147,52	1714,25	3191,46	3349,52	3,64E-01	584,07	313,00	5,06E-01	2592,27	2385,00	9,44E-01	477,71	302,33	6,27E-01	2817,11	2076,26	9,86E-01
SPBC25H2.12c_b	4	219,79	382,68	330,84	541,19	4,18E-01	15286,81	2740,08	14766,09	3040,30	9,91E-01	304,44	630,35	4,58E-01	13587,57	2288,20	9,10E-01	328,56	388,02	5,78E-01	15286,81	2556,58	9,93E-01
SPBC25H2.14_a	4	352,14	235,57	385,34	278,20	6,79E-01	13174,69	13307,94	13821,49	14972,21	1,84E-01	362,04	276,28	7,60E-01	12493,29	12503,12	7,99E-03	617,37	330,84	3,64E-01	12097,10	13969,57	8,45E-01
SPBC25H2.16c_c	10	719,08	410,15	436,55	382,68	4,27E-01	16946,37	13507,34	16742,80	13057,22	9,09E-01	445,72	362,04	4,21E-01	13543,29	12574,30	3,49E-01	648,07	564,18	8,20E-01	14104,65	13515,38	5,02E-01
SPBC26H8.05c_b	4	304,44	481,04	265,03	374,81	5,56E-01	10877,55	14874,15	10202,98	14918,14	9,28E-01	238,86	385,34	5,55E-01	11280,38	13732,05	8,89E-01	321,80	600,49	7,19E-01	10065,64	13334,45	6,93E-01
SPBC26H8.10_a	6	259,57	213,78	326,29	257,78	3,11E-01	3590,58	9230,24	3373,43	9603,91	9,87E-01	367,09	296,11	1,54E-01	3516,68	9696,22	9,67E-01	324,03	294,07	1,18E-01	3691,52	8960,68	9,85E-01
SPBC27B12.01c_b	6	250,73	209,38	272,48	274,37	1,71E-01	7906,55	4269,94	7367,25	4299,64	9,24E-01	252,48	256,00	3,64E-01	9224,01	4240,45	8,54E-01	369,65	268,73	2,44E-01	7868,20	3821,70	9,37E-01
SPBC27B12.07_a	4	286,03	290,02	298,17	270,60	8,18E-01	9853,56	7852,04	9011,45	7582,49	6,96E-01	418,77	257,78	5,96E-01	9078,29	8444,52	9,39E-01	352,14	302,33	2,57E-01	9124,12	7735,49	7,62E-01
SPBC27B12.14_b	6	837,53	222,86	1428,22	235,57	6,97E-01	11036,54	10350,69	14766,09	8042,11	8,53E-01	1478,58	337,79	6,19E-01	20882,40	7461,38	6,56E-01	1408,55	344,89	6,29E-01	11505,21	9714,86	9,38E-01
SPBC28E12.03_a	5	1332,57	580,04	1992,00	1038,29	4,55E-01	7912,95	7486,11	7643,41	9475,59	4,57E-01	2019,80	996,00	4,77E-01	9089,59	12677,65	2,20E-01	2076,59	1209,34	3,54E-01	8248,98	7967,99	2,51E-01
SPBC28E12.03_b	5	1833,01	855,13	2179,83	1541,37	4,70E-01	7912,95	8599,28	7643,41	8364,13	6,62E-01	2435,50	1278,29	5,68E-01	9089,59	10297,45	1,74E-01	3169,41	1184,45	5,30E-01	8248,98	9152,82	5,15E-01
SPBC28F2.11_a	6	461,44	1351,18	548,75	2005,85	7,06E-01	28706,74	8599,28	26775,58	8364,13	9,44E-01	639,15	2091,03	6,44E-01	24516,95	10297,45	9,29E-01	714,11	2538,92	5,52E-01	24597,70	9152,82	9,01E-01
SPBC29A10.16c_b	8	837,53	439,59	910,17	278,20	9,16E-01	7357,20	29546,28	8604,47	27712,62	9,86E-01	744,43	436,55	8,66E-01	5752,90	26215,94	8,85E-01	739,29	621,67	8,59E-01	4965,61	24291,26	8,19E-01
SPBC2A9.08c_c	8	357,05	604,67	481,04	765,36	5,29E-01	17198,56	6702,47	20882,40	7601,58	8,12E-01	396,18	533,74	9,21E-01	19349,38	5527,27	9,60E-01	464,65	625,99	7,05E-01	16844,62	5033,45	9,10E-01
SPBC2D10.20_c	4	1136,20	280,14	903,89	319,57	8,70E-01	1876,91	15716,58	2031,48	18820,27	8,95E-01	1144,10	349,71	9,53E-01	1907,26	16844,62	9,60E-01	1499,22	427,57	7,46E-01	2002,41	15500,21	9,97E-01
SPBC2F12.05c_c	10	313,00	873,10	382,68	910,17	9,02E-01	10513,82	1682,93	10369,08	1766,22	9,96E-01	451,94	1234,75	6,55E-01	10884,59	1386,96	9,96E-01	464,65	1226,22	6,47E-01	10085,54	1614,83	9,71E-01
SPBC2F12.14c_a	15	652,58	326,29	625,99	498,00	7,19E-01	16231,28	11828,67	17600,77	12077,21	8,40E-01	576,03	776,05	4,32E-01	15183,54	14066,74	8,18E-01	837,53	837,53	1,66E-01	18209,13	12677,65	7,28E-01
SPBC2G2.08_a	11	1016,93	498,00	1243,34	652,58	6,76E-01	7538,18	14825,57	8079,22	16999,22	8,36E-01	2272,40	568,10	5,34E-01	5480,15	13931,08	8,16E-01	3468,27	1112,82	3,31E-01	7590,61	15690,30	9,41E-01
SPBC2G2.13c_c	8	326,29	770,69	461,44	1038,29	6,36E-01	8719,32	7486,11	9345,14	8902,53	2,59E-01	491,14	2091,03	4,65E-01	8248,98	5873,48	5,18E-01	451,94	2452,44	4,71E-01	8023,41	7590,61	6,95E-01
SPBC2G5.07c_a	4	448,82	390,72	592,22	413,00	4,72E-01	8526,02	8248,98	8520,82	8902,53	3,03E-01	588,13	458,25	2,83E-01	8198,28	7590,61	2,78E-01	491,14	467,88	1,96E-01	9084,29	6888,62	7,52E-01
SPBC2G5.07c_c	4	494,56	340,14	548,75	292,04	9,86E-01	8526,02	6945,91	8520,82	7678,51	7,24E-01	643,59	321,80	7,49E-01	8198,28	7512,33	9,02E-01	855,13	367,09	5,28E-01	9084,29	8938,87	2,49E-01

SPBC30D10.16_b	6	760,08	436,55	467,88	530,06	6,08E-01	8212,82	6945,91	7175,34	7678,51	8,44E-01	630,35	568,10	9,96E-01	6561,45	7512,33	5,64E-01	744,43	962,07	3,21E-01	7266,61	8938,87	6,67E-01
SPBC30D10.18c_a	12	2574,36	515,56	2418,67	526,39	9,63E-01	8101,05	7657,01	7474,11	6781,75	2,09E-01	2210,26	621,67	9,30E-01	4106,84	6123,32	1,16E-01	4451,27	734,19	6,71E-01	5886,53	6631,57	6,48E-02
SPBC31E1.04_a	4	256,00	2210,26	526,39	2469,49	8,65E-01	1176,27	6828,44	2076,59	6865,51	9,11E-01	313,00	2304,12	9,62E-01	1964,57	4023,74	7,69E-01	315,17	4640,29	6,52E-01	1438,15	5325,62	8,73E-01
SPBC31F10.06c_a	9	580,04	156,50	843,36	354,59	5,50E-01	33315,85	1243,34	33728,41	2149,82	9,79E-01	699,41	278,20	7,26E-01	30253,22	1807,78	9,59E-01	820,30	202,25	7,39E-01	31385,67	1278,29	9,70E-01
SPBC31F10.12_c	4	436,55	617,37	407,31	604,67	8,90E-01	2451,61	32880,72	3045,30	31273,87	9,83E-01	413,00	526,39	6,45E-01	2358,40	30868,16	9,64E-01	508,46	873,10	5,05E-01	2804,86	32255,36	9,95E-01
SPBC31F10.16_b	5	306,55	317,37	367,09	349,71	4,53E-02	4960,81	2261,33	5574,85	2654,72	8,24E-01	398,93	464,65	6,93E-02	5017,65	2102,65	9,82E-01	504,95	568,10	1,97E-02	4668,46	2428,26	9,75E-01
SPBC32F12.12c_a	4	1305,15	302,33	1562,89	308,69	8,84E-01	14359,29	4756,16	15396,51	5273,94	9,21E-01	1618,00	306,55	8,65E-01	14234,04	4471,07	9,79E-01	1530,73	372,22	8,65E-01	14638,07	4185,32	9,85E-01
SPBC32F12.12c_b	14	989,12	1296,13	1152,06	1323,37	6,43E-01	14359,29	12615,31	15396,51	13505,79	5,32E-01	1260,69	1418,35	3,72E-01	14234,04	13335,19	7,90E-01	1200,98	1595,73	4,14E-01	14638,07	13732,98	5,51E-01
SPBC32H8.05_a	4	212,31	820,30	154,34	1024,00	9,03E-01	2423,25	12615,31	2984,60	13505,79	9,30E-01	206,50	1045,52	8,52E-01	2464,87	13335,19	9,64E-01	224,41	1200,98	7,65E-01	3718,26	13732,98	8,81E-01
SPBC32H8.06_b	4	138,14	163,14	132,51	190,02	7,67E-01	63,70	2400,85	91,36	2497,67	9,74E-01	160,90	144,01	9,15E-01	91,95	2219,78	9,66E-01	181,02	259,57	2,33E-01	87,52	3138,45	8,61E-01
SPBC32H8.08c_b	9	1360,57	158,68	855,13	181,02	7,59E-01	3711,52	81,31	3375,56	103,08	9,55E-01	621,67	151,17	6,21E-01	2907,16	86,09	8,78E-01	873,10	151,17	7,58E-01	3586,65	83,10	9,83E-01
SPBC32H8.10_b	5	765,36	879,17	903,89	685,02	8,43E-01	15825,90	3282,58	14664,09	2951,57	9,39E-01	826,00	592,22	4,76E-01	14664,09	2630,13	9,26E-01	962,07	929,30	1,73E-01	15393,14	3372,31	9,86E-01
SPBC336.06c_a	7	639,15	564,18	617,37	776,05	3,92E-01	7136,32	15076,35	7597,11	13493,72	9,20E-01	714,11	608,87	4,52E-01	7247,87	13777,25	9,19E-01	1251,98	625,99	3,97E-01	8397,09	14868,79	9,27E-01
SPBC336.08_b	4	181,02	477,71	178,53	474,41	9,90E-01	846,57	6286,58	733,94	6118,96	9,74E-01	210,84	604,67	7,81E-01	1171,98	6691,93	9,33E-01	235,57	1082,39	5,39E-01	1415,37	7319,72	8,60E-01
SPBC336.14c_a	4	418,77	198,09	652,58	163,14	7,47E-01	4837,35	771,57	5367,37	856,33	9,29E-01	625,99	195,36	7,14E-01	4870,99	1126,60	9,50E-01	442,64	224,41	8,86E-01	4513,40	1296,90	9,73E-01
SPBC337.05c_a	12	433,53	415,87	481,04	809,00	3,12E-01	4317,82	4904,87	4545,58	4770,75	8,95E-01	385,34	552,56	6,51E-01	4195,79	4672,57	6,86E-01	541,19	494,56	6,47E-02	4253,98	4705,07	7,56E-01
SPBC337.09_a	4	227,54	393,44	174,85	396,18	8,73E-01	14155,33	4030,12	12650,77	4345,53	9,36E-01	171,25	451,94	9,95E-01	8898,94	4113,48	6,90E-01	222,86	445,72	8,80E-01	10236,09	4002,04	7,71E-01
SPBC337.12_a	8	803,41	190,02	724,08	171,25	9,16E-01	3373,08	12428,08	3557,56	13211,20	9,48E-01	754,83	176,07	9,48E-01	3139,56	9460,95	7,99E-01	749,61	235,57	9,93E-01	4025,50	8865,50	8,03E-01
SPBC354.01_b	4	254,23	448,82	317,37	560,28	6,31E-01	4192,91	2924,66	4775,18	3410,77	6,24E-01	250,73	744,43	6,37E-01	4273,49	2964,82	9,53E-01	290,02	809,00	5,49E-01	4461,22	3547,17	6,26E-01
SPBC354.02c_a	6	580,04	263,20	617,37	187,40	9,49E-01	20121,33	3773,61	19612,02	4129,94	9,95E-01	634,73	292,04	8,74E-01	17190,42	3975,04	9,09E-01	1260,69	439,59	4,33E-01	16024,77	3791,61	8,60E-01
SPBC354.02c_b	4	184,82	464,65	203,66	515,56	8,83E-01	20171,07	17719,35	19619,49	18716,00	8,80E-01	206,50	458,25	9,71E-01	17198,56	15914,67	2,26E-01	237,21	760,08	6,17E-01	16046,82	15258,00	1,25E-01
SPBC354.06_a	18	3125,78	143,01	3213,66	245,57	9,68E-01	19397,51	17682,08	20342,45	18690,27	4,98E-01	2574,36	167,73	9,03E-01	20052,25	15935,98	8,29E-01	3541,14	168,90	9,31E-01	18349,39	15286,81	4,30E-01
SPBC354.06_b	11	2418,67	2469,49	2574,36	2320,15	9,83E-01	19397,51	18986,49	20342,45	18704,03	7,33E-01	2556,58	2385,37	7,92E-01	20052,25	18900,09	6,88E-01	2957,17	2740,08	6,83E-02	18349,39	18641,13	1,10E-01
SPBC354.07c_b	23	849,22	1897,65	648,07	1858,60	8,95E-01	15563,59	18986,49	15377,26	18704,03	9,31E-01	685,02	2019,80	9,82E-01	14610,17	18900,09	8,67E-01	754,83	2452,44	8,39E-01	14080,12	18641,13	7,79E-01
SPBC354.07c_c	4	568,10	661,68	643,59	467,88	6,13E-01	15563,59	15064,02	15377,26	14314,50	5,09E-01	388,02	515,56	1,75E-01	14610,17	14115,54	1,14E-01	643,59	625,99	7,17E-01	14080,12	14600,25	1,14E-01
SPBC354.14c_b	5	765,36	512,00	634,73	367,09	5,33E-01	16787,24	15064,02	16131,45	14314,50	6,31E-01	588,13	477,71	5,24E-01	16037,43	14115,54	5,78E-01	588,13	724,08	9,15E-01	15070,45	14600,25	3,47E-01
SPBC354.14c_c	6	372,22	634,73	396,18	714,11	8,25E-01	16787,24	17436,94	16131,45	16680,55	2,39E-01	344,89	580,04	8,38E-01	16037,43	16398,84	1,38E-01	467,88	935,76	5,37E-01	15070,45	16355,69	1,91E-01
SPBC36.04_a	5	1478,58	413,00	2304,12	342,51	7,67E-01	7332,05	17436,94	8135,41	16680,55	9,97E-01	1978,24	433,53	8,08E-01	6793,79	16398,84	9,20E-01	3304,00	430,54	6,09E-01	6746,86	16355,69	9,16E-01
SPBC365.14c_a	7	694,58	1379,57	754,83	1833,01	7,26E-01	23623,34	6653,97	20923,47	7912,95	9,52E-01	657,11	1884,54	7,71E-01	18729,34	6562,36	8,34E-01	916,51	3304,00	4,79E-01	18271,93	5595,30	7,91E-01
SPBC3B8.04c_c	6	259,57	613,11	367,09	639,15	7,93E-01	2896,03	22244,39	2789,88	21054,53	9,66E-01	442,64	666,29	6,29E-01	2945,08	18123,86	8,84E-01	568,10	975,50	3,40E-01	2807,66	17407,68	8,58E-01

SPBC3B8.08_a	4	639,15	354,59	724,08	280,14	9,86E-01	6235,90	2985,28	6646,28	2857,65	9,60E-01	879,17	354,59	7,26E-01	7151,77	2807,50	9,04E-01	843,36	604,67	3,46E-01	8050,19	2570,24	8,47E-01
SPBC3B9.03_a	7	333,14	526,39	404,50	501,46	8,50E-01	3468,27	6075,61	3396,89	6552,27	9,30E-01	481,04	544,96	4,99E-01	3350,13	6823,28	8,98E-01	458,25	596,34	4,98E-01	3169,41	6926,55	9,15E-01
SPBC3B9.05_a	4	196,72	265,03	206,50	344,89	6,20E-01	838,86	3541,14	880,14	3281,18	9,57E-01	154,34	396,18	7,58E-01	762,63	3169,41	9,13E-01	222,86	377,41	4,98E-01	1006,10	3396,89	9,95E-01
SPBC3B9.10_c	4	152,22	230,72	163,14	263,20	7,65E-01	1629,68	923,70	2117,51	1094,46	6,49E-01	128,00	172,45	4,57E-01	1807,04	801,11	9,69E-01	124,50	191,34	5,82E-01	1488,88	779,45	8,03E-01
SPBC3B9.10_d	6	140,07	142,02	144,01	174,85	3,56E-01	1629,68	1562,38	2117,51	1927,71	5,15E-02	128,89	135,30	1,16E-01	1807,04	1625,93	3,39E-01	148,06	153,28	7,47E-02	1488,88	1436,23	8,90E-02
SPBC3B9.12_a	8	675,59	134,36	803,41	131,60	8,98E-01	12100,62	1562,38	11878,40	1927,71	9,93E-01	916,51	120,26	8,36E-01	13811,90	1625,93	9,22E-01	1089,92	135,30	7,42E-01	13298,57	1436,23	9,52E-01
SPBC3B9.13c_a	4	130,69	685,02	160,90	734,19	9,30E-01	3716,03	11480,71	4335,89	11820,29	9,37E-01	156,50	867,07	8,39E-01	3591,83	14743,68	8,39E-01	149,09	1341,84	6,59E-01	3688,48	14053,18	8,62E-01
SPBC3B9.13c_b	5	891,44	139,10	803,41	144,01	9,41E-01	3716,03	3057,53	4335,89	3851,32	2,26E-01	719,08	162,02	8,88E-01	3591,83	2631,80	6,83E-01	1287,18	168,90	7,82E-01	3688,48	3222,40	8,80E-01
SPBC3B9.14c_a	11	1807,78	837,53	1758,34	709,18	9,12E-01	21853,31	3057,53	21019,01	3851,32	9,99E-01	2091,03	749,61	9,17E-01	18614,22	2631,80	8,96E-01	2194,99	968,76	7,72E-01	19399,60	3222,40	9,35E-01
SPBC3B9.21_a	11	754,83	1509,65	1074,91	1458,23	7,81E-01	2368,90	21616,04	2469,49	19584,55	9,47E-01	1964,57	2033,85	1,49E-01	2435,50	18504,31	9,14E-01	1105,13	2574,36	4,82E-01	2469,49	20060,89	9,61E-01
SPBC3D6.05_b	13	380,04	792,35	354,59	861,08	9,53E-01	23110,67	2538,92	22766,99	2256,70	9,85E-01	337,79	1595,73	6,23E-01	22287,16	2469,49	9,78E-01	388,02	1448,15	6,19E-01	25647,95	2702,35	9,38E-01
SPBC3D6.06c_a	19	3350,13	335,46	3169,41	321,80	9,67E-01	18612,35	20753,79	19269,38	21586,09	6,83E-01	2977,74	315,17	9,31E-01	19828,05	18755,76	7,75E-01	3821,70	326,29	9,29E-01	21404,37	22374,08	2,01E-01
SPBC3D6.09_a	6	225,97	2556,58	224,41	2836,70	9,44E-01	5829,83	17173,37	5878,65	18787,66	9,32E-01	216,77	3104,19	8,98E-01	6841,20	18925,18	8,83E-01	238,86	3983,99	7,75E-01	6893,30	21748,68	7,91E-01
SPBC3E7.04c_f	10	734,19	205,07	648,07	171,25	8,82E-01	4082,53	5222,20	3601,81	5975,53	9,27E-01	765,36	187,40	9,88E-01	3810,86	6699,51	7,35E-01	867,07	243,88	8,53E-01	4648,69	6812,87	4,71E-01
SPBC3E7.07c_a	5	168,90	666,29	129,79	560,28	8,46E-01	2472,12	3750,30	2996,88	3221,52	9,98E-01	110,66	621,67	8,99E-01	2813,82	3568,65	9,24E-01	127,12	814,63	9,12E-01	3382,21	3814,29	5,45E-01
SPBC3E7.11c_a	4	427,57	131,60	455,09	127,12	9,63E-01	1251,18	3426,01	1387,15	3152,26	9,65E-01	515,56	130,69	8,74E-01	1275,21	3389,06	9,97E-01	526,39	149,09	8,31E-01	1330,45	3133,62	9,47E-01
SPBC3E7.14_a	4	306,55	388,02	401,71	439,59	2,44E-01	6888,62	985,73	6165,49	1093,11	9,44E-01	313,00	474,41	6,59E-01	4837,35	967,56	7,97E-01	290,02	544,96	6,52E-01	5595,30	1027,05	8,82E-01
SPBC3E7.15c_a	7	171,25	282,09	188,71	385,34	6,46E-01	7915,23	5914,33	8226,33	5404,70	9,59E-01	156,50	354,59	8,23E-01	7397,39	4153,18	6,11E-01	173,65	308,69	8,83E-01	6592,89	5955,47	6,04E-01
SPBC3F6.03_a	9	1710,26	179,77	1595,73	183,55	9,62E-01	31132,28	6811,55	32685,65	7433,55	9,56E-01	1541,37	173,65	9,40E-01	29831,19	6921,45	9,75E-01	1884,54	205,07	9,38E-01	27444,61	6553,26	9,13E-01
SPBC3H7.01_d	6	560,28	1448,15	560,28	1251,98	8,78E-01	4840,39	31433,55	5132,67	32447,99	9,76E-01	526,39	1370,04	9,35E-01	5374,55	29376,11	9,70E-01	826,00	1640,59	7,40E-01	4941,00	27154,78	9,15E-01
SPBC3H7.08c_b	6	162,02	544,96	182,28	448,82	8,86E-01	552,36	4431,96	409,24	4897,63	9,62E-01	167,73	519,15	9,73E-01	532,70	4904,89	9,45E-01	181,02	694,58	8,17E-01	875,02	4802,93	9,12E-01
SPBC3H7.15_a	5	955,43	198,09	1038,29	164,28	9,70E-01	30072,92	637,85	29377,08	611,13	9,88E-01	704,28	181,02	7,98E-01	26969,16	588,05	9,44E-01	1002,93	195,36	9,71E-01	26854,84	762,49	9,44E-01
SPBC4.01_a	4	685,02	873,10	515,56	843,36	6,51E-01	499,72	29183,45	746,76	28462,32	9,92E-01	481,04	922,88	7,79E-01	749,23	27659,29	9,77E-01	487,75	1468,37	7,29E-01	760,93	28726,10	9,97E-01
SPBC4.02c_b	10	1192,69	393,44	2256,70	477,71	6,16E-01	11746,96	598,22	11505,21	802,08	9,98E-01	2628,46	464,65	5,81E-01	11346,82	633,38	9,83E-01	3304,00	704,28	4,67E-01	12416,75	917,21	9,56E-01
SPBC4.04c_a	9	349,71	1168,14	421,68	1897,65	6,82E-01	7271,86	11910,94	7049,31	12161,22	9,97E-01	415,87	2272,40	6,22E-01	8396,28	11993,79	8,56E-01	552,56	3281,18	5,02E-01	8285,56	13969,57	7,16E-01
SPBC4.07c_a	5	300,25	364,56	259,57	393,44	9,44E-01	9557,52	7855,74	10382,65	7532,46	8,94E-01	319,57	458,25	5,37E-01	12319,36	8623,80	4,77E-01	240,52	556,41	7,22E-01	8575,22	8310,93	7,88E-01
SPBC409.03_b	7	1606,83	243,88	1992,00	227,54	8,84E-01	5556,65	9396,94	4513,40	10174,58	9,73E-01	2957,17	215,27	7,08E-01	4672,57	12044,35	8,52E-01	3565,78	282,09	6,31E-01	5220,60	8028,28	7,54E-01
SPBC409.06_a	6	2256,70	1499,22	2091,03	1897,65	7,94E-01	19332,67	4973,34	18616,25	4904,87	9,72E-01	2134,97	2836,70	3,60E-01	20054,69	5077,84	9,72E-01	3304,00	4359,66	9,51E-02	19727,02	5595,30	9,64E-01
SPBC409.12c_a	5	302,33	1937,53	385,34	1782,89	9,76E-01	1184,45	18615,60	989,12	17819,15	9,71E-01	556,41	1530,73	9,43E-01	1640,59	21223,61	9,18E-01	675,59	3396,89	6,22E-01	1884,54	21477,81	9,04E-01
SPBC409.13_a	11	347,29	326,29	487,75	421,68	7,66E-02	7804,01	1002,93	10884,59	929,30	8,26E-01	621,67	537,45	3,05E-02	14164,58	1360,57	6,89E-01	526,39	709,18	9,25E-02	8364,13	1734,13	9,04E-01

SPBC409.15_a	10	296,11	300,25	302,33	337,79	3,45E-01	2234,07	7858,29	3331,10	10884,59	7,04E-01	259,57	451,94	6,10E-01	3214,84	12330,98	6,61E-01	333,14	401,71	1,81E-01	3575,54	6653,97	9,85E-01
SPBC409.20c_a	18	526,39	190,02	643,59	245,57	7,72E-01	6198,26	2483,93	7343,49	3013,19	7,97E-01	533,74	276,28	8,46E-01	6468,85	2694,53	9,36E-01	675,59	268,73	7,08E-01	6360,76	3246,21	8,66E-01
SPBC409.23_a	15	278,20	481,04	313,00	548,75	7,73E-01	630,37	5257,89	1332,84	6621,92	7,96E-01	272,48	508,46	9,51E-01	1246,63	6037,22	8,53E-01	280,14	694,58	6,86E-01	960,79	5756,35	9,12E-01
SPBC428.06c_a	7	1002,93	210,84	1260,69	245,57	8,41E-01	5752,61	590,63	5792,62	1053,32	9,49E-01	1663,49	209,38	7,29E-01	6472,02	971,89	8,97E-01	1937,53	278,20	6,40E-01	6382,92	650,40	9,37E-01
SPBC428.12c_c	8	304,44	855,13	464,65	1152,06	6,55E-01	15076,35	5077,84	15181,22	5042,77	9,97E-01	337,79	1144,10	7,73E-01	14664,09	5256,91	9,88E-01	357,05	1530,73	6,31E-01	13587,57	5330,30	9,33E-01
SPBC428.19c_a	7	284,05	292,04	458,25	418,77	1,75E-02	10226,32	15716,58	10297,45	14766,09	9,12E-01	407,31	369,65	3,48E-02	8480,89	14972,21	7,97E-01	445,72	362,04	1,10E-01	9809,75	13400,51	7,17E-01
SPBC4C3.10c_c	8	243,88	313,00	300,25	421,68	3,59E-01	11268,44	11113,30	12161,22	9741,98	8,62E-01	268,73	347,29	6,29E-01	13400,51	8079,22	8,81E-01	347,29	424,61	1,74E-01	11746,96	10660,59	9,83E-01
SPBC4F6.04_a	11	704,28	225,97	584,07	313,00	9,57E-01	15133,03	10734,74	14515,93	11190,60	9,79E-01	464,65	259,57	7,30E-01	9039,11	12161,22	4,78E-01	916,51	308,69	7,40E-01	11273,11	11346,82	5,37E-01
SPBC4F6.15c_c	4	891,44	634,73	948,83	430,54	8,23E-01	4266,15	13845,87	4683,12	13771,70	9,82E-01	873,10	498,00	7,65E-01	4054,29	10671,65	7,99E-01	879,17	1038,29	3,25E-01	4288,00	12028,36	8,97E-01
SPBC530.04_a	10	484,38	770,69	1120,56	749,61	3,20E-01	12854,63	4101,42	13969,57	4427,20	9,22E-01	975,50	765,36	3,05E-01	15500,21	3749,38	8,90E-01	1176,27	729,11	3,45E-01	13587,57	4252,47	9,51E-01
SPBC530.06c_b	6	1031,12	584,07	1060,11	1060,11	3,76E-01	3077,15	12765,83	2627,92	13969,57	9,64E-01	897,64	1160,07	4,83E-01	3300,72	16158,44	8,43E-01	1260,69	1176,27	2,13E-01	3573,99	14066,74	9,11E-01
SPBC530.11c_b	4	2646,74	765,36	1144,10	831,75	5,30E-01	18584,66	3409,93	11905,35	2805,22	7,21E-01	1428,22	803,41	6,12E-01	13277,74	3463,36	7,99E-01	2019,80	1296,13	9,66E-01	19398,84	3899,88	9,58E-01
SPBC530.13_b	9	296,11	2503,97	219,79	1332,57	6,64E-01	3149,92	20892,44	2614,88	13516,50	7,41E-01	209,38	1606,83	7,43E-01	2825,45	15230,63	8,08E-01	321,80	2486,67	9,98E-01	4276,37	20612,62	9,75E-01
SPBC557.03c_a	7	372,22	249,00	477,71	215,27	8,28E-01	9877,98	2706,73	9877,98	2413,60	9,80E-01	491,14	200,85	8,43E-01	9946,68	2558,61	9,95E-01	600,49	296,11	4,90E-01	9809,75	3421,00	9,52E-01
SPBC56F2.10c_a	6	604,67	257,78	541,19	424,61	8,04E-01	628,48	10960,30	804,86	10660,59	9,94E-01	643,59	533,74	4,78E-01	829,56	10586,95	9,91E-01	661,68	634,73	3,39E-01	876,25	10226,32	9,75E-01
SPBC577.02_a	5	359,54	445,72	290,02	576,03	8,58E-01	4181,39	590,86	4265,95	729,29	9,69E-01	276,28	477,71	8,37E-01	3112,45	661,53	8,40E-01	342,51	544,96	7,45E-01	4937,00	544,97	9,12E-01
SPBC577.02_b	7	227,54	328,56	186,11	215,27	2,79E-01	4181,39	4677,90	4265,95	3018,91	3,62E-01	192,67	242,19	3,94E-01	3112,45	2877,26	3,48E-02	313,00	382,68	3,73E-01	4937,00	4806,39	2,27E-01
SPBC577.05c_b	6	172,45	240,52	176,07	162,02	3,94E-01	96,10	4677,90	107,70	3018,91	7,90E-01	142,02	209,38	5,86E-01	115,82	2877,26	7,71E-01	150,12	249,00	9,19E-01	94,27	4806,39	9,86E-01
SPBC577.11_a	7	522,76	155,42	704,28	166,57	7,95E-01	9170,69	101,41	7703,02	100,84	9,13E-01	608,87	183,55	8,58E-01	6762,62	84,89	8,50E-01	849,22	165,42	7,07E-01	10082,22	108,18	9,52E-01
SPBC577.11_b	5	157,59	596,34	176,07	436,55	8,08E-01	9170,69	8159,93	7703,02	6361,53	1,91E-01	198,09	512,00	9,43E-01	6762,62	5669,63	8,13E-02	194,01	903,89	7,20E-01	10082,22	9449,01	2,06E-01
SPBC582.05c_b	5	3258,52	190,02	3848,29	171,25	9,16E-01	6122,90	8159,93	6653,97	6361,53	6,01E-01	5792,62	173,65	7,32E-01	8902,53	5669,63	9,47E-01	5442,30	196,72	7,53E-01	8364,13	9449,01	2,66E-01
SPBC582.08_a	8	1002,93	2486,67	1234,75	3213,66	7,36E-01	19302,10	6793,79	20024,70	6746,86	9,74E-01	1045,52	4837,35	6,16E-01	16999,97	9607,86	9,75E-01	1269,46	4803,93	5,70E-01	16267,62	9345,14	9,76E-01
SPBC582.09_b	11	719,08	885,29	643,59	885,29	8,21E-01	2985,85	18302,55	2906,62	18808,63	9,86E-01	719,08	903,89	9,47E-01	2680,10	16319,65	9,21E-01	855,13	1243,34	3,63E-01	2535,48	16104,78	9,09E-01
SPBC609.01_a	5	268,73	512,00	352,14	552,56	7,32E-01	2019,80	2847,22	2469,49	2966,62	6,15E-01	261,38	680,29	7,71E-01	3125,78	2489,08	5,48E-01	300,25	826,00	6,11E-01	2740,08	2193,80	9,52E-01
SPBC646.11_c	5	471,14	276,28	530,06	385,34	5,60E-01	10449,18	2574,36	10512,52	2592,27	9,95E-01	484,38	247,28	9,64E-01	9027,03	3169,41	9,41E-01	709,18	265,03	6,86E-01	9184,53	3104,19	9,48E-01
SPBC649.02_a	7	831,75	310,83	709,18	385,34	9,45E-01	28741,39	9881,72	30725,02	9183,85	9,68E-01	739,29	315,17	9,08E-01	25135,64	8125,09	8,52E-01	831,75	584,07	6,82E-01	26995,54	8566,29	9,18E-01
SPBC649.04_b	9	3061,45	765,36	4067,71	608,87	8,57E-01	22693,63	27056,56	24833,50	26764,69	7,36E-01	2368,90	670,92	8,09E-01	23987,58	23629,40	6,74E-01	2778,33	903,89	9,66E-01	20738,16	23835,46	4,35E-01
SPBC651.01c_a	10	4182,07	2320,15	3691,52	3326,99	8,11E-01	16729,19	22073,07	16611,36	24661,96	8,22E-01	3666,02	2149,82	8,02E-01	14519,12	26249,46	8,93E-01	4576,41	3281,18	6,11E-01	18742,73	23493,92	6,78E-01
SPBC660.08_a	4	1089,92	3565,78	3743,05	2817,11	5,46E-01	2574,36	19450,09	3104,19	18722,95	9,94E-01	2241,11	3304,00	7,73E-01	2998,45	17306,86	9,45E-01	1618,00	5184,54	6,70E-01	2610,30	21037,26	9,54E-01
SPBC660.11_a	10	433,53	1389,16	584,07	4938,99	4,94E-01	31651,80	2876,30	31433,17	3640,70	9,90E-01	661,68	2817,11	5,55E-01	30786,28	3590,58	9,97E-01	484,38	1897,65	7,74E-01	24154,43	2936,74	8,54E-01

SPBC660.15_c	13	380,04	364,56	401,71	515,56	2,72E-01	6329,61	30786,28	6766,82	30786,28	9,91E-01	421,68	670,92	2,98E-01	6657,55	32995,92	9,50E-01	491,14	596,34	8,42E-02	7644,53	25355,30	9,04E-01
SPBC660.16_b	6	1520,15	415,87	1351,18	367,09	8,96E-01	26655,28	6515,96	28354,96	7508,25	9,34E-01	1408,55	369,65	9,27E-01	29290,72	6442,53	9,41E-01	1278,29	413,00	8,78E-01	18954,02	7010,35	7,87E-01
SPBC660.16_c	36	2048,00	1200,98	2418,67	1398,83	7,10E-01	26655,28	26761,75	28354,96	29208,13	4,04E-02	2368,90	1269,46	8,05E-01	29290,72	30127,79	1,92E-02	1820,35	1038,29	7,67E-01	18954,02	18690,07	3,25E-04
SPBC660.16_d	6	393,44	1897,65	617,37	2336,28	7,99E-01	26615,89	26761,75	28329,16	29208,13	4,29E-02	604,67	1937,53	9,12E-01	29328,18	30127,79	1,74E-02	694,58	1468,37	9,47E-01	18951,18	18690,07	3,61E-04
SPBC685.02_b	6	276,28	415,87	292,04	760,08	5,38E-01	609,05	26801,01	539,30	29125,60	9,59E-01	413,00	675,59	3,14E-01	714,44	30152,71	9,38E-01	401,71	648,07	3,34E-01	712,48	18690,27	8,25E-01
SPBC685.02_c	5	137,19	202,25	173,65	206,50	6,33E-01	608,87	738,93	537,45	579,33	2,33E-01	156,50	272,48	5,70E-01	714,11	735,32	5,21E-01	194,01	388,02	3,58E-01	714,11	658,57	8,77E-01
SPBC685.05_a	7	380,04	129,79	321,80	163,14	9,41E-01	6256,02	739,29	4333,29	580,04	7,85E-01	374,81	176,07	9,10E-01	3861,98	734,19	7,42E-01	494,56	168,90	7,44E-01	5066,69	657,11	8,74E-01
SPBC691.05c_a	5	354,59	362,04	335,46	330,84	2,90E-02	9750,65	5875,33	8722,57	4281,79	7,00E-01	421,68	317,37	8,50E-01	7643,07	4003,36	5,32E-01	415,87	458,25	6,72E-02	7332,42	4912,58	5,36E-01
SPBC713.02c_a	4	321,80	357,05	261,38	349,71	5,50E-01	10593,56	9126,86	12182,46	8170,56	8,96E-01	364,56	330,84	7,67E-01	12611,42	7983,30	8,74E-01	304,44	430,54	7,10E-01	9433,75	7807,15	3,75E-01
SPBC713.11c_a	15	6122,90	237,21	4451,27	222,86	8,38E-01	22678,38	14521,97	22423,12	15300,87	9,66E-01	4938,99	240,52	8,90E-01	22529,97	16549,48	8,70E-01	5634,22	245,57	9,57E-01	22672,98	13276,87	9,29E-01
SPBC725.10_a	21	1009,90	5442,30	1323,37	2977,74	6,94E-01	13034,07	19711,60	11910,94	19146,75	8,80E-01	1428,22	4544,80	9,38E-01	28526,20	19340,48	3,15E-01	935,76	4938,99	9,32E-01	14462,21	20548,65	8,25E-01
SPBC725.16_a	4	989,12	849,22	955,43	1428,22	3,84E-01	10982,71	12765,83	10967,80	11505,21	5,64E-01	770,69	1408,55	6,54E-01	11212,43	25888,07	4,62E-01	797,86	1128,35	8,29E-01	10675,78	14263,10	7,94E-01
SPBC725.17c_a	4	218,27	797,86	304,44	873,10	8,61E-01	396,21	11255,68	355,55	11124,46	9,92E-01	230,72	699,41	9,19E-01	466,99	12015,15	9,63E-01	243,88	1128,35	7,68E-01	577,56	11406,13	9,85E-01
SPBC776.02c_a	17	604,67	252,48	910,17	238,86	7,37E-01	26615,89	302,78	26432,04	356,37	9,98E-01	826,00	215,27	8,18E-01	23493,92	285,02	9,37E-01	867,07	250,73	7,49E-01	23010,42	458,21	9,30E-01
SPBC776.04_c	7	436,55	458,25	519,15	608,87	1,27E-01	9098,00	23493,92	9976,61	23987,58	9,52E-01	487,75	621,67	2,54E-01	8163,76	23170,48	9,57E-01	424,61	765,36	4,78E-01	8592,25	21920,61	9,25E-01
SPBC776.15c_b	13	903,89	413,00	989,12	477,71	8,52E-01	6659,35	9375,21	6966,90	10522,17	7,76E-01	903,89	487,75	9,18E-01	6756,52	8347,64	7,95E-01	1448,15	560,28	5,66E-01	6491,12	8664,06	8,24E-01
SPBC800.04c_a	4	1323,37	792,35	1618,00	968,76	6,31E-01	55850,72	6192,39	55766,04	7039,69	9,92E-01	1782,89	826,00	6,96E-01	54788,48	6276,74	9,90E-01	1833,01	1226,22	3,63E-01	55683,57	5530,85	9,92E-01
SPBC800.08_a	6	147,03	1136,20	141,04	1360,57	9,02E-01	1736,57	54672,84	1774,96	54704,90	9,99E-01	137,19	1060,11	9,55E-01	1535,69	53903,38	9,91E-01	173,65	2164,77	6,82E-01	1739,95	55653,53	9,91E-01
SPBC800.09_c	4	1209,34	146,02	1152,06	154,34	9,76E-01	17987,55	1303,71	18197,74	1414,96	9,90E-01	1152,06	155,42	9,77E-01	16598,16	1152,14	9,52E-01	1675,06	154,34	8,22E-01	17178,63	1306,02	9,75E-01
SPBC83.01_a	6	284,05	968,76	290,02	1209,34	8,50E-01	6508,95	15681,55	6162,43	16760,51	9,63E-01	254,23	1226,22	8,66E-01	6440,68	15225,05	9,71E-01	344,89	1833,01	6,29E-01	6374,58	14351,65	9,15E-01
SPBC83.08_a	4	222,86	230,72	168,90	233,94	5,20E-01	5626,44	6398,50	5968,07	7289,55	5,05E-01	250,73	240,52	9,98E-02	5085,42	7068,07	9,57E-01	268,73	310,83	9,88E-02	5618,95	6485,75	9,51E-01
SPBC83.14c_a	6	362,04	229,13	458,25	270,60	6,10E-01	6048,83	5398,75	6197,96	5358,25	9,28E-01	504,95	243,88	6,44E-01	5199,90	4933,70	2,02E-01	484,38	280,14	5,51E-01	6429,70	5422,84	7,68E-01
SPBC83.15_b	6	313,00	385,34	326,29	382,68	9,18E-01	3400,17	5810,57	3893,87	5907,31	8,68E-01	294,07	404,50	9,99E-01	3429,65	5037,80	8,22E-01	398,93	568,10	2,82E-01	4335,66	5596,72	8,16E-01
SPBC83.15_d	4	133,44	276,28	184,82	242,19	9,21E-01	3396,89	3109,40	3902,01	3501,35	2,11E-01	181,02	232,32	9,83E-01	3420,52	2865,83	7,58E-01	219,79	372,22	4,75E-01	4329,55	3351,65	3,68E-01
SPBC839.02_a	5	315,17	129,79	315,17	171,25	8,76E-01	9766,19	3104,19	8400,17	3492,39	9,17E-01	324,03	151,17	9,16E-01	8668,60	2856,44	8,93E-01	310,83	199,47	7,91E-01	8940,07	3350,13	9,53E-01
SPBC839.05c_a	12	1820,35	202,25	1243,34	272,48	8,13E-01	20228,85	10609,67	18718,01	9049,58	8,43E-01	1097,50	304,44	7,63E-01	14545,83	8854,63	5,74E-01	2005,85	225,97	9,39E-01	16393,52	9272,74	7,08E-01
SPBC839.09c_a	4	352,14	1296,13	533,74	879,17	8,37E-01	18432,96	18806,99	21618,82	18518,03	4,52E-01	564,18	948,83	9,07E-01	20171,07	14883,76	7,20E-01	885,29	1573,76	5,59E-01	18053,61	14445,58	3,21E-01
SPBC839.16_b	12	1686,71	330,84	1160,07	439,59	8,11E-01	19663,56	19215,73	19379,14	19483,97	9,75E-01	1389,16	451,94	9,25E-01	12005,68	18951,18	3,73E-01	2856,44	1120,56	4,68E-01	15498,24	22226,61	8,80E-01
SPBC839.17c_b	4	213,78	1217,75	359,54	1217,75	9,22E-01	9809,75	23386,41	11665,82	22371,69	9,66E-01	404,50	1097,50	9,59E-01	14362,31	15249,92	8,17E-01	342,51	2320,15	6,35E-01	11665,82	19024,10	8,86E-01
SPBC839.17c_c	5	171,25	278,20	216,77	410,15	5,06E-01	9809,75	7281,40	11665,82	10369,08	2,24E-01	247,28	274,37	5,80E-01	14362,31	12590,08	8,56E-02	221,32	306,55	6,24E-01	11665,82	8841,04	4,63E-01

SPBC839.17c_d	8	1458,23	210,84	1978,24	304,44	7,96E-01	9809,75	7281,40	11665,82	10369,08	2,24E-01	1992,00	222,86	8,24E-01	14362,31	12590,08	8,56E-02	2836,70	212,31	6,82E-01	11665,82	8841,04	4,63E-01
SPBC839.19_a	5	209,38	1112,82	199,47	1296,13	9,14E-01	1836,75	7281,40	1592,11	10369,08	8,09E-01	156,50	1332,57	9,21E-01	1643,55	12590,08	7,16E-01	209,38	1686,71	7,72E-01	2578,39	8841,04	8,08E-01
SPBC887.02_a	4	280,14	174,85	340,14	191,34	7,16E-01	9975,63	1871,29	8965,87	1659,74	9,21E-01	302,33	177,29	8,94E-01	9131,75	1298,18	9,11E-01	298,17	225,97	6,42E-01	9011,64	2233,12	9,60E-01
SPBC8D2.01_b	23	7281,40	272,48	7383,04	235,57	9,95E-01	9336,90	9832,24	9055,76	8910,06	1,45E-01	6562,36	224,41	9,43E-01	8834,12	9150,65	1,82E-01	6888,62	268,73	9,71E-01	8716,31	9313,79	2,80E-01
SPBC8D2.02c_a	5	982,29	6122,90	1152,06	5996,90	9,96E-01	14065,95	9251,29	13733,87	8466,10	8,90E-01	1314,23	5330,30	9,50E-01	12406,92	8673,46	7,49E-01	2486,67	6793,79	7,76E-01	13308,72	9124,00	9,02E-01
SPBC8D2.07c_a	7	2272,40	1024,00	2385,37	1067,48	9,39E-01	12093,79	13710,73	10912,20	13065,70	5,68E-01	2740,08	1323,37	7,24E-01	10899,72	13102,84	5,77E-01	3350,13	2048,00	3,64E-01	10870,85	13028,69	5,53E-01
SPBC8D2.10c_b	14	1458,23	2486,67	1573,76	2759,13	8,28E-01	8636,91	12223,03	9175,65	10961,58	8,73E-01	2469,49	3468,27	2,99E-01	7340,60	11225,22	7,07E-01	3191,46	4837,35	1,70E-01	7381,18	11748,52	7,88E-01
SPBC8D2.12c_b	14	173,65	1226,22	186,11	1305,15	9,58E-01	5369,97	7696,03	4750,57	7917,41	9,29E-01	194,01	1509,65	8,74E-01	5627,22	6398,67	7,13E-01	200,85	3147,52	5,97E-01	6988,80	6773,26	7,94E-01
SPBC8D2.19_b	6	315,17	162,02	328,56	188,71	8,65E-01	147,82	5114,75	166,07	3997,46	8,77E-01	342,51	178,53	8,63E-01	225,55	4479,22	9,40E-01	254,23	179,77	8,23E-01	170,75	6320,42	8,91E-01
SPBC902.05c_b	6	584,07	268,73	814,63	276,28	7,39E-01	17198,56	168,04	18179,19	180,47	9,72E-01	916,51	284,05	6,71E-01	18179,19	208,00	9,71E-01	975,50	252,48	6,81E-01	16270,83	168,83	9,72E-01
SPBC902.05c_d	4	205,07	699,41	229,13	968,76	7,73E-01	17205,35	17559,94	18178,47	18053,61	5,98E-02	233,94	849,22	8,42E-01	18140,15	18561,17	7,22E-02	219,79	1089,92	7,25E-01	16280,33	17079,76	2,49E-01
SPBC947.03c_a	5	2469,49	216,77	2149,82	235,57	9,28E-01	1534,48	17530,39	1442,54	18015,70	9,88E-01	2856,44	176,07	9,30E-01	1550,48	18571,43	9,68E-01	5293,48	194,01	6,65E-01	1780,00	17044,48	9,92E-01
SPBC9B6.07_c	7	213,78	2401,97	268,73	1675,06	8,20E-01	1752,03	1511,51	1809,15	1255,14	7,73E-01	263,20	2628,46	9,40E-01	1651,03	1333,36	5,56E-01	261,38	5752,61	6,23E-01	1965,91	1600,31	5,61E-01
SPBC9B6.10_b	14	617,37	222,86	831,75	207,94	8,12E-01	6793,79	1593,88	7858,29	1819,39	8,86E-01	1089,92	218,27	6,73E-01	10226,32	1512,06	7,73E-01	621,67	261,38	9,43E-01	5873,48	1727,21	9,17E-01
SPBC9B6.11c_a	4	491,14	464,65	448,82	608,87	5,94E-01	10679,77	6608,01	11851,52	7590,61	7,50E-01	390,72	826,00	6,10E-01	9356,04	9809,75	6,92E-01	630,35	560,28	8,85E-02	9591,49	5832,91	7,69E-01
SPBP16F5.04_a	17	259,57	448,82	337,79	487,75	6,75E-01	5148,73	9886,59	9809,75	10075,20	4,14E-01	377,41	600,49	4,54E-01	13307,94	8356,89	4,35E-01	344,89	592,22	5,39E-01	7131,55	9015,21	8,48E-01
SPBP22H7.06_c	6	1871,53	280,14	1782,89	349,71	9,94E-01	11928,97	4803,93	12636,25	10441,20	4,84E-01	1951,00	374,81	9,45E-01	10295,93	12677,65	4,94E-01	3848,29	333,14	6,51E-01	13013,71	6841,04	7,72E-01
SPBP23A10.03c_a	4	170,07	1833,01	163,14	1618,00	9,29E-01	1757,12	11421,23	1484,19	11880,24	9,91E-01	156,50	1951,00	9,70E-01	1612,12	9169,47	8,63E-01	196,72	3396,89	7,02E-01	1739,85	12504,84	9,48E-01
SPBP23A10.05_b	4	418,77	159,79	596,34	170,07	7,43E-01	6562,36	2029,35	7082,29	1234,38	9,74E-01	471,14	167,73	8,94E-01	6888,62	1432,67	9,73E-01	576,03	212,31	6,85E-01	6793,79	2099,90	9,67E-01
SPBP23A10.10_a	6	680,29	464,65	843,36	643,59	3,65E-01	4913,55	5792,62	4975,55	6038,61	8,44E-01	498,00	661,68	9,62E-01	4046,53	5792,62	7,01E-01	867,07	494,56	6,65E-01	4723,65	5914,33	9,67E-01
SPBP23A10.14c_a	4	335,46	576,03	404,50	643,59	7,26E-01	12503,67	5112,57	11877,02	5030,21	9,50E-01	390,72	471,14	8,63E-01	12243,62	4220,13	9,26E-01	533,74	770,69	3,65E-01	11253,27	4753,96	8,85E-01
SPBP35G2.04c_a	7	1278,29	418,77	1323,37	421,68	9,73E-01	5762,48	11739,24	5491,50	11292,60	9,39E-01	1144,10	501,46	9,66E-01	5758,37	12822,11	9,18E-01	1024,00	512,00	8,87E-01	5805,40	10838,55	9,23E-01
SPBP4H10.04_b	7	498,00	1243,34	621,67	891,44	8,01E-01	4970,95	6220,46	5252,48	4891,17	5,05E-01	685,02	1120,56	9,47E-01	4873,26	5168,45	4,65E-01	975,50	1341,84	5,60E-01	4388,72	6776,15	9,93E-01
SPBP4H10.12_a	5	1251,98	467,88	1379,57	458,25	9,31E-01	10791,99	4709,31	12995,09	4801,86	8,43E-01	1428,22	461,44	9,04E-01	16154,28	4778,82	7,15E-01	1226,22	955,43	6,34E-01	13390,82	4307,43	8,59E-01
SPBP4H10.20_b	5	714,11	1097,50	541,19	1002,93	6,99E-01	6053,57	9791,90	6419,72	12593,78	7,04E-01	843,36	1606,83	5,33E-01	4991,58	13262,50	8,16E-01	996,00	1243,34	4,47E-01	4355,09	10555,78	9,09E-01
SPBP8B7.07c_a	5	280,14	729,11	270,60	670,92	9,21E-01	2531,58	5318,72	2206,45	5713,28	9,89E-01	302,33	666,29	9,50E-01	2319,21	4624,66	8,26E-01	321,80	776,05	9,02E-01	2430,19	4467,94	8,09E-01
SPBP8B7.10c_a	5	270,60	354,59	252,48	272,48	3,66E-01	1313,31	2085,73	1476,97	1940,95	9,85E-01	247,28	317,37	6,36E-01	1314,67	1787,91	7,75E-01	362,04	313,00	6,59E-01	1568,12	1753,64	9,31E-01
SPBP8B7.11_b	5	451,94	199,47	580,04	232,32	7,44E-01	20729,24	1322,96	19790,53	1264,77	9,74E-01	592,22	209,38	7,74E-01	16078,23	1269,66	8,65E-01	512,00	333,14	5,95E-01	15853,85	1803,16	8,71E-01
SPBP8B7.16c_a	31	1192,69	369,65	962,07	324,03	8,16E-01	13314,64	18080,62	16389,19	19094,63	5,33E-01	1278,29	421,68	9,18E-01	12582,52	15336,07	5,92E-01	1478,58	445,72	8,10E-01	13373,28	14730,05	5,75E-01
SPBP8B7.24c_a	5	206,50	1082,39	218,27	843,36	8,52E-01	605,74	12516,24	586,17	15832,06	8,80E-01	199,47	1024,00	9,62E-01	527,89	11884,86	9,70E-01	237,21	1341,84	8,56E-01	452,76	12217,05	9,81E-01

SPCC1020.03_b	9	265,03	199,47	198,09	232,32	6,90E-01	10964,14	351,42	7804,84	488,99	8,36E-01	215,27	235,57	8,61E-01	3103,60	477,18	5,53E-01	222,86	227,54	8,50E-01	4631,56	337,57	6,35E-01
SPCC1020.04c_a	7	390,72	302,33	504,95	238,86	8,73E-01	13684,38	11244,96	13525,68	8556,08	6,58E-01	494,56	212,31	9,67E-01	12987,81	3566,57	4,80E-01	814,63	218,27	6,30E-01	15454,35	4168,05	6,91E-01
SPCC1020.07_c	7	910,17	367,09	955,43	382,68	9,46E-01	12866,41	13406,34	12501,28	12734,93	2,20E-01	861,08	308,69	9,02E-01	8711,21	11563,99	1,75E-01	948,83	625,99	6,84E-01	11018,44	14075,87	7,41E-01
SPCC1183.02_a	10	613,11	885,29	873,10	719,08	7,93E-01	12503,12	11998,90	12416,75	12304,81	7,12E-01	1128,35	837,53	3,61E-01	10015,87	8782,07	5,05E-02	1686,71	1160,07	1,51E-01	9026,81	10049,53	4,14E-02
SPCC1183.03c_a	6	515,56	608,87	685,02	675,59	1,28E-01	1992,00	10960,30	1951,00	10660,59	9,81E-01	809,00	968,76	7,17E-02	1858,60	9607,86	9,12E-01	996,00	1418,35	9,64E-02	2401,97	7590,61	8,02E-01
SPCC1183.10_a	12	280,14	544,96	265,03	617,37	9,08E-01	638,19	2033,85	538,27	1978,24	9,45E-01	270,60	734,19	7,68E-01	618,46	1722,16	8,69E-01	377,41	1120,56	4,84E-01	942,09	2452,44	7,59E-01
SPCC1223.08c_a	8	367,09	250,73	433,53	288,01	6,34E-01	8192,00	619,88	8248,98	632,85	9,95E-01	508,46	377,41	2,66E-01	7434,40	704,00	9,53E-01	560,28	398,93	2,28E-01	9475,59	864,31	9,06E-01
SPCC1223.10c_a	5	481,04	319,57	670,92	401,71	4,78E-01	9216,48	7082,29	11036,54	7538,18	6,34E-01	410,15	439,59	7,93E-01	12503,12	6936,54	6,51E-01	588,13	560,28	1,68E-01	11346,82	8364,13	4,50E-01
SPCC1223.10c_b	6	481,04	359,54	719,08	484,38	3,03E-01	9216,48	9280,58	11036,54	10226,32	7,66E-02	576,03	564,18	1,34E-01	12503,12	11505,21	3,14E-02	903,89	541,19	2,55E-01	11346,82	11190,60	1,74E-03
SPCC1223.10c_c	4	512,00	344,89	776,05	600,49	1,65E-01	9216,48	9280,58	11036,54	10226,32	7,66E-02	680,29	448,82	4,41E-01	12503,12	11505,21	3,14E-02	704,28	786,88	7,66E-02	11346,82	11190,60	1,74E-03
SPCC1235.07_a	6	232,32	451,94	249,00	544,96	7,94E-01	1078,06	9280,58	913,01	10226,32	9,56E-01	232,32	661,68	7,06E-01	733,54	11505,21	9,02E-01	319,57	935,76	4,75E-01	1133,30	11190,60	8,94E-01
SPCC1235.07_c	4	212,31	200,85	230,72	229,13	5,62E-02	1078,06	935,87	913,01	850,61	2,48E-01	174,85	172,45	3,02E-02	733,54	789,98	8,51E-02	225,97	278,20	2,31E-01	1133,30	1165,41	1,90E-01
SPCC1235.11_c	7	2721,15	198,09	5113,16	215,27	7,05E-01	12330,98	935,87	14164,58	850,61	9,30E-01	3769,09	250,73	8,23E-01	12854,63	789,98	9,84E-01	4904,87	224,41	7,18E-01	16728,26	1165,41	8,33E-01
SPCC1235.15_a	10	781,44	2210,26	689,78	3795,30	7,05E-01	6218,59	9877,98	6456,63	12077,21	7,51E-01	604,67	3350,13	7,85E-01	5867,63	10015,87	9,73E-01	588,13	4672,57	6,52E-01	5726,96	14868,79	6,93E-01
SPCC1235.15_b	4	814,63	648,07	916,51	556,41	9,82E-01	6218,59	5383,87	6456,63	5654,77	7,03E-01	786,88	666,29	9,67E-01	5867,63	5258,65	6,90E-01	935,76	754,83	4,52E-01	5726,96	5506,14	7,10E-01
SPCC1235.16_a	6	276,28	760,08	393,44	760,08	8,65E-01	388,70	5383,87	919,78	5654,77	9,18E-01	333,14	781,44	9,16E-01	867,23	5258,65	9,62E-01	369,65	1200,98	6,34E-01	534,15	5506,14	9,73E-01
SPCC1259.03_a	11	294,07	298,17	333,14	263,20	9,59E-01	9608,39	327,32	11391,76	581,94	8,99E-01	302,33	284,05	7,84E-01	9501,35	588,31	9,92E-01	491,14	330,84	2,88E-01	11943,58	304,95	8,91E-01
SPCC1259.15c_b	7	319,57	205,07	274,37	265,03	9,10E-01	11203,47	8178,52	9487,73	9735,77	9,63E-01	304,44	252,48	8,21E-01	9437,71	7040,25	5,30E-01	354,59	335,46	2,90E-01	9918,85	9319,22	9,67E-01
SPCC126.05c_b	6	147,03	296,11	200,85	294,07	7,96E-01	4532,64	8115,99	4809,57	7614,31	9,65E-01	153,28	268,73	9,21E-01	4881,06	8531,74	8,95E-01	207,94	261,38	8,84E-01	4996,85	8618,60	8,67E-01
SPCC126.05c_c	4	176,07	179,77	153,28	183,55	5,97E-01	4532,64	4453,60	4809,57	4403,09	6,39E-01	164,28	177,29	4,02E-01	4881,06	4064,97	9,65E-01	159,79	187,40	7,86E-01	4996,85	4463,21	4,72E-01
SPCC126.06_c	4	1074,91	179,77	1379,57	230,72	8,30E-01	20088,70	4453,60	18216,13	4403,09	9,35E-01	2194,99	158,68	6,70E-01	17667,38	4064,97	9,05E-01	2418,67	179,77	6,33E-01	16786,50	4463,21	8,84E-01
SPCC126.09_a	22	3019,30	1217,75	2048,00	1038,29	6,33E-01	22005,57	18327,03	19293,01	17313,35	4,67E-01	2194,99	1833,01	9,20E-01	18314,63	17267,05	3,40E-01	2856,44	2610,30	5,69E-01	21075,72	17893,02	8,06E-01
SPCC126.13c_a	6	270,60	2646,74	390,72	1629,26	7,70E-01	6208,38	22263,70	7967,99	17892,52	9,03E-01	342,51	1618,00	7,57E-01	8779,97	16978,16	8,94E-01	270,60	2977,74	9,35E-01	7912,95	22037,56	9,51E-01
SPCC1281.08_a	6	280,14	247,28	263,20	319,57	4,86E-01	583,87	6251,56	651,02	8422,31	8,38E-01	266,87	259,57	9,80E-01	921,06	8599,28	8,05E-01	268,73	292,04	4,95E-01	717,83	8719,32	8,16E-01
SPCC132.03_a	8	2033,85	250,73	1488,87	245,57	8,24E-01	8030,00	623,39	7412,04	723,09	9,63E-01	2336,28	259,57	9,20E-01	6531,23	748,55	8,97E-01	2610,30	243,88	8,65E-01	7361,80	644,33	9,54E-01
SPCC1322.08_a	8	17438,64	1379,57	9089,59	1389,16	6,86E-01	11117,09	7408,17	12290,93	6585,88	9,63E-01	7486,11	2272,40	6,45E-01	8488,24	6749,35	5,06E-01	11036,54	2836,70	8,10E-01	7956,23	6996,34	4,50E-01
SPCC1322.09_b	6	212,31	17559,94	221,32	8719,32	6,92E-01	6399,11	11464,42	6365,24	11830,94	9,68E-01	230,72	9674,69	7,29E-01	6957,04	8387,66	6,79E-01	245,57	13124,73	8,57E-01	5151,98	8764,53	5,91E-01
SPCC1322.11_a	11	3169,41	225,97	2998,45	200,85	9,66E-01	52597,87	7038,56	53789,38	6145,67	9,97E-01	2702,35	191,34	9,09E-01	48983,67	6933,16	9,58E-01	3983,99	265,03	8,74E-01	50935,56	5929,91	9,69E-01
SPCC1322.16_a	8	212,31	2503,97	240,52	2272,40	9,53E-01	1583,51	51955,26	2101,52	52430,00	9,90E-01	194,01	2076,59	8,94E-01	3287,38	51412,57	9,88E-01	247,28	3326,99	8,44E-01	2383,70	50650,48	9,95E-01
SPCC1393.09c_a	6	210,84	216,77	191,34	249,00	8,46E-01	12039,70	1448,89	10655,00	2066,27	9,60E-01	210,84	196,72	3,21E-01	8413,88	2569,49	8,55E-01	276,28	237,21	1,62E-01	10914,07	1948,18	9,68E-01

SPCC1393.09c_c	6	155,42	181,02	140,07	177,29	7,14E-01	12039,70	13352,71	10655,00	10607,21	8,80E-02	146,02	176,07	7,51E-01	8413,88	8230,06	2,22E-02	141,04	310,83	5,71E-01	10914,07	14344,77	9,74E-01
SPCC1393.13_a	4	268,73	148,06	661,68	122,79	5,74E-01	14263,10	13352,71	15825,90	10607,21	8,44E-01	625,99	176,07	4,95E-01	18432,96	8230,06	9,34E-01	776,05	152,22	5,05E-01	14164,58	14344,77	4,37E-01
SPCC13B11.03c_b	7	393,44	266,87	357,05	436,55	4,67E-01	280,51	12416,75	283,37	15181,22	8,99E-01	396,18	519,15	2,85E-01	379,81	17318,18	8,33E-01	427,57	630,35	2,38E-01	569,87	11425,74	9,70E-01
SPCC1442.03_a	4	552,56	484,38	576,03	410,15	8,04E-01	13669,84	299,59	12995,11	279,18	9,73E-01	625,99	430,54	9,33E-01	13232,04	331,98	9,85E-01	661,68	474,41	6,68E-01	14765,03	434,34	9,56E-01
SPCC1442.06_c	6	129,79	487,75	137,19	689,78	7,81E-01	18050,87	13722,28	18817,41	12786,00	9,84E-01	133,44	760,08	7,39E-01	19806,36	13529,27	8,57E-01	138,14	749,61	7,40E-01	17533,90	16734,28	6,28E-01
SPCC1442.09_a	7	424,61	123,64	464,65	153,28	8,87E-01	2664,83	16047,12	2772,64	15732,72	9,92E-01	398,93	101,13	9,20E-01	2072,59	16838,64	9,93E-01	504,95	117,78	8,93E-01	2731,21	16264,37	9,89E-01
SPCC1442.12_b	11	2740,08	374,81	2469,49	372,22	9,39E-01	18833,69	2343,93	17844,34	2404,70	9,71E-01	1552,09	362,04	6,95E-01	16935,50	1793,20	9,23E-01	1152,06	487,75	6,09E-01	15737,98	2501,82	9,02E-01
SPCC1442.19_a	7	1833,01	1746,20	1698,45	1795,29	5,79E-01	12076,13	16079,66	11564,54	15559,07	8,72E-01	2556,58	1052,79	9,86E-01	10674,58	14423,31	6,33E-01	3640,70	837,53	7,79E-01	11641,45	14398,01	7,06E-01
SPCC1450.04_a	10	1418,35	1408,55	1478,58	1618,00	1,93E-01	25056,25	10705,92	28157,48	10707,61	9,03E-01	1964,57	1978,24	2,27E-04	24801,38	10913,55	9,98E-01	1722,16	3147,52	2,88E-01	25735,86	11751,16	9,39E-01
SPCC1450.08c_e	12	313,00	1192,69	335,46	1217,75	9,73E-01	883,42	23648,70	589,75	26430,16	9,49E-01	352,14	1871,53	7,22E-01	702,83	23951,26	9,97E-01	421,68	2048,00	6,54E-01	1169,58	24154,97	9,83E-01
SPCC1450.12_b	5	254,23	372,22	306,55	337,79	8,97E-01	2856,44	735,72	2876,30	539,43	9,60E-01	278,20	415,87	7,45E-01	2896,31	614,48	9,82E-01	252,48	484,38	7,13E-01	2836,70	1077,64	9,18E-01
SPCC1450.12_d	8	458,25	192,67	464,65	238,86	8,94E-01	2861,53	3019,30	2874,57	3061,45	8,42E-01	404,50	268,73	9,47E-01	2894,93	3040,30	8,24E-01	407,31	252,48	9,80E-01	2831,99	2836,70	3,11E-01
SPCC1450.15_a	16	1964,57	481,04	2019,80	477,71	9,83E-01	10847,44	3028,36	10607,03	3059,55	9,86E-01	2401,97	471,14	8,77E-01	9310,85	3047,85	8,94E-01	4240,45	461,44	6,34E-01	9332,80	2838,92	8,82E-01
SPCC1494.02c_b	9	512,00	1675,06	474,41	1389,16	8,47E-01	9350,43	10506,15	8846,98	9795,96	5,02E-01	608,87	1509,65	9,67E-01	7814,55	9055,06	2,20E-01	689,78	3902,01	5,54E-01	7741,70	9865,68	4,50E-01
SPCC14G10.04_b	4	315,17	461,44	424,61	424,61	6,69E-01	19893,37	8753,22	20311,37	8362,44	9,99E-01	455,09	544,96	3,23E-01	19893,37	8174,14	9,75E-01	377,41	564,18	5,59E-01	19755,96	8095,36	9,65E-01
SPCC14G10.04_c	12	1152,06	393,44	1428,22	436,55	8,22E-01	19888,61	20031,74	20347,54	19215,73	7,84E-01	1488,87	410,15	8,14E-01	19951,64	18820,27	4,20E-01	1438,15	458,25	8,04E-01	19793,36	19619,49	1,53E-01
SPCC162.07_a	12	552,56	1243,34	729,11	1332,57	7,99E-01	25709,25	20052,27	25355,30	19277,22	9,04E-01	719,08	1060,11	9,85E-01	24322,43	18786,26	7,69E-01	781,44	1509,65	6,71E-01	23987,58	19555,02	7,87E-01
SPCC1620.01c_b	15	2164,77	675,59	1418,35	831,75	7,48E-01	14797,70	27746,15	12837,83	27175,14	9,08E-01	1910,85	814,63	9,56E-01	10307,60	26615,89	8,13E-01	1782,89	935,76	9,50E-01	11680,73	27939,14	9,01E-01
SPCC1620.02_a	9	401,71	1448,15	413,00	897,64	6,86E-01	5667,34	12694,36	4033,44	11582,86	8,15E-01	385,34	1120,56	8,13E-01	4840,09	10661,36	7,84E-01	572,05	1663,49	8,23E-01	7785,12	10963,76	9,65E-01
SPCC1620.05_d	4	249,00	380,04	216,77	430,54	9,49E-01	1585,19	6238,33	1568,60	3747,49	6,74E-01	313,00	413,00	6,16E-01	1687,77	3623,79	6,68E-01	284,05	685,02	5,05E-01	2088,21	7582,00	8,22E-01
SPCC1620.06c_b	6	6562,36	243,88	9089,59	259,57	8,37E-01	38165,99	1451,83	36865,92	1614,62	9,84E-01	8539,88	263,20	8,66E-01	32768,00	1777,98	9,26E-01	12677,65	242,19	7,04E-01	35610,13	1786,55	9,69E-01
SPCC1672.01_a	4	1234,75	5914,33	989,12	9280,58	7,74E-01	3917,24	37122,34	3400,37	36107,23	9,77E-01	996,00	7912,95	8,53E-01	3331,26	33456,53	9,33E-01	1710,26	14164,58	5,79E-01	4113,60	34397,12	9,60E-01
SPCC1672.01_b	7	1234,75	867,07	1052,79	982,29	8,75E-01	3917,24	3643,82	3400,37	3267,39	9,90E-02	1052,79	809,00	6,41E-01	3331,26	3432,79	1,12E-01	1379,57	1530,73	1,79E-01	4113,60	3670,72	7,10E-01
SPCC1672.04c_b	19	209,38	1060,11	261,38	1060,11	9,69E-01	335,46	3643,82	568,10	3267,39	9,76E-01	261,38	968,76	9,75E-01	481,04	3432,79	9,90E-01	232,32	1269,46	8,78E-01	560,28	3670,72	9,61E-01
SPCC1672.12c_c	4	396,18	212,31	337,79	270,60	1,00E+00	4564,55	461,44	5078,32	734,19	9,07E-01	388,02	245,57	9,24E-01	4938,39	504,95	9,51E-01	306,55	233,94	7,64E-01	4727,84	471,14	9,79E-01
SPCC1682.04_a	7	689,78	315,17	770,69	319,57	8,98E-01	7662,55	4391,21	7042,27	4756,57	9,55E-01	1031,12	407,31	6,12E-01	6237,13	5080,72	8,52E-01	1675,06	342,51	5,41E-01	7474,14	4389,18	9,70E-01
SPCC1682.07_a	5	249,00	458,25	385,34	487,75	5,50E-01	5480,15	4366,84	5955,47	4346,57	8,38E-01	430,54	675,59	3,41E-01	7434,40	3657,09	7,82E-01	372,22	1074,91	4,19E-01	5873,48	4485,69	8,01E-01
SPCC1682.09c_b	4	885,29	300,25	1722,16	377,41	5,97E-01	15935,98	5042,77	16270,83	5404,70	9,68E-01	2062,24	471,14	5,10E-01	14066,74	6562,36	9,81E-01	2665,15	369,65	5,17E-01	18053,61	5256,91	9,02E-01
SPCC1682.16_a	20	1243,34	975,50	1807,78	1468,37	1,34E-01	12677,65	13587,57	14066,74	14263,10	1,57E-01	2352,53	1640,59	1,45E-01	17559,94	11585,24	6,81E-01	1897,65	2062,24	3,11E-02	14066,74	16384,00	2,35E-01
SPCC16A11.03c_a	13	515,56	1009,90	398,93	1251,98	9,10E-01	3630,38	11910,94	3812,80	13400,51	9,07E-01	508,46	1820,35	6,24E-01	4156,88	16046,82	7,78E-01	588,13	1795,29	5,78E-01	4667,74	13587,57	8,44E-01

SPCC16A11.08_b	5	903,89	442,64	765,36	396,18	7,84E-01	3573,45	3173,00	3469,15	3458,43	6,95E-01	1009,90	430,54	9,11E-01	4346,95	3748,22	2,02E-01	1052,79	657,11	6,11E-01	3702,41	4126,56	2,05E-01
SPCC16A11.10c_b	23	576,03	749,61	600,49	754,83	9,10E-01	11068,16	3704,07	13854,70	3648,61	8,48E-01	560,28	916,51	7,40E-01	12586,72	3882,25	8,95E-01	530,06	786,88	9,80E-01	11565,77	3666,28	9,70E-01
SPCC16A11.12c_a	10	210,84	430,54	194,01	508,46	8,88E-01	2135,26	11393,61	2326,39	13766,72	8,78E-01	168,90	445,72	9,47E-01	2119,83	11748,95	9,82E-01	177,29	421,68	9,09E-01	1892,76	10196,03	9,18E-01
SPCC16A11.16c_b	7	194,01	174,85	203,66	165,42	9,96E-01	1542,43	2050,75	1265,04	2351,80	9,86E-01	272,48	181,02	4,61E-01	1864,45	2054,37	6,09E-01	216,77	191,34	3,43E-01	1239,65	1786,51	5,27E-01
SPCC16C4.01_d	4	304,44	210,84	324,03	192,67	9,94E-01	1929,93	1384,53	2235,89	1341,11	8,26E-01	259,57	184,82	6,14E-01	2610,47	1557,04	5,47E-01	221,32	213,78	4,83E-01	3344,46	1059,70	6,88E-01
SPCC16C4.03_a	6	929,30	245,57	1458,23	252,48	7,36E-01	15608,02	2062,62	15286,81	2191,88	9,93E-01	1910,85	296,11	6,16E-01	17438,64	2493,68	9,21E-01	2076,59	268,73	6,06E-01	14362,31	3090,35	9,91E-01
SPCC16C4.08c_c	4	187,40	948,83	194,01	1758,34	6,85E-01	7824,90	16728,26	8794,47	16270,83	9,69E-01	225,97	2076,59	6,19E-01	7692,75	16961,78	9,94E-01	235,57	2916,45	5,45E-01	9573,02	15181,22	9,86E-01
SPCC16C4.10_a	4	1910,85	286,03	1858,60	172,45	9,50E-01	23393,78	7399,73	22826,12	7869,54	9,97E-01	1964,57	215,27	9,95E-01	22058,13	6566,79	9,31E-01	2836,70	198,09	8,12E-01	21790,28	8484,80	9,82E-01
SPCC16C4.16c_a	5	487,75	1686,71	552,56	1807,78	9,24E-01	2276,72	20298,86	2186,54	20700,07	9,91E-01	526,39	1871,53	9,13E-01	1964,30	20257,16	9,90E-01	704,28	2272,40	7,24E-01	2186,63	19972,24	9,88E-01
SPCC16C4.18c_a	5	749,61	487,75	1209,34	458,25	6,43E-01	19483,97	2031,12	19619,49	1776,17	9,97E-01	837,53	508,46	8,20E-01	17928,91	1618,15	9,42E-01	1243,34	643,59	4,26E-01	18432,96	2260,28	9,76E-01
SPCC16C4.22_a	4	519,15	744,43	680,29	831,75	4,57E-01	356,21	19349,38	437,21	18432,96	9,77E-01	608,87	714,11	8,33E-01	431,84	17805,06	9,60E-01	820,30	1478,58	2,75E-01	529,10	19215,73	9,99E-01
SPCC1739.14_a	7	252,48	704,28	324,03	548,75	8,83E-01	9299,44	282,68	8752,22	429,11	9,77E-01	372,22	537,45	9,31E-01	8316,97	432,38	9,51E-01	413,00	820,30	6,94E-01	9370,59	447,01	9,87E-01
SPCC1739.15_a	11	564,18	313,00	630,35	266,87	9,68E-01	496,06	8618,39	424,78	8420,06	9,83E-01	797,86	319,57	7,00E-01	453,34	7908,52	9,52E-01	1052,79	372,22	5,29E-01	757,24	9326,87	9,42E-01
SPCC1753.01c_a	4	369,65	584,07	257,78	729,11	9,55E-01	8543,28	552,47	8648,15	421,45	9,98E-01	369,65	897,64	6,37E-01	7903,69	437,93	9,51E-01	464,65	1052,79	4,63E-01	8956,93	709,99	9,65E-01
SPCC1753.03c_a	4	210,84	390,72	306,55	247,28	8,25E-01	404,50	8268,52	1105,13	8533,78	9,37E-01	448,82	237,21	7,90E-01	1910,85	8040,82	9,10E-01	296,11	461,44	5,89E-01	962,07	8466,03	9,51E-01
SPCC1795.03_a	6	1184,45	213,78	1217,75	302,33	9,36E-01	25296,72	385,34	23257,74	1168,14	9,73E-01	1002,93	413,00	9,89E-01	19930,21	2304,12	9,20E-01	1351,18	367,09	8,38E-01	21794,18	962,07	9,36E-01
SPCC18.09c_a	9	512,00	903,89	430,54	968,76	9,82E-01	5267,32	22292,53	5782,12	21483,04	9,91E-01	508,46	867,07	9,46E-01	5972,74	18621,43	9,02E-01	530,06	1192,69	7,29E-01	6122,20	21470,67	9,99E-01
SPCC18.10_b	6	648,07	445,72	809,00	421,68	7,84E-01	4265,81	4736,63	4319,02	4893,90	8,04E-01	826,00	491,14	6,26E-01	3425,88	5191,19	8,52E-01	1031,12	608,87	3,64E-01	3935,26	5431,44	8,38E-01
SPCC18.12c_a	6	274,37	621,67	240,52	643,59	9,84E-01	796,68	4249,06	1158,03	3918,06	9,95E-01	247,28	826,00	8,17E-01	890,28	3355,11	8,68E-01	282,09	1168,14	6,19E-01	1172,87	4451,09	9,14E-01
SPCC18.12c_b	4	263,20	266,87	222,86	238,86	5,31E-02	796,68	767,80	1158,03	980,72	8,55E-02	210,84	227,54	3,31E-02	890,28	798,82	3,23E-01	190,02	222,86	7,11E-02	1172,87	1037,72	4,28E-02
SPCC18.14c_a	14	374,81	198,09	484,38	245,57	6,50E-01	34159,12	767,80	33791,50	980,72	9,98E-01	374,81	192,67	9,85E-01	27958,88	798,82	8,99E-01	427,57	195,36	8,80E-01	31216,78	1037,72	9,58E-01
SPCC18.15_b	20	1820,35	364,56	1782,89	393,44	9,97E-01	12765,24	32849,87	13071,01	33610,83	9,74E-01	1910,85	377,41	9,65E-01	13092,32	29656,23	9,22E-01	2225,63	481,04	8,40E-01	12131,10	31865,99	9,59E-01
SPCC18.18c_a	24	2556,58	1520,15	2574,36	1640,59	9,30E-01	30596,29	11307,88	29531,49	11904,32	9,87E-01	2998,45	1541,37	8,20E-01	29592,41	11139,47	9,69E-01	4124,49	2352,53	3,63E-01	29396,23	10500,76	9,48E-01
SPCC18.19c_a	6	181,02	2368,90	249,00	2435,50	9,69E-01	63,56	30887,29	78,79	30106,30	9,87E-01	254,23	3082,75	8,46E-01	81,57	30021,65	9,86E-01	250,73	4153,18	7,19E-01	131,60	29611,88	9,80E-01
SPCC1827.06c_a	14	464,65	178,53	477,71	195,36	9,47E-01	32061,42	38,85	30634,47	82,71	9,78E-01	390,72	237,21	9,67E-01	27230,53	83,87	9,20E-01	530,06	216,77	8,30E-01	30380,25	75,58	9,74E-01
SPCC1840.05c_a	19	349,71	458,25	404,50	344,89	6,83E-01	5892,93	30093,32	5725,11	30162,74	9,98E-01	352,14	354,59	4,50E-01	5056,99	28265,84	9,44E-01	369,65	504,95	7,38E-01	5828,67	31080,30	9,81E-01
SPCC1840.06_b	4	617,37	319,57	996,00	396,18	5,67E-01	24661,96	5048,29	23170,48	5222,71	9,65E-01	744,43	330,84	8,12E-01	22693,63	4213,83	9,27E-01	680,29	393,44	7,72E-01	23331,64	5141,21	9,67E-01
SPCC1840.12_a	4	501,46	600,49	410,15	580,04	6,27E-01	1208,83	23657,34	1111,07	23170,48	9,87E-01	385,34	600,49	6,72E-01	1264,78	23170,48	9,90E-01	352,14	831,75	8,83E-01	1481,98	25006,23	9,65E-01
SPCC188.02_c	14	413,00	461,44	410,15	278,20	3,17E-01	12598,90	1406,64	11780,40	1196,64	9,53E-01	439,59	359,54	5,05E-01	10927,34	1151,74	9,09E-01	458,25	377,41	7,21E-01	10443,26	1264,74	8,88E-01
SPCC188.06c_a	20	380,04	349,71	477,71	390,72	2,71E-01	7863,47	11680,16	8409,73	11112,57	9,97E-01	592,22	357,05	4,52E-01	8006,24	11035,40	9,27E-01	903,89	430,54	3,30E-01	7909,64	10462,49	8,22E-01

SPCC1884.01_a	8	187,40	382,68	152,22	424,61	9,86E-01	241,64	7533,94	206,46	7396,89	9,88E-01	199,47	512,00	7,38E-01	232,11	7013,42	9,62E-01	198,09	849,22	5,55E-01	428,02	8360,94	9,34E-01
SPCC1884.01_b	6	922,88	160,90	643,59	149,09	7,79E-01	241,64	264,16	206,46	221,98	1,06E-01	820,30	170,07	9,34E-01	232,11	213,83	1,75E-01	1448,15	199,47	7,37E-01	428,02	388,08	2,11E-02
SPCC1906.03_a	9	388,02	837,53	407,31	625,99	7,38E-01	2499,00	264,16	2224,70	221,98	9,26E-01	388,02	634,73	7,31E-01	2292,76	213,83	9,41E-01	572,05	1458,23	5,03E-01	4194,16	388,08	7,20E-01
SPCC1906.04_a	12	148,06	313,00	153,28	407,31	7,74E-01	888,02	2956,86	905,08	2362,61	8,41E-01	147,03	385,34	8,29E-01	1161,25	2098,73	8,21E-01	153,28	675,59	5,71E-01	1408,08	3536,84	7,47E-01
SPCC1919.01_a	4	709,18	145,01	1016,93	153,28	7,88E-01	4400,60	814,10	4312,50	882,10	9,97E-01	916,51	151,17	8,43E-01	3677,10	1052,45	9,23E-01	1478,58	164,28	6,37E-01	4745,22	1188,50	9,00E-01
SPCC1919.01_b	4	354,59	861,08	424,61	744,43	9,45E-01	4389,98	4212,40	4299,64	3798,12	4,43E-01	739,29	867,07	5,32E-01	3666,02	3544,29	2,31E-02	410,15	1488,87	6,24E-01	4737,79	4611,30	7,57E-02
SPCC1919.03c_c	7	1067,48	243,88	1060,11	324,03	9,53E-01	23470,34	4211,15	21887,32	3795,30	9,47E-01	1226,22	613,11	6,58E-01	22886,41	3541,14	9,68E-01	1418,35	385,34	7,45E-01	22681,50	4608,24	9,90E-01
SPCC1919.06c_d	16	436,55	975,50	533,74	1152,06	7,70E-01	3444,31	21339,52	4329,55	21059,89	9,83E-01	657,11	1031,12	7,15E-01	5792,62	21871,93	9,16E-01	765,36	1488,87	4,49E-01	6472,02	20124,50	9,43E-01
SPCC1919.09_b	11	330,84	451,94	372,22	588,13	5,48E-01	17598,01	3743,05	19710,61	4938,99	8,85E-01	433,53	739,29	3,57E-01	18721,69	5077,84	9,11E-01	481,04	760,08	2,71E-01	21316,01	6208,38	7,91E-01
SPCC1919.11_b	4	781,44	290,02	699,41	308,69	9,29E-01	592,22	17776,62	748,99	18886,33	9,64E-01	685,02	302,33	9,05E-01	879,37	18805,72	9,63E-01	781,44	418,77	8,53E-01	651,05	22046,70	8,89E-01
SPCC23B6.01c_a	15	369,65	1038,29	367,09	797,86	7,89E-01	20137,57	682,67	17666,33	757,00	9,34E-01	572,05	942,27	9,02E-01	20748,80	931,49	9,78E-01	484,38	1089,92	8,71E-01	21087,71	832,83	9,72E-01
SPCC23B6.01c_b	4	3040,30	317,37	2556,58	380,04	9,15E-01	20137,57	20763,21	17666,33	19394,02	1,72E-01	3213,66	541,19	9,27E-01	20748,80	21516,84	3,02E-01	3590,58	544,96	8,67E-01	21087,71	21134,99	1,70E-01
SPCC23B6.01c_c	4	2503,97	2896,31	1663,49	2435,50	2,72E-01	20137,57	20763,21	17666,33	19394,02	1,72E-01	2019,80	3590,58	9,09E-01	20748,80	21516,84	3,02E-01	2272,40	3666,02	7,46E-01	21087,71	21134,99	1,70E-01
SPCC24B10.03_a	8	152,22	2225,63	171,25	1488,87	7,98E-01	162,33	20763,21	221,09	19394,02	9,67E-01	186,11	1964,57	9,41E-01	193,95	21516,84	9,81E-01	196,72	2916,45	8,50E-01	384,01	21134,99	9,86E-01
SPCC24B10.05_a	10	257,78	162,02	261,38	164,28	9,70E-01	7184,35	160,08	7286,71	207,13	9,89E-01	261,38	156,50	9,90E-01	5783,60	162,67	8,91E-01	328,56	156,50	7,72E-01	7594,79	239,34	9,66E-01
SPCC24B10.06_a	7	501,46	195,36	515,56	227,54	9,22E-01	14107,12	6457,28	12506,59	7461,46	9,54E-01	544,96	216,77	8,98E-01	12928,43	6330,90	9,09E-01	560,28	292,04	7,39E-01	13721,37	7785,04	9,31E-01
SPCC24B10.14c_a	4	168,90	498,00	184,82	544,96	9,09E-01	219,47	12012,63	184,67	11165,96	9,61E-01	206,50	643,59	7,70E-01	322,91	11655,20	9,89E-01	286,03	548,75	7,29E-01	461,86	11384,09	9,83E-01
SPCC24B10.17_b	4	2998,45	179,77	6936,54	162,02	6,47E-01	13124,73	250,02	13969,57	226,24	9,69E-01	8023,41	149,09	6,11E-01	13034,07	240,53	9,96E-01	15181,22	215,27	5,07E-01	12677,65	360,64	9,87E-01
SPCC24B10.18_a	12	600,49	2610,30	861,08	6295,04	5,66E-01	942,27	10734,74	1618,00	12854,63	8,69E-01	820,30	5752,61	5,92E-01	1520,15	12330,98	8,95E-01	657,11	13682,08	4,87E-01	2352,53	11036,54	9,08E-01
SPCC24B10.19c_a	19	310,83	630,35	263,20	680,29	9,97E-01	4233,21	1067,48	3859,84	1652,00	9,61E-01	352,14	814,63	7,27E-01	3812,91	1351,18	9,76E-01	464,65	709,18	6,22E-01	4345,91	2164,77	7,83E-01
SPCC285.06c_d	15	354,59	270,60	393,44	263,20	8,58E-01	1414,05	4437,32	1055,52	3857,57	8,41E-01	467,88	326,29	4,13E-01	1324,75	3857,61	8,81E-01	564,18	388,02	2,36E-01	2085,30	4485,49	8,69E-01
SPCC285.06c_e	11	308,69	372,22	326,29	436,55	5,86E-01	1414,05	920,08	1055,52	1134,00	8,00E-01	377,41	556,41	3,14E-01	1324,75	994,26	9,82E-01	326,29	556,41	4,87E-01	2085,30	1674,10	1,57E-01
SPCC285.07c_f	12	148,06	317,37	153,28	324,03	9,65E-01	151,97	920,08	124,43	1134,00	8,97E-01	147,03	364,56	8,82E-01	145,95	994,26	9,58E-01	153,28	380,04	8,33E-01	192,70	1674,10	6,81E-01
SPCC285.09c_a	20	3082,75	145,01	2179,83	153,28	8,25E-01	15877,42	147,89	14061,42	130,85	9,38E-01	2256,70	151,17	8,42E-01	13860,10	150,06	9,32E-01	2778,33	164,28	9,49E-01	16495,84	159,79	9,80E-01
SPCC285.11_a	8	685,02	2019,80	837,53	1795,29	9,69E-01	13701,45	14388,66	12578,09	14156,11	5,13E-01	685,02	1924,14	9,63E-01	13709,32	14524,11	9,05E-01	1128,35	2665,15	6,46E-01	11327,01	15805,65	8,52E-01
SPCC285.16c_a	5	265,03	689,78	235,57	734,19	9,84E-01	2457,51	13908,07	2529,66	12624,90	9,44E-01	222,86	634,73	8,85E-01	3360,69	12283,42	9,65E-01	178,53	989,12	8,38E-01	2892,80	11638,42	9,10E-01
SPCC285.17_b	5	2740,08	176,07	5556,65	210,84	6,78E-01	19215,73	2755,90	20594,91	2541,38	9,66E-01	4211,15	243,88	7,76E-01	17682,08	3114,82	9,62E-01	4451,27	240,52	7,53E-01	17559,94	2783,05	9,48E-01
SPCC285.17_c	7	1833,01	3492,39	2320,15	5077,84	5,86E-01	19215,73	16961,78	20594,91	18820,27	3,76E-01	1584,71	3956,48	9,47E-01	17682,08	18053,61	8,64E-01	2179,83	5330,30	6,02E-01	17559,94	17559,94	6,85E-01
SPCC290.02_a	5	210,84	1323,37	294,07	1807,78	7,91E-01	2957,17	16961,78	2628,46	18820,27	9,50E-01	254,23	1746,20	8,26E-01	2836,70	18053,61	9,67E-01	393,44	1951,00	7,13E-01	3590,58	17559,94	9,56E-01
SPCC290.04_a	4	461,44	229,13	666,29	247,28	6,87E-01	19083,00	2574,36	19349,38	2352,53	9,99E-01	504,95	245,57	8,79E-01	17438,64	2225,63	9,37E-01	1278,29	256,00	5,05E-01	22073,07	2916,45	9,07E-01

SPCC297.04c_a	12	776,05	436,55	929,30	675,59	4,52E-01	2935,92	20311,37	3262,42	18561,17	9,57E-01	792,35	396,18	9,67E-01	2784,55	18820,27	9,51E-01	648,07	1562,89	4,14E-01	2927,93	25006,23	8,83E-01
SPCC306.05c_b	10	530,06	968,76	617,37	1136,20	7,44E-01	9308,79	3838,08	8240,88	3923,10	9,01E-01	504,95	1067,48	9,27E-01	8929,07	3764,42	9,57E-01	661,68	809,00	9,57E-01	9573,50	3539,12	9,97E-01
SPCC306.07c_a	9	148,06	477,71	170,07	592,22	8,23E-01	2738,11	8631,64	3127,49	7406,30	9,19E-01	165,42	458,25	9,97E-01	3036,36	7723,22	9,43E-01	173,65	744,43	7,01E-01	4156,01	8530,78	8,74E-01
SPCC306.10_a	12	200,85	167,73	206,50	163,14	9,86E-01	525,19	2778,42	440,82	2548,65	9,28E-01	270,60	163,14	6,21E-01	550,89	2601,24	9,65E-01	300,25	160,90	5,84E-01	935,89	3501,58	7,71E-01
SPCC306.10_b	7	272,48	213,78	304,44	199,47	8,97E-01	525,19	527,08	440,82	500,99	2,08E-01	247,28	290,02	5,55E-01	550,89	637,33	2,56E-01	315,17	298,17	1,73E-01	935,89	722,53	1,05E-01
SPCC31H12.05c_b	16	319,57	219,79	308,69	286,03	6,43E-01	9091,38	527,08	10364,43	500,99	9,33E-01	298,17	229,13	9,30E-01	7620,03	637,33	9,13E-01	424,61	308,69	3,32E-01	8886,94	722,53	9,99E-01
SPCC320.09_a	4	729,11	288,01	1209,34	354,59	6,27E-01	14362,31	7708,82	16961,78	7922,07	8,26E-01	929,30	337,79	7,67E-01	12161,22	5610,28	6,90E-01	1009,90	421,68	6,30E-01	12944,04	6932,28	8,29E-01
SPCC320.10_c	4	259,57	1031,12	302,33	1530,73	7,44E-01	10769,23	15393,14	10991,01	17559,94	7,94E-01	288,01	1060,11	9,63E-01	9427,56	12944,04	5,81E-01	300,25	1144,10	9,05E-01	10149,59	14066,74	7,79E-01
SPCC320.11c_a	6	178,53	298,17	245,57	298,17	6,59E-01	7082,29	9712,44	8841,04	10027,72	5,47E-01	199,47	337,79	7,72E-01	7486,11	9073,39	9,46E-01	272,48	342,51	4,24E-01	9741,98	9735,11	4,15E-01
SPCC320.11c_b	6	313,00	154,34	367,09	230,72	5,97E-01	7098,97	6427,31	8837,33	7590,61	1,77E-01	354,59	206,50	7,08E-01	7465,71	6295,04	8,78E-01	398,93	256,00	4,72E-01	9758,92	8841,04	4,68E-02
SPCC330.03c_b	6	2797,65	298,17	781,44	359,54	5,21E-01	6025,43	6420,73	4120,12	7585,65	8,52E-01	530,06	321,80	4,65E-01	2786,45	6296,03	4,41E-01	1379,57	349,71	6,63E-01	5497,93	8865,12	6,29E-01
SPCC338.05c_b	6	227,54	2134,97	333,14	719,08	5,70E-01	15179,45	6400,39	14696,99	3795,58	8,46E-01	259,57	484,38	4,88E-01	13344,73	3198,27	7,43E-01	349,71	1573,76	8,64E-01	14583,22	7473,83	9,70E-01
SPCC338.05c_c	7	203,66	219,79	238,86	218,27	3,27E-01	15179,45	15038,77	14696,99	16188,47	7,00E-01	190,02	288,01	6,38E-01	13344,73	14654,76	2,34E-01	209,38	292,04	4,52E-01	14583,22	13844,84	1,40E-01
SPCC338.15_a	5	282,09	200,85	284,05	192,67	9,64E-01	3815,47	15038,77	3752,59	16188,47	9,54E-01	249,00	256,00	8,12E-01	3386,71	14654,76	9,64E-01	321,80	209,38	7,61E-01	3434,31	13844,84	9,27E-01
SPCC417.09c_a	4	290,02	213,78	304,44	315,17	2,71E-01	3199,12	3731,84	2866,91	3591,57	6,52E-01	300,25	237,21	7,66E-01	3293,09	3316,24	6,08E-01	347,29	257,78	4,80E-01	3823,02	3326,58	7,92E-01
SPCC417.09c_c	4	190,02	340,14	229,13	302,33	9,95E-01	3199,12	3335,03	2866,91	3129,72	2,11E-01	252,48	298,17	9,08E-01	3293,09	3327,94	5,99E-01	170,07	326,29	8,90E-01	3823,02	3748,95	2,15E-02
SPCC4B3.02c_c	5	891,44	177,29	867,07	196,72	9,96E-01	14414,88	3335,03	12399,13	3129,72	8,92E-01	600,49	141,04	7,37E-01	8444,17	3327,94	6,73E-01	1024,00	156,50	9,30E-01	9055,00	3748,95	7,26E-01
SPCC4G3.02_a	4	203,66	666,29	304,44	634,73	9,14E-01	15608,02	12789,22	15935,98	11489,96	8,71E-01	165,42	634,73	9,25E-01	12077,21	7583,13	2,41E-01	216,77	1184,45	6,69E-01	12416,75	10517,67	2,49E-01
SPCC4G3.02_c	5	382,68	165,42	572,05	232,32	5,90E-01	15608,02	12245,81	15935,98	12677,65	8,86E-01	442,64	240,52	6,94E-01	12077,21	9607,86	2,77E-01	458,25	173,65	8,37E-01	12416,75	10155,69	3,22E-01
SPCC4G3.04c_a	5	1105,13	308,69	1884,54	552,56	5,77E-01	17928,91	12245,81	19349,38	12677,65	8,52E-01	2048,00	442,64	6,09E-01	18053,61	9607,86	8,28E-01	1618,00	439,59	6,95E-01	16158,44	10155,69	6,86E-01
SPCC4G3.05c_a	5	252,48	849,22	354,59	1573,76	6,04E-01	2105,58	17198,56	2916,45	18690,27	9,26E-01	410,15	2336,28	5,00E-01	4389,98	17559,94	9,07E-01	256,00	1573,76	6,65E-01	3019,30	16384,00	9,97E-01
SPCC548.02c_d	13	380,04	335,46	374,81	424,61	3,36E-01	71,87	2272,40	62,42	3281,18	8,22E-01	458,25	652,58	1,86E-01	72,50	4640,29	6,86E-01	512,00	278,20	7,83E-01	118,16	2916,45	8,64E-01
SPCC548.05c_a	5	151,17	362,04	216,77	404,50	7,39E-01	2418,67	77,89	2759,13	101,41	9,28E-01	187,40	474,41	7,17E-01	3640,70	68,95	8,03E-01	154,34	596,34	6,76E-01	3691,52	95,32	7,92E-01
SPCC550.11_a	14	639,15	157,59	648,07	198,09	9,47E-01	9562,19	2836,70	9560,57	3082,75	9,81E-01	770,69	210,84	8,26E-01	9419,68	3769,09	9,37E-01	744,43	219,79	8,36E-01	10024,71	3444,31	9,20E-01
SPCC553.02_d	8	604,67	455,09	719,08	491,14	6,36E-01	4694,23	9990,37	4714,02	10154,71	9,83E-01	765,36	652,58	1,96E-01	4321,82	9493,74	9,17E-01	1016,93	689,78	2,14E-01	3535,26	9824,32	8,87E-01
SPCC576.05_a	8	2721,15	596,34	2856,44	729,11	9,37E-01	8678,81	5351,88	8844,57	4512,46	9,13E-01	3191,46	724,08	8,71E-01	10967,70	3931,24	9,21E-01	2778,33	975,50	8,90E-01	9445,91	3460,83	8,85E-01
SPCC576.05_b	4	1573,76	2304,12	2288,20	2134,97	5,41E-01	8659,09	8195,32	8841,04	8494,83	4,93E-01	2320,15	2628,46	3,09E-01	10960,30	10515,92	1,88E-02	2368,90	2683,69	2,78E-01	9475,59	9136,19	9,24E-02
SPCC576.14_a	11	451,94	1530,73	724,08	2352,53	6,32E-01	6653,97	8192,00	7696,57	8480,89	5,21E-01	891,44	1871,53	6,46E-01	7231,10	10513,82	5,08E-01	1408,55	2385,37	3,39E-01	8364,13	9152,82	2,62E-01
SPCC576.16c_d	11	240,52	380,04	243,88	556,41	6,52E-01	443,09	6746,86	384,94	7281,40	9,64E-01	261,38	903,89	4,95E-01	417,77	6251,56	9,57E-01	362,04	1209,34	3,84E-01	891,84	7858,29	8,83E-01
SPCC576.17c_a	5	494,56	222,86	481,04	222,86	9,74E-01	1855,24	465,45	2026,04	446,58	9,49E-01	729,11	278,20	6,37E-01	3103,24	423,40	7,28E-01	675,59	354,59	5,35E-01	2205,03	874,65	7,31E-01

SPCC584.05_b	8	1120,56	588,13	2019,80	596,34	6,11E-01	5256,91	2622,39	5184,54	2894,96	9,59E-01	2210,26	680,29	5,42E-01	5220,60	3989,15	6,92E-01	2957,17	724,08	4,81E-01	5220,60	2342,62	9,43E-01
SPCC584.11c_a	19	1287,18	942,27	1509,65	1964,57	1,61E-01	26068,14	5293,48	26987,43	5077,84	9,84E-01	1251,98	2288,20	3,53E-01	23493,92	5330,30	9,35E-01	1370,04	2797,65	3,18E-01	24154,43	5404,70	9,55E-01
SPCC584.15c_b	19	556,41	770,69	548,75	1009,90	6,94E-01	11099,92	24833,50	12246,27	25180,16	9,44E-01	608,87	781,44	8,40E-01	12276,95	23657,34	1,00E+00	719,08	709,18	6,84E-01	12000,34	23493,92	9,83E-01
SPCC594.01_b	12	8248,98	404,50	7231,10	430,54	9,33E-01	21810,70	11765,96	21364,34	12199,63	9,99E-01	6746,86	448,82	8,98E-01	20966,76	12443,31	9,91E-01	8599,28	481,04	9,73E-01	19768,22	12022,38	9,01E-01
SPCC5E4.03c_d	9	1351,18	6841,04	1332,57	5792,62	8,94E-01	18681,35	21565,08	18540,76	21249,48	9,19E-01	1251,98	7082,29	9,87E-01	17746,54	22307,64	9,75E-01	1351,18	8964,45	8,42E-01	18450,52	20578,00	7,66E-01
SPCC61.01c_a	5	1217,75	1226,22	1758,34	1136,20	5,44E-01	11268,44	18884,61	8779,97	18063,65	8,09E-01	1296,13	1192,69	7,08E-01	7912,95	19153,15	8,41E-01	1448,15	1341,84	8,33E-02	8306,36	19557,63	8,82E-01
SPCC61.04c_b	10	203,66	1243,34	232,32	1686,71	8,16E-01	10671,95	11346,82	10289,69	9280,58	1,81E-01	250,73	1629,26	8,25E-01	10267,17	8306,36	2,39E-01	215,27	2556,58	6,57E-01	10706,31	8364,13	3,50E-01
SPCC613.08_b	4	377,41	195,36	491,14	237,21	6,68E-01	2333,81	9911,26	2554,94	10367,09	9,56E-01	410,15	200,85	9,03E-01	2390,53	9569,38	9,81E-01	522,76	235,57	6,40E-01	2643,88	10677,61	9,31E-01
SPCC622.11_a	8	613,11	374,81	670,92	344,89	9,51E-01	9507,89	1993,05	8403,66	2216,24	9,36E-01	533,74	461,44	9,79E-01	7802,96	2048,15	8,78E-01	689,78	445,72	7,07E-01	6862,92	2540,28	8,31E-01
SPCC622.14_a	13	1663,49	481,04	2019,80	421,68	8,95E-01	10884,59	8087,12	9280,58	7033,23	5,36E-01	3082,75	352,14	7,07E-01	6562,36	6883,52	1,89E-01	2856,44	544,96	6,76E-01	7967,99	6675,94	2,95E-01
SPCC622.17_a	11	333,14	1389,16	382,68	1845,76	8,05E-01	4022,11	8902,53	3650,94	8599,28	9,32E-01	494,56	2556,58	6,24E-01	4544,01	6038,61	6,91E-01	471,14	2486,67	6,42E-01	5131,12	6295,04	7,93E-01
SPCC622.21_a	10	213,78	319,57	207,94	364,56	8,55E-01	132,68	3506,91	130,05	2792,66	8,83E-01	186,11	382,68	8,88E-01	183,83	3613,28	9,77E-01	212,31	501,46	6,17E-01	189,82	4182,18	9,01E-01
SPCC622.21_b	13	410,15	196,72	357,05	192,67	8,52E-01	132,68	134,34	130,05	143,16	6,86E-01	498,00	216,77	7,89E-01	183,83	159,06	9,24E-02	458,25	196,72	9,00E-01	189,82	168,21	5,23E-02
SPCC63.07_b	4	215,27	310,83	224,41	319,57	9,07E-01	2639,06	134,34	2497,62	143,16	9,73E-01	206,50	347,29	8,86E-01	2678,95	159,06	9,87E-01	268,73	439,59	4,50E-01	2885,84	168,21	9,46E-01
SPCC663.15c_a	10	2194,99	263,20	2759,13	164,28	8,99E-01	7858,29	2176,36	7696,57	2242,74	9,91E-01	3019,30	215,27	8,41E-01	8841,04	2254,95	9,14E-01	4803,93	302,33	6,43E-01	7486,11	2468,99	9,93E-01
SPCC663.17_a	8	212,31	2759,13	167,73	3516,68	8,81E-01	119,29	8902,53	97,52	9026,81	9,94E-01	183,55	3492,39	8,82E-01	116,57	9607,86	9,62E-01	198,09	4705,07	7,45E-01	154,13	8306,36	9,67E-01
SPCC736.05_a	8	342,51	183,55	306,55	186,11	8,82E-01	902,75	120,39	918,98	125,86	9,86E-01	455,09	181,02	7,61E-01	1705,00	104,54	7,02E-01	388,02	176,07	8,99E-01	1788,07	114,08	6,81E-01
SPCC736.11_a	17	724,08	298,17	661,68	280,14	9,01E-01	3560,19	863,75	3345,72	935,17	9,72E-01	873,10	415,87	7,11E-01	4292,55	1531,98	7,51E-01	996,00	484,38	5,62E-01	4794,13	1333,67	7,35E-01
SPCC736.12c_d	5	873,10	770,69	948,83	770,69	7,48E-01	19645,39	4815,87	20629,48	4376,52	9,82E-01	1418,35	1089,92	1,29E-01	18669,89	5827,58	9,99E-01	1038,29	1168,14	7,66E-02	21757,56	6072,43	8,90E-01
SPCC736.14_a	11	657,11	709,18	749,61	781,44	1,14E-01	5832,91	19032,76	5556,65	19912,56	9,78E-01	873,10	1458,23	2,42E-01	5712,87	18730,48	9,84E-01	1478,58	942,27	1,89E-01	6295,04	20835,40	9,19E-01
SPCC737.06c_b	6	326,29	464,65	436,55	666,29	3,65E-01	6251,56	6427,31	5996,90	6122,90	1,23E-01	474,41	739,29	2,93E-01	5480,15	5556,65	1,34E-02	313,00	1296,13	4,97E-01	6653,97	6122,90	8,77E-01
SPCC737.06c_c	4	292,04	280,14	344,89	418,77	1,25E-01	6250,71	5752,61	6010,07	5442,30	5,42E-01	380,04	415,87	2,73E-02	5478,13	5595,30	2,11E-01	380,04	357,05	2,38E-02	6676,11	6700,25	1,10E-01
SPCC74.01_b	4	357,05	286,03	385,34	354,59	3,37E-01	7696,57	5770,04	8422,31	5445,86	9,20E-01	328,56	464,65	4,31E-01	7383,04	5609,61	8,73E-01	357,05	436,55	2,93E-01	7643,41	6710,53	7,19E-01
SPCC74.02c_b	4	1398,83	225,97	1509,65	337,79	9,05E-01	7716,02	7131,55	8902,54	7231,10	5,43E-01	1045,52	328,56	8,72E-01	10514,32	6793,79	5,81E-01	1160,07	380,04	9,58E-01	9514,70	7486,11	4,15E-01
SPCC74.02c_c	6	3169,41	1251,98	4576,41	1200,98	7,60E-01	7696,57	6449,41	8902,53	7172,15	4,61E-01	4705,07	1002,93	7,87E-01	10513,82	9229,01	8,89E-02	5595,30	1296,13	6,52E-01	9541,50	8210,74	1,87E-01
SPCC74.03c_b	8	380,04	2452,44	410,15	2797,65	9,16E-01	4451,27	6427,31	4672,57	7181,15	7,89E-01	608,87	3615,55	7,40E-01	5007,93	9216,48	5,46E-01	765,36	5673,41	5,68E-01	4359,66	8192,00	7,35E-01
SPCC757.08_b	4	203,66	304,44	315,17	415,87	2,58E-01	1341,84	4973,34	1746,20	6122,90	8,10E-01	247,28	491,14	4,75E-01	2048,00	5792,62	7,98E-01	354,59	608,87	2,38E-01	2225,63	4640,29	9,11E-01
SPCC777.06c_a	5	184,82	176,07	232,32	200,85	1,57E-01	10884,59	1341,84	11665,82	1686,71	9,42E-01	209,38	276,28	2,06E-01	9152,82	1782,89	9,25E-01	256,00	319,57	7,89E-02	12245,81	1978,24	9,00E-01
SPCC777.09c_a	11	861,08	144,01	837,53	184,82	9,87E-01	27633,57	9741,98	27397,56	10226,32	9,93E-01	803,41	167,73	9,75E-01	23073,13	7967,99	8,12E-01	1105,13	249,00	7,84E-01	26560,06	10441,20	9,89E-01
SPCC777.10c_a	10	313,00	744,43	249,00	648,07	8,11E-01	9709,08	25251,65	7262,69	26004,82	9,51E-01	218,27	916,51	9,34E-01	9037,00	22846,78	8,96E-01	292,04	1168,14	7,20E-01	9651,11	25086,56	9,93E-01

SPCC777.10c_b	5	300,25	272,48	410,15	230,72	7,43E-01	9741,98	10572,26	7281,40	7834,45	3,49E-02	481,04	280,14	4,51E-01	9026,81	10017,31	4,29E-01	630,35	362,04	2,60E-01	9674,69	11180,79	7,83E-01
SPCC777.12c_a	4	380,04	294,07	1009,90	367,09	3,92E-01	8192,00	10586,95	9345,14	7858,29	6,32E-01	685,02	424,61	2,53E-01	10586,95	10015,87	5,36E-01	477,71	564,18	9,46E-02	8192,00	11190,60	8,89E-01
SPCC777.14_a	4	162,02	427,57	150,12	814,63	6,52E-01	2690,62	7434,40	3087,40	8248,98	8,79E-01	112,99	639,15	8,09E-01	3264,35	8964,45	8,03E-01	146,02	430,54	9,76E-01	3367,64	7181,15	9,51E-01
SPCC777.17c_a	5	198,09	187,40	179,77	104,69	3,14E-01	811,19	2466,14	781,01	2865,89	9,02E-01	207,94	138,14	6,33E-01	820,78	2920,55	8,78E-01	212,31	139,10	6,90E-01	1049,61	3035,35	7,84E-01
SPCC790.03_b	6	1067,48	183,55	1067,48	237,21	9,69E-01	7192,18	856,00	5036,31	676,31	7,90E-01	1260,69	171,25	9,09E-01	6559,32	621,20	9,30E-01	1499,22	216,77	7,93E-01	5709,15	701,73	8,58E-01
SPCC794.02_a	12	504,95	962,07	584,07	1016,93	8,51E-01	1217,75	6306,86	1389,16	4884,35	8,58E-01	704,28	1168,14	5,97E-01	1323,37	5899,11	9,69E-01	903,89	1089,92	3,98E-01	1937,53	4942,35	9,23E-01
SPCC825.02_d	4	484,38	504,95	568,10	643,59	1,05E-01	14466,89	1184,45	13659,91	1499,22	9,81E-01	501,46	765,36	4,04E-01	11934,27	1897,65	9,23E-01	474,41	903,89	4,61E-01	12078,42	1964,57	9,32E-01
SPCC830.02_a	14	265,03	352,14	278,20	421,68	6,71E-01	2583,01	13949,39	1783,48	12450,66	8,96E-01	328,56	372,22	4,81E-01	2391,41	11534,44	8,75E-01	344,89	352,14	4,57E-01	3549,89	12010,69	9,52E-01
SPCC830.05c_a	22	1746,20	265,03	1675,06	265,03	9,75E-01	9341,36	2854,95	7888,99	1735,82	8,01E-01	1438,15	344,89	9,13E-01	8386,95	1963,31	8,58E-01	1418,35	342,51	9,04E-01	8905,69	3332,12	9,97E-01
SPCC830.06_a	8	504,95	1360,57	765,36	1112,82	9,90E-01	11505,21	8971,41	11746,96	7293,69	8,06E-01	861,08	1360,57	7,54E-01	9741,98	8606,56	5,24E-01	1192,69	1584,71	4,35E-01	11036,54	9193,05	9,44E-01
SPCC830.08c_b	5	792,35	467,88	797,86	719,08	5,22E-01	29677,55	12416,75	28985,68	11268,44	9,47E-01	724,08	634,73	7,97E-01	30862,97	9280,58	9,50E-01	699,41	1192,69	3,97E-01	27384,05	10586,95	8,80E-01
SPCC830.10_a	4	224,41	699,41	227,54	648,07	9,46E-01	3322,99	25657,10	2923,36	26349,35	9,94E-01	225,97	729,11	9,68E-01	2497,55	29745,12	9,35E-01	261,38	820,30	8,50E-01	3786,39	26091,49	9,80E-01
SPCC830.10_c	6	367,09	178,53	330,84	233,94	9,36E-01	3322,99	2882,94	2923,36	2973,65	5,58E-01	300,25	191,34	8,27E-01	2497,55	2847,14	2,65E-01	374,81	252,48	7,51E-01	3786,39	3799,92	8,84E-02
SPCC895.04c_b	8	249,00	278,20	418,77	445,72	1,36E-02	6700,25	2882,94	6936,54	2973,65	9,58E-01	319,57	333,14	6,00E-02	6936,54	2847,14	9,75E-01	347,29	508,46	1,83E-01	5792,62	3799,92	9,98E-01
SPCC962.01_a	8	1964,57	250,73	2486,67	347,29	8,42E-01	9504,27	5914,33	9714,46	6038,61	9,54E-01	2062,24	215,27	9,83E-01	10064,55	6472,02	8,46E-01	2628,46	324,03	8,21E-01	10294,43	5256,91	9,85E-01
SPCC962.05_a	5	729,11	2225,63	922,88	2019,80	9,95E-01	6208,38	10389,42	6208,38	10217,01	9,79E-01	639,15	2005,85	8,93E-01	4299,64	11090,03	8,93E-01	734,19	3040,30	7,94E-01	5832,91	11403,28	9,35E-01
SPCC965.03_b	12	962,07	639,15	1031,12	955,43	3,65E-01	16092,91	6427,31	15810,36	6338,83	9,81E-01	1209,34	680,29	6,87E-01	15238,93	4067,71	8,48E-01	1160,07	982,29	2,80E-01	13532,37	6295,04	8,44E-01
SPCC965.04c_a	4	247,28	910,17	388,02	885,29	9,02E-01	3798,50	15879,07	3776,27	14543,69	9,41E-01	367,09	942,27	8,79E-01	3418,53	15440,61	9,66E-01	342,51	1370,04	6,94E-01	3678,77	15162,25	9,65E-01
SPCC970.04c_a	19	1418,35	324,03	1370,04	278,20	9,57E-01	16322,40	3574,75	12989,85	3547,51	8,52E-01	1217,75	319,57	8,98E-01	12512,86	2981,75	8,08E-01	1184,45	372,22	9,04E-01	13525,18	3411,30	8,72E-01
SPCC970.05_a	6	313,00	942,27	278,20	942,27	9,73E-01	24679,49	13604,18	23760,67	11453,30	8,70E-01	315,17	719,08	7,95E-01	20447,09	10803,50	6,79E-01	401,71	809,00	9,58E-01	23296,01	10664,47	8,21E-01
SPCC970.11c_a	12	504,95	294,07	576,03	274,37	9,02E-01	3142,90	23393,36	2365,12	23463,66	9,83E-01	699,41	326,29	6,50E-01	1908,78	19382,47	8,63E-01	897,64	357,05	5,15E-01	3751,94	20789,03	9,47E-01
SPCP1E11.04c_a	10	879,17	533,74	1136,20	643,59	6,04E-01	27175,14	2883,29	26987,43	1942,59	9,77E-01	1009,90	770,69	4,74E-01	25180,16	1727,17	9,34E-01	1038,29	910,17	2,83E-01	25355,30	3218,15	9,68E-01
SPCP1E11.08_b	5	337,79	765,36	315,17	962,07	8,43E-01	3242,68	26249,46	3506,68	25180,16	9,82E-01	313,00	843,36	9,45E-01	3331,27	25709,25	9,90E-01	328,56	1192,69	7,07E-01	4572,81	25531,66	9,86E-01
SPCP1E11.09c_a	6	471,14	321,80	448,82	377,41	8,59E-01	18390,65	2959,01	19243,17	3342,91	9,61E-01	347,29	326,29	5,12E-01	15267,51	2777,50	8,83E-01	522,76	359,54	7,25E-01	16604,26	4022,52	9,74E-01
SPCP31B10.08c_a	7	280,14	544,96	250,73	474,41	8,00E-01	28619,22	17559,68	26241,51	18267,28	9,14E-01	242,19	335,46	4,71E-01	24320,96	14394,02	6,65E-01	249,00	522,76	9,01E-01	28809,17	15887,78	9,39E-01
SPCPB16A4.02c_c	12	1897,65	227,54	1951,00	218,27	9,87E-01	12835,05	25275,45	11712,30	25061,43	9,48E-01	2033,85	222,86	9,62E-01	10625,47	20694,56	7,13E-01	2936,74	292,04	7,58E-01	11311,16	23265,10	8,57E-01
SPCPB16A4.04c_a	5	410,15	1499,22	560,28	1606,83	8,80E-01	4269,94	11277,43	5184,54	10237,70	9,90E-01	592,22	1820,35	7,88E-01	4640,29	10186,28	9,43E-01	709,18	2435,50	6,07E-01	4870,99	10224,70	9,64E-01
